# Supramolecular Functionalizable Linear–Dendritic Block Copolymers for the Preparation of Nanocarriers by Microfluidics

**DOI:** 10.3390/polym13050684

**Published:** 2021-02-25

**Authors:** Miriam Abad, Alejandro Martínez-Bueno, Gracia Mendoza, Manuel Arruebo, Luis Oriol, Víctor Sebastián, Milagros Piñol

**Affiliations:** 1 Instituto de Nanociencia y Materiales de Aragón (INMA), CSIC-Universidad de Zaragoza, 50009 Zaragoza, Spain; abadm@unizar.es (M.A.); amartinezb@unizar.es (A.M.-B.); gmendoza@issaragon.es (G.M.); arruebom@unizar.es (M.A.); loriol@unizar.es (L.O.); 2 Departamento de Química Orgánica, Facultad de Ciencias, Universidad de Zaragoza, Pedro Cerbuna 12, 50009 Zaragoza, Spain; 3 Networking Research Centre on Bioengineering, Biomaterials and Nanobiomedicine (CIBER-BNN), 28029 Madrid, Spain; 4 Aragon Health Research Institute (ISS Aragón), 50009 Zaragoza, Spain; 5 Department of Chemical Engineering and Environmental Technologies, University of Zaragoza, 50018 Zaragoza, Spain

**Keywords:** linear–dendritic block copolymers, self-assembly, polymeric nanocarriers, microfluidics

## Abstract

Hybrid linear–dendritic block copolymers (LDBCs) having dendrons with a precise number of peripheral groups that are able to supramolecular bind functional moieties are challenging materials as versatile polymeric platforms for the preparation of functional polymeric nanocarriers. **PEG_2k_-*b*-dxDAP** LDBCs that are based on polyethylene glycol (PEG) as hydrophilic blocks and dendrons derived from bis-MPA having 2,6-diacylaminopyridine (DAP) units have been efficiently synthesized by the click coupling of preformed blocks, as was demonstrated by spectroscopic techniques and mass spectrometry. Self-assembly ability was first checked by nanoprecipitation. A reproducible and fast synthesis of aggregates was accomplished by microfluidics optimizing the total flow rate and phase ratio to achieve spherical micelles and/or vesicles depending on dendron generation and experimental parameters. The morphology and size of the self-assemblies were studied by TEM, Cryogenic Transmission Electron Microscopy (cryo-TEM), and Dynamic Light Scattering (DLS). The cytotoxicity of aggregates synthesized by microfluidics and the influence on apoptosis and cell cycle evaluation was studied on four cell lines. The self-assemblies are not cytotoxic at doses below 0.4 mg mL^−1^. Supramolecular functionalization using thymine derivatives was explored for reversibly cross-linking the hydrophobic blocks. The results open new possibilities for their use as drug nanocarriers with a dynamic cross-linking to improve nanocarrier stability but without hindering disassembly to release molecular cargoes.

## 1. Introduction

The self-assembly ability of block copolymers (BCs) either in bulk or in solution has been widely explored to produce nanomaterials of interest because of the capability of chemically connected dissimilar polymers to phase segregate into self-organized structures of nanometric dimensions [1]. If the constituent blocks differ in regard to the solubility in a given solvent, as in the case of amphiphilic BCs in water, phase segregation occurs, resulting in self-assemblies whose morphology and size mainly depend on their macromolecular chemical structure, their hydrophilic/hydrophobic ratio, or the experimental assembly methodology followed [2]. Polymeric micelles, mainly spherical ones, and polymersomes have received particular attention as nanocarriers of active molecules. In comparison to low molecular weight amphiphiles, BC self-assemblies have higher stability and chemical tunability that can provide these polymeric nanocarriers with several functions such as stimuli-response, active targeting, or labeling, amongst others [3].

On the other hand, the emergence of dendritic macromolecules has opened new possibilities for designing materials of interest in nanobiomedicine [4]. Applications are mainly associated to the monodisperse, highly branched, and regular structure of dendrimers able to encapsulate molecules into the inner cavities of these macromolecules. They also have a high and well-defined number of peripheral end groups in which functionality such as drug conjugation, fluorescent probes, or DNA-binding groups, amongst others, can be incorporated. Furthermore, the particular physicochemical and self-assembly properties of this type of macromolecular architecture also offer potential applications as nanoreactors, in electronics and energy harvesting among other applications [5].

Hybrid linear–dendritic block copolymers (LDBCs), which contain two chemically connected blocks of different chain topology, may offer synergistic properties by combining the processability and easier preparation of linear polymers with the advantages of the regular branched structure of dendrons. From the point of view of the synthesis, different approaches have been exploited to prepare LDBCs in a precise manner [6]. LDBCs can be accomplished by a sequential synthesis of the blocks, using either a linear-chain first or a dendron-first approach, or by the separate synthesis of the linear and dendronized blocks and their final coupling. This coupling strategy is particularly attractive because it allows a better control over the length and dispersity of the blocks but requires very efficient reactions with a high tolerance to the presence of functional groups. In fact, LDBCs are usually synthesized by click chemistry reactions between preformed blocks having complementary reactive groups [7], i.e., an ending group in a linear block and the complementary one in the focal point of a preformed dendron [8,9].

The functionalization, response ability of LDBCs, and their applications in materials science were recently reviewed by Blasco et al. [10]. The manifest versatility of this architecture has lately been exploited by Malkoch and coworkers for the preparation of functional porous membranes using a series of 2,2-bis(hydroxymethyl)propionic acid (bis-MPA)-based dendrons [11,12]. Gitsov and coworkers have also described porous films and solution aggregates with an adjustable morphology, e.g., by Pd complexation, showing their potential applications as catalysts [13,14].

Nevertheless, most of the reported applications of functional LDBCs have been addressed to the nanobiomedicine field [15,16] and in particular to the preparation of aqueous self-assemblies [17]. LDBC self-assemblies exhibit high potential as nanocarriers of bioactive substances for drug and gene delivery as one of the most representative applications [18]. The adequate design of the block components and the selection of the experimental self-assembly conditions facilitate the control over the morphology and performance of the nanocarriers, as well as over their stability and dimensions, which are of crucial importance for their use in macromolecular therapeutics [19]. The effect of the dendritic macromolecular architecture on the self-assembly of LDBCs [20] has recently been theoretically examined [21]. In the case of amphiphilic LDBCs with hydrophobic dendrons, the dendron generation has a strong influence on the micelle morphology [22]. This fact was also earlier described in a series of LDBCs consisting of polyethylene glycol (PEG) and bis-MPA-derived dendrons with peripheral light responsive moieties synthesized in our labs [23,24]. They have been investigated as promoters of LDBC self-assembled nanocontainers exhibiting a light-controlled release of molecular payloads [25].

Since bis-MPA has widely demonstrated its synthetic versatility for the preparation of biocompatible and biodegradable peripherally functionalized dendrons [26], many of the examples in the literature have been focused on amphiphilic LDBCs consisting of a hydrophilic PEG segment linked to a hydrophobic bis-MPA based dendron. Recently, Malkoch and coworkers have described LDBCs consisting of a fluorescent-labeled PEG linked to a bis-MPA dendron with peripheral cholesterol units that exhibit improved drug-loading capacity and therapeutic efficacy [27]. Ambade and coworkers have described the preparation of smart LDBCs by click coupling PEG to dendrons incorporating photo- and pH-cleavable groups at the junction between the blocks as a strategy to enhance the stimulated release of doxorubicin (DOX) encapsulated in the micelles [28]. In a similar way, Sierra and coworkers have described plitidepsin nanocarriers [29]. Grayson and coworkers have also explored different branched macromolecular architectures based on PEG and bis-MPA dendrons with fatty acid chains at the periphery as nanocarriers for transdermal drug delivery [30,31]. In addition to bis-MPA based dendrons, relevant examples can be found with other dendrons, e.g., Amir and coworkers have studied the self-assembly properties of LDBCs with PEG and dendrons prepared by thiol-ene reaction with enzyme-responsive peripheral groups [32,33]. In the field of RNA therapeutics, Siegwart and coworkers have described a series of PEG-containing LDBCs to optimize the in vitro and in vivo siRNA delivery based on dendrimer–lipid nanoparticles as carriers [34,35]. From the described examples, it is clear that PEG has been mostly used as a linear block for the preparation of amphiphilic LDBCs [15] due to its biocompatibility and properties as a stealth coating polymer for biomedical applications [36,37]. However, alternatives such as poly[*N*-(2-hydroxyethyl-l-glutamine)] [38] or poly(*N*-vinylpyrrolidone) [39] have also been investigated for this purpose.

**Scheme 1:**
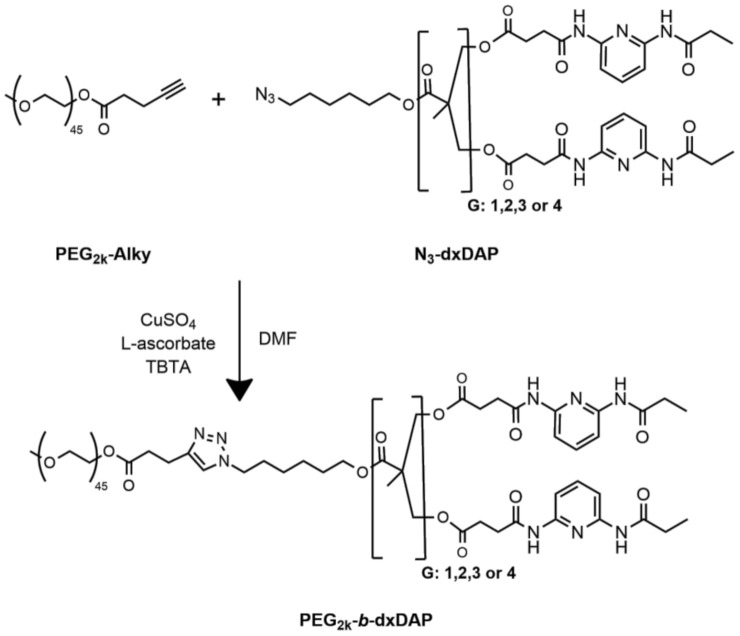
Synthesis of **PEG_2k_-*b*-dxDAP** (x = 2, 4, 8, or 16) linear–dendritic block copolymers (LDBCs) by copper(I)-catalyzed azide-alkyne cycloaddition (CuAAC) reaction (G = dendron generation. Number of peripheral ending groups = 2^G^). PEG_2k_: Polyethylenglycol monomethyl ether.

In a previous work, we described the performance of a series of amphiphilic linear–linear block copolymers based on PEG and a hydrophobic polymethacrylate chain containing side 2,6-diacylaminopyridine (DAP) units. These BCs easily self-assemble into stable and non-cytotoxic micelles sensitive to pH that are able to encapsulate hydrophobic drugs, as demonstrated with camptothecin [40]. DAP, a nucleobase analog, can also interact with complementary thymine, which envisages the possibility of functionalizing the DAP-containing block by H-bond recognition. Using this supramolecular approach for post-polymerization functionalization, nanocarriers with light responsiveness were provided [41]. In the present work, we report on a new series of LDBCs based on PEG and a bis-MPA dendron having peripheral DAP units. The LDBCs, coded as **PEG_2k_-*b*-dxDAP** (where x is the number of peripheral DAP moieties), have been synthesized by clicking two performed blocks and using the first four generations of bis-MPA dendrons (G = 1, 2, 3, or 4) with x = 2, 4, 8, or 16 DAP units, respectively (Scheme 1). Together with their characterization, the self-assembly properties of the LDBCs using two different methodologies, nanoprecipitation [42,43,44,45] and microfluidics, are described. Microfludics provide controlled reaction conditions and then an excellent control on the assembly of organic nanomaterials due to their remarkable heat and mass transfer enhancement [46]. In addition, microfluidics offers a reproducible and continuous operation, and it is also possible to adapt the production throughput by increasing the number of microfluidic units. The morphology and size of the self-assemblies have been determined by Transmission Electron Microscopy (TEM) and Dynamic Light Scattering (DLS), and their cytotoxicity has been evaluated in different cell lines. Furthermore, the supramolecular cross-linking of the self-assemblies has been tentatively explored by using cross-linkers containing several thymine units able to be H-bonded to the peripheral DAP moieties as a means to demonstrate the versatility of DAP units on providing functionality in a dynamic way.

## 2. Materials and Methods

### 2.1. General Synthetic Procedures

#### 2.1.1. Materials

**Scheme 2:**
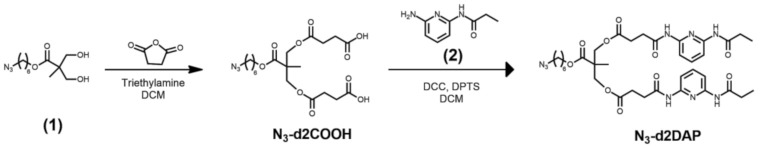
Synthesis of first-generation dendron, **N_3_-d2DAP**.

**Scheme 3:**
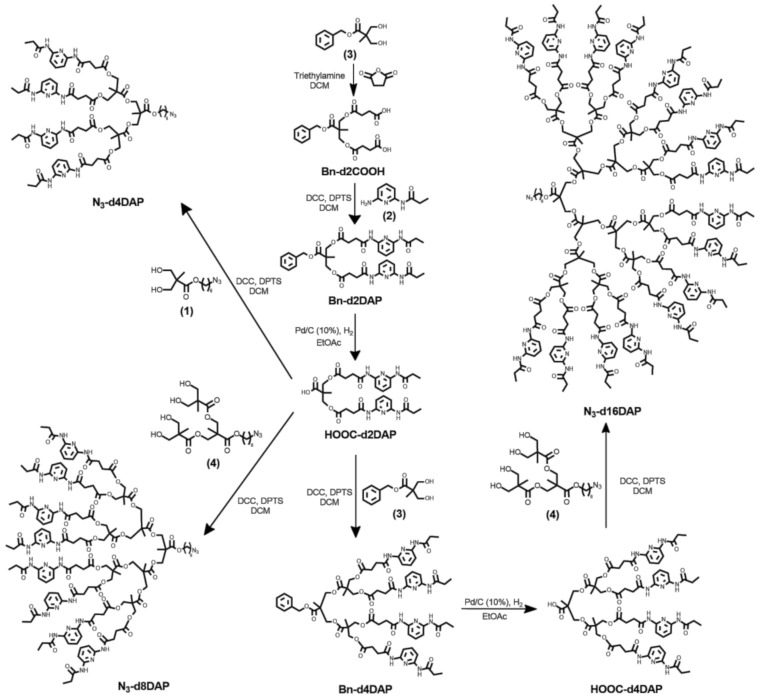
Synthesis of second (**N_3_-d4DAP**), third (**N_3_-d8DAP**), and fourth (**N_3_-d16DAP**) generation dendrons.

**Scheme 4:**
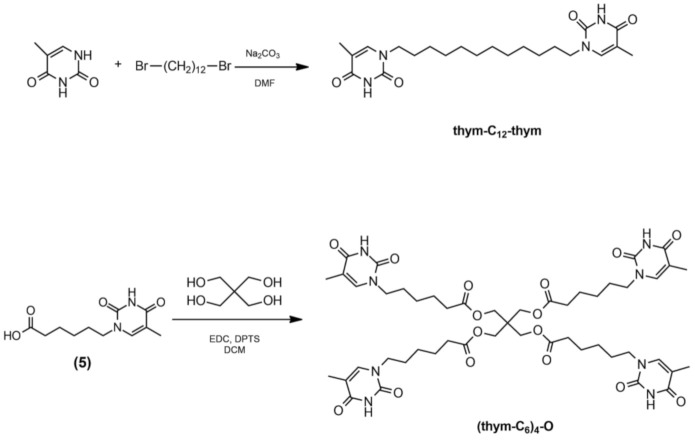
Synthesis of cross-linking agents with two (**thym-C_12_-thym**) and four (**(thym-C_6_)_4_-O**) thymine units.

The synthesis of the dendrons **N_3_-dxDAP**, where x represents the number of peripheral DAP units, is outlined in Scheme 2; Scheme 3, and the synthesis of thymine cross-linkers is outlined in Scheme 4. Compounds **(1)-(5)** were synthesized according to previously reported procedures [47,48,49]. Polyethylenglycol monomethyl ether (PEG_2k_–OH) was purchased from Sigma Aldrich (Sigma Aldrich Gmbh, Steinheim, Germany) and used as received (according to certificate of analysis, batch 1257344: M_n_ = 1678 g mol^−1^, *Ð* = 1.21; according to MALDI-TOF mass spectrometry: M_n_ = 1948.0 g mol^−1^ corresponding to the more intense *m*/*z* peak) (Figure S1). Palladium on carbon (Pd/C) (10 wt% loading, matrix-activated carbon support) catalyst was purchased from Sigma Aldrich. Tris[(1-benzyl-1H-1,2,3-triazol-4-yl)methyl]amine (TBTA) was purchased from ACROS Organics (Thermo Fisher Scientific, Geel, Belgium). The azide-functionalized Merrifield’s resin was prepared from Merrifield’s resin cross-linked with 1% divinylbenzene (200–400 mesh) (0.8–1.3 mmol g^−1^) (TCI Europe N.V., Zwijndrecht, Belgium) according to the literature procedure [50]. Other reagents were purchased from Sigma Aldrich and used as received. Fourier Transformed Infrared Spectroscopy (FTIR) and 1H NMR spectra are collected at the Supporting Information (Figures S2–S35).

#### 2.1.2. Synthesis of N_3_-d2COOH and Bn-d2COOH

**N_3_-d2COOH and Bn-d2COOH** were synthesized according to the following general procedure: Triethylamine (36.0 mol) was dropwise added to a solution of the selected dihydroxyl compound (6.0 mol) and succinic anhydride (36.0 mol) in dry dichloromethane (DCM) (25 mL) under Ar atmosphere. After stirring overnight at room temperature, the reaction crude was diluted with DCM and washed with 1 M HCl (aq) and water, dried over anhydrous MgSO_4_, and concentrated under vacuum. The resulting oil was diluted with diethyl ether and cooled at 0 °C for 3 h. Solid impurities were removed upon filtration, and the solvent evaporated under vacuum. The isolated yellow oil was purified by flash column chromatography on silica gel.

**N_3_-d2COOH** was obtained as described above from 6-azidohexyl 2,2-di(hydroxymethyl)propanoate **(1)**. The resulting yellow oil was purified by flash column chromatography eluting with ethyl acetate (EtOAc)/hexane (1:1) and gradually increasing the polarity to pure EtOAc. **N_3_-d2COOH** was obtained as a colorless viscous oil. Yield: 74%.

FTIR (KBr), ν (cm^‒1^): 3566, 2099, 1740.

^1^H NMR (400 MHz, CDCl_3_) δ (ppm): 4.32 (d, *J* = 11.0 Hz, 1H), 4.20 (d, *J* = 11.0 Hz, 1H), 4.13 (t, *J* = 6.6 Hz, 1H), 3.28 (t, *J* = 6.8 Hz, 1H), 2.72–2.57 (m, 4H), 1.70–1.56 (m, 2H), 1.46–1.32 (m, 2H), 1.24 (s, 2H).

**Bn-d2COOH** was obtained from benzyl 2,2-di(hydroxymethyl)propanoate **(3)**. The recovered oil was purified by flash column chromatography using first EtOAc/hexane (3:7) as eluent and gradually increasing polarity to EtOAc/hexane (6:4). **Bn-d2COOH** was obtained as a colorless viscous oil. Yield: 84%.

FTIR (ATR), ν (cm^‒1^): 3441, 1714.

^1^H NMR (400 MHz, CDCl_3_) δ (ppm) 9.41 (s, 2H), 7.38–7.28 (m, 5H), 5.15 (s, 2H), 4.29 (d, *J* = 11.2 Hz, 2H), 4.21 (d, *J* = 11.2, 2H), 2.61–2.51 (m, 8H), 1.24 (s, 3H).

^13^C NMR (100 MHz, CDCl_3_) δ (ppm): 177.9, 172.7, 171.7, 135.7, 128.7, 128.5, 128.3, 67.0, 65.6, 46.4, 28.9, 28.8, 18.0.

MS (HR-ESI^+^, *m*/*z*): calculated: 424.1; found: 447.1 [M + Na]^+^.

#### 2.1.3. Synthesis of DAP Functionalized Dendrons N_3_-dxDAP and Their Precursors

Synthesis was based on the following general esterification or amidation synthetic procedure. A solution of the selected acid (2.0 mol), the selected alcohol or amine (2.2 mol), and 4-(dimethylamino)pyridinium 4-toluenesulfonate (DPTS) (0.8 mol) in dry DCM (10–20 mL) was prepared under Ar atmosphere. The solution was cooled to 0 °C and *N,N*′-dicyclohexylcarbodiimide (DCC) (1.5 mol) was added. The mixture was stirred first at 0 °C for 1 h and then at room temperature for 48–72 h. The solid was filtered off, and the solvent was removed under vacuum. The crude product was purified by flash column chromatography on silica gel.

##### Synthesis of DAP Precursors HOOC-dxDAP

**Synthesis of Bn-d2DAP**. The compound was obtained according to the general amidation procedure described above from **Bn-d2COOH** and 2-amino-6-propanoylamidopyridine **(2)**. The crude product was purified by flash column chromatography using EtOAc/hexane (6:4) as eluent first and then gradually increasing the polarity to EtOAc/hexane (9:1) to yield the target compound as a white solid. Yield: 86%.

FTIR (ATR), ν (cm^‒1^): 3303, 1732, 1678, 1583, 1510, 1442, 1290, 1242, 1149.

^1^H NMR (400 MHz, CDCl_3_) δ (ppm) 8.18 (s, 2H), 8.03 (s, 2H), 7.89 (d, *J* = 7.9 Hz, 2H), 7.79 (s, 2H), 7.64 (t, *J* = 8.1 Hz, 2H), 7.37–7.27 (m, 5H), 5.13 (s, 2H), 4.33 (d, *J* = 11.1 Hz, 2H), 4.24 (d, *J* = 11.1 Hz, 2H), 2.72–2.50 (m, 8H), 2.37 (q, *J* = 7.5 Hz, 4H), 1.23 (s, 3H), 1.20 (t, *J* = 7.6 Hz, 6H).

^13^C NMR (100 MHz, CDCl_3_) δ (ppm): 172.7, 172.6, 172.4, 170.1, 149.9, 149.5, 140.9, 135.6, 128.8, 128.6, 128.3, 109.8, 109.6, 67.1, 65.4, 46.5, 33.4, 32.0, 30.8, 29.2, 25.4, 24.8, 18.1, 9.5.

MS (HR-ESI^+^, *m*/*z*): calculated: 718.3; found: 719.3 [M + H]^+^, 741.3 [M + Na]^+^.

**Synthesis of HOOC-d2DAP**. Carbon-supported palladium catalyst (0.770 g) was added to a solution of **Bn-d2DAP** (7.70 g, 10.7 mmol) in EtOAc (150 mL). The flask was evacuated from air and filled with hydrogen. After stirring for 24 h at room temperature, the catalyst was filtered off using Celite^®^ and carefully washed with EtOAc. The collected organic extracts were combined and evaporated, and the product was obtained as a white solid. Yield: 93%.

FTIR (KBr), ν (cm^‒1^): 3492, 3292, 1728, 1680, 1583, 1518, 1444, 1290, 1242, 1149.

^1^H NMR (400 MHz, CDCl_3_) δ (ppm) 8.70 (s, 2H), 8.41 (s, 2H), 7.75–7.61 (m, 4H), 7.49 (t, *J* = 8.1 Hz, 2H), 4.32 (d, *J* = 11.0 Hz, 2H), 4.20 (d, *J* = 11.1 Hz, 2H), 2.71–2.59 (m, 8H), 2.34 (q, *J* = 7.5 Hz, 4H), 1.20 (s, 3H), 1.13 (t, *J* = 7.5 Hz, 6H).

^13^C NMR (100 MHz, CDCl_3_) δ (ppm): 177.5, 172.9, 172.5, 170.4, 149.7, 149.2, 141.5, 110.2, 109.7, 65.9, 46.6, 31.9, 30.7, 29.3, 17.9, 9.4.

MS (HR-ESI^+^, *m*/*z*): calculated: 628.3; found: 629.3 [M + H]^+^, 651.2 [M + Na]^+^.

**Synthesis of Bn-d4DAP**. The compound was obtained according to the general esterification procedure described above from benzyl 2,2-di(hydroxymethyl)propanoate **(3)** and **HOOC-d2DAP**. The crude product was purified by flash column chromatography using EtOAc/DCM (7:3) as eluent and gradually increasing polarity to EtOAc. **Bn-d4DAP** was obtained as a white solid. Yield: 82%.

FTIR (ATR), ν (cm^‒1^): 3282, 1734, 1678, 1583, 1508, 1444, 1288, 1243, 1147.

^1^H NMR (400 MHz, CDCl_3_) δ (ppm) 8.38 (s, 4H), 8.27 (s, 4H), 7.88–7.78 (m, 8H), 7.61 (t, *J* = 8.1 Hz, 4H), 7.36–7.28 (m, 5H), 5.15 (s, 2H), 4.23–4.13 (m, 12H), 2.71–2.61 (m, 16H), 2.37 (q, *J* = 7.4 Hz, 8H), 1.23 (s, 3H), 1.18 (t, *J* = 7.5 Hz, 12H), 1.13 (s, 6H).

^13^C NMR (100 MHz, CDCl_3_) δ (ppm): 173.0, 172.4, 172.3, 172.2, 170.4, 149.9, 149.5, 140.9, 135.3, 128.9, 128.8, 128.5, 109.9, 109.5, 67.4, 65.5, 46.7, 46.5, 31.9, 30.7, 29.2, 17.9, 17.8, 9.5.

MS (HR-ESI^+^, *m*/*z*): calculated: 1444.6; found: 1445.6 [M + H]^+^, 1467.6 [M + Na]^+^.

**Synthesis of HOOC-d4DAP.** This compound was synthesized following the procedure described for **HOOC-d2DAP** using carbon-supported palladium catalyst (10%) (0.5 g) and **Bn-d4DAP** (4.85 g, 3.3 mmol) in EtOAc (70 mL). The crude product was purified by flash column chromatography on silica gel eluted using EtOAc to yield the target acid as a white solid. Yield: 85%.

FTIR (KBr), ν (cm^‒1^): 3546, 3316, 1734, 1690, 1586, 1521, 1447, 1290, 1240, 1149.

^1^H NMR (400 MHz, CDCl_3_) δ (ppm) 8.60 (s, 4H), 8.42 (s, 4H), 7.88–7.75 (m, 8H), 7.62 (t, *J* = 7.8 Hz, 4H), 4.25–4.18 (m, 12H), 2.70–2.62 (m, 16H), 2.39 (q, *J* = 7.6 Hz, 8H), 1.25–1.16 (m, 21H).

^13^C NMR (100 MHz, CDCl_3_) δ (ppm): 175.8, 173.1, 172.6, 170.6, 150.0, 149.5, 140.9, 110.1, 109.6, 66.7, 65.9, 46.6, 31.8, 30.6, 29.1, 17.9, 9.5.

MS (HR-ESI^+^, *m*/*z*): calculated: 1354.5; found: 1355.5 [M + H]^+^, 1377.5 [M + Na]^+^.

##### Synthesis of N_3_-dxDAP

**Synthesis of N_3_-d2DAP**. The compound was obtained following the described general amidation procedure from **N_3_-d2COOH** and 2-amino-6-propionylamidopyridine **(2)**. The crude product was purified by flash column chromatography on silica gel using EtOAc/hexane (6:4) as eluent and gradually increasing polarity to EtOAc/hexane (9:1). **N_3_-d2DAP** was isolated as a white solid. Yield: 56%.

FTIR (ATR), ν (cm^‒1^): 3292, 2096, 1733, 1676, 1583, 1508, 1444, 1288, 1242, 1147.

^1^H NMR (400 MHz, CDCl3) δ (ppm) 8.21 (s, 2H), 8.04 (s, 2H), 7.95–7.72 (m, 4H), 7.66 (t, *J* = 8 Hz, 2H), 4.33 (d, *J* = 11.1 Hz, 2H), 4.22 (d, *J* = 11.1 Hz, 2H), 4.09 (t, *J* = 6.6 Hz, 2H), 3.26 (t, *J* = 6.8 Hz, 2H), 2.78–2.51 (m, 8H), 2.39 (q, *J* = 8 Hz, 4H), 1.66–1.54 (m, 4H), 1.43–1.30 (m, 4H), 1.25–1.18 (m, 9H).

^13^C NMR (100 MHz, CDCl_3_) δ (ppm): 172.7, 172.6, 172.3, 170.0, 149.6, 149.3, 140.8, 109.6, 109.4, 65.2, 51.3, 46.3, 31.9, 30.6, 29.0, 28.7, 28.3, 26.3, 25.4, 18.1, 9.3.

MS (HR-ESI^+^, *m*/*z*): calculated: 753.8; found: 754.3 [M + H]^+^, 776.3 [M + Na]^+^.

**Synthesis of N_3_-d4DAP.** The compound was obtained according to the general esterification procedure described above, from 6-azidohexyl 2,2-di(hydroxymethyl)propanoate **(1)** and **HOOC-d2DAP**. The isolated crude product was purified by flash column chromatography using EtOAc/DCM (7:3) as eluent and gradually increasing polarity to EtOAc. The target compound was obtained as a white solid. Yield: 85%.

FTIR (ATR), ν (cm^‒1^): 3294, 2098, 1734, 1681, 1583, 1508, 1442, 1288, 1240, 1151.

^1^H NMR (400 MHz, CDCl_3_) δ (ppm) 8.37 (s, 4H), 8.27 (s, 4H), 7.89–7.78 (m, 8H), 7.62 (t, *J* = 8.1 Hz, 4H), 4.28–4.17 (m, 12H), 4.10 (t, *J* = 6.7 Hz, 2H), 3.26 (t, *J* = 6.8 Hz, 2H), 2.75–2.61 (m, 16H), 2.38 (q, *J* = 7.5 Hz, 8H), 1.67–1.55 (m, 4H), 1.41–1.33 (m, 4H), 1.21–1.17 (m, 21H).

^13^C NMR (100 MHz, CDCl_3_) δ (ppm): 172.9, 172.5, 172.4, 172.3, 170.3, 149.9, 149.5, 140.8, 109.9, 109.5, 65.6, 65.5, 51.4, 46.6, 46.5, 31.9, 30.7, 29.2, 28.8, 28.5, 26.5, 25.6, 18.0, 17.8, 9.5.

MS (MALDI^+^, dithranol, *m*/*z*): calculated: 1479.6; found: 1480.9 [M + H]^+^.

**Synthesis of N_3_-d8DAP**. This compound was synthesized following the general esterification procedure described above from compound **(4)** and **HOOC-d2DAP**. The crude product was purified by flash column chromatography with EtOAc as eluent and gradually increasing polarity to EtOAc/methanol (97:3) to yield the required compound as a white solid. Yield: 30%.

FTIR (ATR), ν (cm^‒1^): 3294, 2100, 1736, 1678, 1583, 1508, 1446, 1288, 1243, 1147.

^1^H NMR (400 MHz, CDCl_3_) δ (ppm) 8.48 (s, 8H), 8.37 (s, 8H), 7.81–7.70 (m, 16H), 7.53 (t, *J* = 8.1 Hz, 8H), 4.18–4.10 (m, 28H), 4.02 (t, *J* = 6.8 Hz, 2H), 3.19 (t, *J* = 6.8 Hz, 2H), 2.70–2.51 (m, 32H), 2.30 (q, *J* = 7.4 Hz, 16H), 1.59–1.49 (m, 4H), 1.32–1.28 (m, 4H), 1.18–1.08 (m, 45H).

^13^C NMR (100 MHz, CDCl_3_) δ (ppm): 173.1, 172.6, 172.3, 171.8, 170.5, 150.1, 149.6, 140.8, 110.0, 109.5, 66.2, 65.7, 65.5, 65.4, 51.4, 46.7, 46.6, 46.5, 31.8, 30.6, 29.8, 29.1, 28.8, 28.5, 26.4, 25.6, 17.9, 17.7, 17.6, 9.5.

MS (MALDI^+^, dithranol, *m*/*z*): calculated: 2933.2; found: 2933.6 [M]^+^.

**Synthesis of N_3_-d16DAP**. This compound was synthesized following the general esterification procedure from compound **(4)** and **HOOC-d4DAP**. The crude product was purified by flash column chromatography with EtOAc/methanol (99:1) eluent and gradually increasing polarity to EtOAc/methanol (9:1) to yield the requested compound as a white solid. Yield: 25%.

FTIR (ATR), ν (cm^‒1^): 3294, 2096, 1734, 1680, 1583, 1512, 1444, 1290, 1242, 1149.

^1^H NMR (400 MHz, CDCl_3_) δ (ppm) 8.63 (s, 16H), 8.52 (s, 16H), 7.86–7.75 (m, 32H), 7.58 (t, *J* = 8.0 Hz, 16H), 4.28–4.15 (m, 60H), 4.08 (t, *J* = 6.8 Hz, 2H), 3.25 (t, *J* = 6.8 Hz, 2H), 2.70–2.64 (m, 64H), 2.35 (q, *J* = 7.2 Hz, 32H), 1.62–1.53 (m, 4H), 1.37–1.33 (m, 4H), 1.25–1.14 (m, 93H).

^13^C NMR (100 MHz, CDCl_3_) δ (ppm): 173.3, 172.6, 172.3, 171.8, 171.7, 170.6, 150.1, 149.6, 140.8, 110.0, 109.5, 65.9, 65.5, 65.4, 51.4, 46.8, 46.7, 46.6, 31.7, 30.5, 29.8, 29.1, 28.8, 28.5, 26.4, 25.5, 17.9, 17.7, 17.5, 9.5.

MS (MALDI^+^, dithranol, *m*/*z*): calculated: 5839.3; found: 5830.2 [M]^+^, 5855.7 [M + Na]^+^.

#### 2.1.4. Synthesis of PEG_2k_-Alky

Polyethylenglycol monomethyl ether (**PEG_2k_-OH**) (4.33 g, 2.2 mmol), 4-pentyonic acid (0.29 g, 2.9 mmol) and DPTS (0.38 g, 1.3 mmol) were dissolved in dry DCM (25 mL) under Ar atmosphere. After the solution was cooled to 0 °C, DCC (0.59 g, 2.9 mmol) was added. The solution was stirred for 1 h at 0 °C and then at room temperature for 48 h. The formed solid was filtered off and the solvent was removed under vacuum. The crude product was precipitated from a large volume of cold diethyl ether. Then, the solid was recovered by filtration and dried under vacuum. The target compound was obtained as a white powder. Yield: 85%.

FTIR (ATR), ν (cm^‒1^): 2164, 1736, 1097.

^1^H NMR (400 MHz, CDCl_3_) δ (ppm): 4.25(t, *J* = 4.6 Hz, 2H), 3.90–3.42 (m, 180 H), 3.37 (s, 3H), 2.61–2.55 (m, 2H), 2.53–2.47 (m, 2H), 1.98 (t, *J* = 2.6 Hz, 1H).

MS (MALDI^+^, dithranol, *m*/*z*): found: 1983.9 [M]^+^ (Corresponding to the most intense *m*/*z*peak).

#### 2.1.5. General Synthetic Procedure for the Synthesis of the LDBCs PEG_2k_-b-dxDAP

The dendron block **N_3_-dxDAP** (1.0 mol) and the linear alkyne-functionalized PEG (**PEG_2k_-Alky**) (1.2 mol) were placed into a Schlenk tube with distilled *N,N*-dimethylformamide (DMF) (15 mL). The flask was degassed by three freeze–pump–thaw cycles and flushed with Ar, and then maintained under Ar atmosphere at 45 °C. A second Schlenk tube, containing CuSO_4_·5H_2_O (0.1 mol), l-ascorbate (0.2 mol), and TBTA (0.1 mol) in distilled DMF (5 mL), was also degassed by three freeze–pump–thaw cycles and flushed with Ar, and then maintained under Ar atmosphere at 45 °C until the color solution changed from blue to yellow. Then, the content was transferred using a cannula to the initial solution of the azide (**N_3_-dxDAP**) and alkyne (**PEG_2k_-Alky**) compounds. The reaction was stirred at 45 °C and monitored by thin layer chromatography (TLC). Once the reaction was completed, an excess of **PEG_2k_-Alky** was removed by adding azide-functionalized Merrifield’s resin, and the reaction mixture was additionally stirred for 24 h. Merrifield’s resin was filtered off, and the collected solution was diluted with DCM washed with a KCN aqueous solution (15 mg of KCN/100 mL of water) to remove copper and then with brine, dried over anhydrous MgSO_4_, and concentrated under vacuum. The resulting crude product was precipitated from a large volume of cold diethyl ether.

**Synthesis of PEG_2k_-b-d2DAP.** The compound was obtained according to the general synthetic procedure described above, from **N_3_-d2DAP**. The product was obtained as a white powder. Yield: 35%.

FTIR (ATR), ν (cm^‒1^): 3288, 1736, 1695, 1585, 1516, 1446, 1101.

^1^H NMR (400 MHz, CDCl_3_) δ (ppm): 8.30 (s, 2H), 8.08 (s, 2H), 7.97–7.72 (m, 4H), 7.66 (t, *J* = 8.0 Hz, 2H), 7.39 (s, 1H), 4.42–4.15 (m, 8H), 4.07 (t, *J* = 6.4 Hz, 2H), 3.88–3.42 (m, 183H) 3.37 (s, 3H), 3.04 (t, *J* = 7.0 Hz, 2H), 2.84–2.54 (m, 10H), 2.40 (q, *J* = 7.4 Hz, 4H), 1.89–1.81 (m, 2H), 1.67–1.51 (m, 2H), 1.41–1.12 (m, 13H).

MS (MALDI^+^, dithranol, *m*/*z*): calculated: 2737.2; found: 2737.9 [M]^+^.

**Synthesis of PEG_2k_-b-d4DAP.** The compound was obtained according to the general synthetic procedure described above, from **N_3_-d4DAP**. The product was obtained as a white powder. Yield: 71%.

FTIR (ATR), ν (cm^‒1^): 3284, 1736, 1695, 1585, 1518, 1446, 1101.

^1^H NMR (400 MHz, CDCl_3_) δ (ppm) 8.50 (s, 4H), 8.32 (s, 4H), 7.97–7.71 (m, 8H), 7.62 (t, *J* = 8.1 Hz, 4H), 7.37 (s, 1H), 4.32–4.15 (m, 16H), 4.07 (t, *J* = 6.6 Hz, 2H), 3.73–3.57 (m, 190H), 3.37 (s, 3H), 3.05 (t, *J* = 7.3 Hz, 2H), 2.82–2.56 (m, 18H), 2.39 (q, *J* = 7.5 Hz, 8H), 1.90–1.81 (m, 2H), 1.66–1.56 (m, 2H), 1.38–1.26 (m, 4H), 1.23–1.13 (m, 21H).

MS (MALDI^+^, dithranol, *m*/*z*): calculated: 3463.8; found: 3509.8 [M]^+^.

**Synthesis of PEG_2k_-b-d8DAP.** The compound was obtained according to the general synthetic procedure described above, from **N_3_-d8DAP**. The product was obtained as a colorless viscous oil. Yield: 45%.

FTIR (ATR), ν (cm^‒1^): 3275, 1736, 1691, 1585, 1519, 1444, 1112.

^1^H NMR (400 MHz, CDCl_3_) δ (ppm) 8.62 (s, 8H), 8.47 (s, 8H), 7.93–7.73 (m, 16H), 7.59 (t, *J* = 8.1 Hz, 8H), 7.37 (s, 1H), 4.28–4.15 (m, 32H), 4.06 (t, *J* = 6.6 Hz, 2H), 3.70–3.55 (m, 196H), 3.37 (s, 3H), 3.04 (t, *J* = 7.2 Hz, 2H), 2.80–2.58 (m, 34H), 2.36 (q, *J* = 7.4 Hz, 16H), 1.87–1.82 (m, 2H), 1.62–1.56 (m, 2H), 1.34–1.29 (m, 4H), 1.23–1.14 (m, 45H).

MS (MALDI^+^, dithranol, *m*/*z*): calculated: 4917.5; found: 4919.8 [M]^+^.

**Synthesis of PEG_2k_-*b*-d16DAP.** The compound was obtained according to the general synthetic procedure described above, from **N_3_-d16DAP**. The product was obtained as a colorless viscous oil. Yield: 39%.

FTIR (ATR), ν (cm^‒1^): 3298, 1736, 1691, 1585, 1516, 1444, 1149.

^1^H NMR (400 MHz, CDCl_3_) δ (ppm) 8.70 (s, 16H), 8.56 (s, 16H), 7.92–7.66 (m, 32H), 7.57 (t, *J* = 8.0 Hz, 16H), 7.37 (s, 1H), 4.29–4.08 (m, 66H), 3.65–3.56 (m, 180H), 3.37 (s, 3H), 3.04 (t, *J* = 7.1 Hz, 2H), 2.78–2.64 (m, 66H), 2.35 (q, *J* = 7.2 Hz, 32H), 1.89–1.81 (m, 2H), 1.62–1.56 (m, 2H), 1.31–1.13 (m, 100H).

MS (MALDI^+^, dithranol, *m*/*z*): calculated: 7814.1; found: 7720.9 [M]^+^.

#### 2.1.6. Synthesis of the Thymine Based Cross-Linkers

**Synthesis of thym-C_12_-thym**. A suspension of 1,12-dibromodecane (2.59 g, 7.90 mmol), thymine (4.85 g, 38.44 mmol), and anhydrous Na_2_CO_3_ (5.72g, 53.97mmol) in DMF (120 mL) was stirred for 40 h at 80 °C, while the reaction progress was monitored by TLC. The solid was filtered off and thoroughly washed with EtOAc. The combined filtrates were diluted with EtOAc (300 mL) and washed with water (3 × 75 mL) and brine (3 ×75 mL), dried over anhydrous MgSO_4_, and evaporated. A mixture of 1- and 3-substituted thymines was obtained that was purified firstly by flash column chromatography on silica gel using DCM as eluent, gradually increasing the polarity to DCM/methanol (9:1), and then by flash column chromatography on neutral alumina with DCM as eluent, gradually increasing the polarity to DCM/methanol (92:8). Yield: 28%

FTIR (ATR), ν (cm^‒1^): 3155, 3033, 2931, 2858, 1687, 1651, 1479.

^1^H NMR (400 MHz, CDCl_3_) δ (ppm): 9.54 (s, 2H), 6.97 (d, *J* = 1.2 Hz, 2H), 3.71–3.64 (m, 4H), 1.91 (d, *J* = 1.0 Hz, 6 H), 1.70–1.59 (m, 4H), 1.26 (d, *J* = 21.0 Hz, 16H).

^13^C NMR (100 MHz, CDCl_3_) δ (ppm): 164.5, 151.1, 140.5, 110.7, 48.6, 29.5, 29.2, 26.5, 12.5.

MS (HR-ESI^+^, *m*/*z*): calculated: 418.26; found: 419.27 [M + H]^+^, 441.25 [M + Na]^+^.

**Synthesis of (thym-C_6_)_4_-O.** 1-(3-Dimethylaminopropyl)-3-ethylcarbodiimide hydrochloride (EDC) (2.34 g, 12.29 mmol) was added to a solution of 6-(5-methyl-2,4-dioxo-3,4-dihydro-2H-pyrimidin-1-yl)hexanoic acid **(5)** (2.81 g, 11.69 mmol), pentaerythritol (0.29 g, 2.12 mmol), and DPTS (0.90 g, 3.06 mmol) in dry DCM (25 mL) under Ar atmosphere and cooled in a salt–ice bath. After 30 min, the salt–ice bath was removed, and the reaction was stirred for additional 96 h while progress was followed by TLC. The reaction was diluted with DCM (150 mL), and the resulting organic solution was washed with distilled water (3 × 40 mL) and brine (2x40 mL), dried over anhydrous MgSO_4_, and evaporated. The obtained residue was purified by flash column chromatography on silica gel using DCM as eluent and gradually increasing polarity to DCM/methanol (9:1). Yield: 83%

FTIR (ATR), ν (cm^‒1^): 3500, 3184, 3061, 2955, 2868, 1716, 1666, 1471, 1363, 1250, 1221.

^1^H NMR (400 MHz, CDCl_3_) δ (ppm): 9.61 (s, 4H), 7.02 (d, *J* = 1.2Hz, 4H), 4.10 (s, 8H), 3.70 (t, *J* = 7.2Hz, 8H), 2.33 (t, *J* = 7.3Hz, 8H), 1.91 (d, *J* = 1.1 Hz, 12H), 1.66 (m, 16H), 1.40–1.28 (m, 8H).

^13^C NMR (100 MHz, CDCl_3_) δ (ppm): 173.00, 164.63, 151.25, 140.56, 110.84, 62.26, 48.20, 42.14, 33.82, 28.83, 25.83, 24.36, 12.47.

MS (HR-ESI^+^, *m*/*z*): calculated: 1024.47; found: 1047.47 [M + Na]^+^.

### 2.2. Preparation of Self-Assemblies in Water

#### 2.2.1. Preparation of Self-Assemblies by Nanoprecipitation

Milli-Q^®^ water was gradually added to a solution of 5 mg mL^−1^ of the corresponding LDBC, **PEG_2k_-*b*-dxDAP**, in THF (HPLC grade). The self-assembly process was followed by turbidimetry, recording the decrease of transmitted light intensity at 650 nm due to aggregation and the subsequent scattering upon water addition. When a constant value was reached, the suspension was dialyzed against a large volume of Milli-Q^®^ water to remove THF, using a Spectra/Por^®^ dialysis membrane (MWCO 1 kDa) (Repligen Europe B.V., Breda, The Netherlands) for 4 days (water was periodically refreshed).

#### 2.2.2. Preparation of Self-Assemblies by Microfluidic Technology

A solution of 5 mg mL^−1^ of **PEG_2k_-*b*-dxDAP** in THF (HPLC grade) was filtered through a 0.2 μm PTFE filter. This organic solution and Milli-Q^®^ water were fed into a passive micromixer using two syringe pumps Harvard Apparatus PHD Ultra CP 4400 (Harvard Apparatus, Holliston, MA, USA), one for each solution. The micromixer employed was a commercial slit interdigital microstructured mixer from IMM (Institut für Mikrotechnik Mainz GmbH, Germany) interfaced with 0.76 mm internal diameter PTFE tubing. This micromixer, which has a volume of 8 µL, was designed for dividing the inlet streams into 15 channels of 40 µm that were merged at the outlet, achieving instant mixing [51]. Injection flow rates of each solution were adjusted to collect suspensions with different aqueous/organic solution ratios at a total flow value of 10 mL min^−1^ and a mixing time of just 48 milliseconds. Samples were collected after waiting several seconds to reach the micromixer’s steady state. Then, they were dialyzed against Milli-Q^®^ water, to remove THF, using a Spectra/Por^®^ dialysis membrane (MWCO 1 kDa) for 4 days (water was periodically refreshed). The synthesis of these nanomaterials has been performed by the Synthesis of Nanoparticles Unit of the ICTS “NANBIOSIS”.

#### 2.2.3. Determination of the Critical Aggregation Concentration (CAC)

Critical aggregation concentration (CAC) was determined by fluorescence spectroscopy employing Nile Red as probe. First, 100 µL of a Nile Red solution in DCM (6.0 × 10^−6^ M) was added into a series of flasks, and then, the solvent was evaporated. Afterwards, water suspensions of self-assemblies were added to each flask with concentrations ranging from 1.0 × 10^−4^ to 1.0 mg mL^−1^. The resulting solutions were stirred overnight at room temperature to reach equilibrium before fluorescence was measured. The emission spectra of Nile Red were recorded from 560 to 700 nm while exciting at 550 nm, and maximum emission at different concentrations was represented. CAC was calculated as the intersection of the tangents of the Nile Red emission increase with the extrapolated baseline (onset value).

#### 2.2.4. Preparation of Rhodamine B-Loaded Vesicles

Rhodamine B-loaded vesicles were prepared by nanoprecipitation by adding to the LDBCs solution in THF (5 mg mL^−1^) a solution of Rhodamine B in Milli-Q^®^ water. The amount of Rhodamine B was adjusted to add an average of 5 molecules of Rhodamine B per molecule of polymer. Once vesicles were formed, the suspension was dialyzed against water to remove non-encapsulated Rhodamine B.

### 2.3. Characterization Techniques and Instrumentation

Fourier Transformed Infrared Spectroscopy (FTIR) was applied using a Bruker Vertex 70 spectrophotometer (Bruker, Ettlingen, Germany) having an ATR Golden Gate accessory™ (Specac Ltd., Orpington, Kent, UK). Solution NMR experiments were carried out on a Bruker AV-400 spectrometer (Bruker, Billerica, MA, USA) operating at 400 MHz for ^1^H and 100 MHz for ^13^C, using standard pulse sequences. Chemical shifts are given in ppm relative to TMS, and the solvent residual peak was used as an internal reference. MALDI-TOF MS was performed on an Autoflex mass spectrometer (Bruker, Billerica, MA, USA). Gel Permeation Chromatography (GPC) was performed using a Water Alliance 2695 liquid chromatography system with a Waters 2424 evaporation light scattering detector and a Waters 2998 PDA detector (Waters, Milford, MA, USA), using two PLgel 5µm MIXED-C Agilent columns (7.5 × 300 mm) and THF (HPLC grade) as eluent (flow 1 mL min^−1^). Calibration was made with polymethacrylate (PMMA) narrow molecular weight standards.

Thermogravimetric analysis (TGA) was performed using a Q5000 module from TA Instruments (TA Instruments, New Castle, DE, USA) at a heating rate of 10 °C min^−1^ under nitrogen atmosphere. Decomposition temperature (T_onset_) was given as the onset of the peak recorded at the first derivative of the mass loss. Differential Scanning Calorimetry (DSC) was carried out to determine thermal transitions using a Q2000 calorimeter from TA Instruments on powdered samples (2–5 mg) sealed in aluminium pans. Glass transition temperatures (T_g_) were determined at the half-height of the baseline jump, and melting temperatures (T_m_) were read at the maximum of the corresponding peaks.

Ultraviolet-visible (UV-vis) spectra were recorded on a Varian CaryBio 100 UV-vis spectrophotometer (Agilent, Santa Clara, CA, USA). Fluorescence measurements were performed using a Perkin Elmer LS 50B fluorescence spectrophotometer (PE Corporation, Waltham, MA, USA).

Dynamic Light Scattering (DLS) measurements were carried out in a Malvern Instrument Nano Zs (Malvern, Worcestershire, UK) using a He-Ne laser with a 633 nm wavelength and a detector angle of 173° at 25 °C. The self-assemblies were measured at approximately 0.1 mg mL^−1^ concentration, and hydrodynamic diameters (D_h_) were given as an average of three measurements on each sample to ensure reproducibility. Transmission Electron Microscopy (TEM) and Cryogenic Transmission Electron Microscopy (cryo-TEM) analysis were developed in a FEI Tecnai T20 microscope (FEI Company, Waltham, MA, USA), operating at 200 kV. In the case of TEM, samples were prepared placing a droplet of self-assemblies’ dispersion on a continuous carbon film-copper grid, and the excess was removed by capillarity using filter paper. Then, samples were stained with uranyl acetate (1% aqueous solution), removing the excess again by capillarity using filter paper. The grids were dried overnight under vacuum. For cryo-TEM, Holey carbon film-copper grids previously ionized using a plasma cleaner were employed. A droplet was placed onto the grid, and sample vitrification was automatically processed using FEI Vitrobot (FEI Company, Waltham, MA, USA) and performed in liquid ethane. Samples were maintained under liquid nitrogen with a Gatan TEM cryo-holder (FEI Company) and observed operating at 80 kV. Fluorescence vesicles were observed with an Olympus FV10i confocal scanning microscope (Olympus, Tokyo, Japan). Samples were prepared placing a droplet of self-assemblies’ dispersion onto an Ibidi^®^ µ-dish 35 mm (Ibidi Inc., Gräfelfing, Germany), and a cover slip was placed on top of the sample.

### 2.4. Cell Culture

The cytocompatibility of the self-assemblies was assessed at different levels regarding cell metabolism, cell membrane (induction of apoptosis and/or necrosis), and cell nucleus (distribution of cell cycle phases). These studies were performed by using THP1 human macrophages (obtained from the American Type Culture Collection, US), human dermal fibroblasts (purchased from Lonza, Belgium), and mouse mesenchymal stem cells (mMSCs) and human glioblastoma cells (U251MG) (both kindly gifted by Dr. Pilar Martín-Duque). Macrophages were obtained by the in vitro differentiation of monocytes through the addition of 1 µM phorbol 12-mystirate 13-acetate (Sigma Aldrich, St. Louis, MO, USA) to the cell culture medium. Fibroblasts and U251MG cells were grown in DMEM high glucose (DMEM w/stable Glutamine; Biowest, France) supplemented with 10% fetal bovine serum (Gibco, UK) and 1% penicillin–streptomycin–amphotericin B (Biowest, France). mMSCs were cultured in DMEM-F12 (Biowest, France) containing 1% glutamine (Biowest, France), 10% fetal bovine serum (Gibco, UK), and 1% penicillin– streptomycin–amphotericin B (Biowest, France). All cell types were grown at 37 °C and 5% CO_2_, except for mMSCs which were cultured in hypoxia (3% O_2_).

### 2.5. Cytotoxicity Assays

The effect of self-assemblies in cell metabolism was determined by the Blue Cell Viability assay (Abnova, Taiwan), which is based on the reduction of a dye to a fluorescent compound mediated by metabolically active cells. For that purpose, cells were incubated with self-assemblies (0.01–0.4 mg mL^−1^) for 24 h. Then, the reagent was added (10%) and incubated for 4 h. After that, fluorescence was read at 535/590 nm (ex/em) in a Multi-mode Synergy HT Microplate Reader (Biotek, US). Cell viability was determined by the interpolation of the emission data obtained from the treated samples and the control ones, which were assigned with 100% viability.

### 2.6. Evaluation of Early and Late Cell Apoptosis and/or Necrosis

The effect of self-assemblies on the cell membrane was determined by a quantitative analysis of cell apoptosis and necrosis by flow cytometry. Cells were incubated with the different samples at the subcytotoxic concentration obtained from the Blue Cell Viability assay for 24 h. Then, cells were harvested in PBS and double stained with annexin V-FITC and propidium iodide. Briefly, cell suspensions were stained with annexin V-FITC, treated with a solution composed of annexin V-FITC, propidium iodide, and annexin V binding buffer, to be finally incubated with the binding buffer for 15 min before the analysis of the samples in a FACSARIA BD equipment, using the FACSARIA BD software (Cell Separation and Cytometry Unit, CIBA, Spain) to calculate the percentages of alive, apoptotic, and necrotic cells. Control samples were also evaluated as reference. 

### 2.7. Study of Cell Cycle

The effect of self-assemblies on cell cycle and DNA was determined by analyzing the distribution of cell cycle phases by flow cytometry. As described above, cells were incubated with the different samples at the subcytotoxic concentration for 24 h and then, they were collected in PBS and fixed with 70% ice-cold ethanol. After 24 h of incubation at 4 °C, DNA staining was performed by adding RNAase A and propidium iodide. Samples were analyzed in a FACSARRAY BD equipment with the MODIFIT 3.0 Verity software (Cell Separation and Cytometry Unit, CIBA, Spain), and control samples were also evaluated.

## 3. Results and Discussion

### 3.1. Synthesis and Characterization of the Amphiphilic Linear–Dendritic Block Copolymers

Amphiphilic LDBCs were denoted as **PEG_2k_-*b*-dxDAP** where 2k represents the approximate average molar mass of the PEG block (according to MALDI-TOF MS) (Figure S1) and x accounts for the number of peripheral groups (x = 2, 4, 8, or 16) at the dendritic block. The LDBCs were obtained by click coupling of the blocks using the copper(I)-catalyzed azide-alkyne cycloaddition (CuAAC) reaction (Scheme 1). Therefore, the hydrophilic poly(ethylene glycol) monomethyl ether block with a terminal alkyne group (PEG_2k_-Alky) was coupled to the first four generations of hydrophobic dendrons derived from bis-MPA monomer, which were functionalized with 2,6-diaminopyridine units at the periphery and having a 6-azidohexyl group at the focal point (**N_3_-dxDAP**).

The synthesis of the first-generation dendron **N_3_-d2DAP** is outlined in Scheme 2. The dendron incorporates the DAP units into 6-azidohexyl 2,2-di(hydroxymethyl)propanoate **(1)** through a succinic connector. The syntheses of the second (**N_3_-d4DAP**), third (**N_3_-d8DAP**), and fourth (**N_3_-d16DAP**) generation dendrons are shown in Scheme 3 according to a double-exponential dendrimeric growth [52]. In general, dendrons were prepared by esterification reaction of the peripheral hydroxyl groups of a bis-MPA wedge having and an azido focal group with the carboxylic focal group of a bis-MPA wedge with peripheral DAP units. Thus, the reaction of bis-MPA decorated with two DAP units (**HOOC-d2DAP**), with either compound **(1)** or compound **(4)**, yielded **N_3_-d4DAP**, or **N_3_-d8DAP**, respectively. Likewise, the reaction of **HOOC-d4DAP**, which was obtained from **HOOC-d2DAP** and benzyl 2,2-di(hydroxymethyl)propanoate **(3)**, with compound **(4)** afforded **N_3_-d16DAP**. All the compounds were characterized by ^1^H-NMR, ^13^C-NMR, and FTIR.

The coupling of both blocks, **PEG_2k_-Alky** and **N_3_-dxDAP**, was accomplished by CuAAC in DMF using CuSO_4_ as catalyst, l-ascorbate as reducing agent, and TBTA as stabilizing ligand, and a slight excess of the PEG block that was easily removed using an azide-functionalized Merryfield resin. The complete coupling with absence of non-coupled segments was assessed by MALDI-TOF MS and GPC (Figures S36–S40). Details of calculated molar masses, dispersity, and hydrophobic/hydrophilic ratios are given in Table 1.

Thermal stability of **PEG_2k_-*b*-dxDAP**, as well as of **N_3_-dxDAP** precursors, was determined by TGA using powdered samples. All the samples exhibited a good thermal stability with the onset of decomposition associated to mass loss at approximately 240–260 °C (Table 2). **PEG_2k_-*b*-dxDAP** thermal stability was slightly higher than that of the corresponding **N_3_-dxDAP**.

Thermal transitions were evaluated by DSC between ‒50 and 150 °C, and the relevant parameters are gathered in Table 2. Neat **PEG_2k_-OH** has been described as a semicrystalline polymer with a melting temperature close to 50 °C [53]. Dendrons **N_3_-dxDAP** were of amorphous nature with only a glass transition (Tg) on DSC scans whose value, calculated from a second heating scan, increased with the dendron generation from 19 °C (for **N_3_-d2DAP**) to 73 °C (for **N_3_-d16DAP**) (Figure S41). **PEG_2k_-*b*-dxDAP** LDBCs were obtained as semicrystalline materials having endothermic transitions at temperatures of about 40 °C, likely corresponding to melting of the PEG block. No other transitions associated, such as the Tg of the dendronized blocks, were observed on the corresponding first heating scans. On cooling, the tendency of the melted LDBCs to crystallize decreased upon increasing the dendron generation. **PEG_2k_-*b*-d2DAP** and **PEG_2k_-*b*-d4DAP** underwent a cold crystallization process and subsequent melting of the crystalline fraction at approximately 40 °C as the only discernible thermal transitions on heating. However, for **PEG_2k_-*b*-d8DAP** and **PEG_2k_-*b*-d16DAP**, crystallization was largely suppressed, and only a glass transition was clearly observed at about −10 °C for both LDBCs, which might indicate at least partial miscibility between both polymeric blocks. Partial miscibility has also been observed for linear BC analogues of PEG and a polymethacrylate with DAP side units [40] (Figure S42).

### 3.2. Self-Assembly of the Linear–Dendritic Amphiphilic BCs in Water by Nanoprecipitation

**Figure 1:**
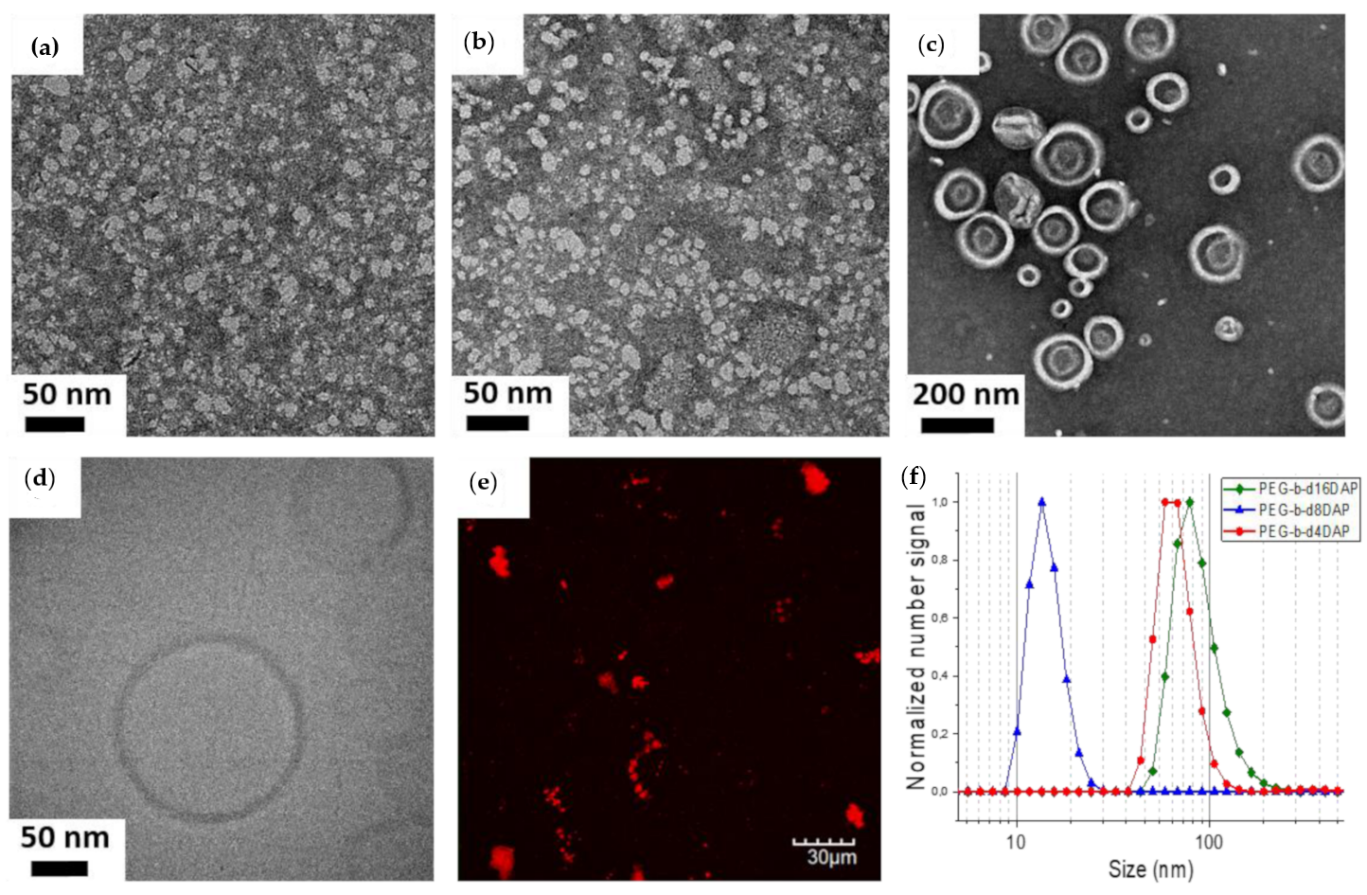
TEM images of (**a**) **PEG_2k_-*b*-d4DAP**, (**b**) **PEG_2k_-*b*-d8DAP**, and (**c**) **PEG_2k_-*b*-d16DAP** self-assemblies prepared by nanoprecipitation. (**d**) Cryogenic Transmission Electron Microscopy (Cryo-TEM) image of **PEG_2k_-*b*-d16DAP** vesicles. (**e**) Fluorescence microscopy image of the water suspension of **PEG_2k_-*b*-d16DAP** vesicles with encapsulated Rhodamine B. (**f**) Number particle size distributions recorded by Dynamic Light Scattering (DLS) of LDBCs self-assemblies prepared by nanoprecipitation.

Polymeric self-assemblies’ suspensions in water were prepared by gradually adding water into THF solutions of the LDBCs while monitoring changes on the light transmittance, as these changes are associated to the formation of the self-assemblies (Figure S43) [54]. Once the turbidity reached a stable value, the resulting dispersion was dialyzed against water to remove the organic solvent. The morphological characteristics of the self-assemblies were evaluated by TEM while their size, associated to the hydrodynamic volume (D_h_), was determined by DLS (Figure 1 and Figure S44).

Inspection of **PEG_2k_-*b*-d2DAP** dispersions revealed that this LDBC did not form stable self-assemblies and, for this reason, it was excluded from any further study. **PEG_2k_-*b*-d4DAP** showed self-assembled structures without a clearly defined morphology (Figure 1a). The mean average hydrodynamic diameter (D_h_) of 63 ± 25 nm as determined by DLS and a small population having larger sizes was also observable when representing the size distribution by intensity (Figure S44). The presence of this second size distribution and the large differences between the D_h_ and sizes inferred from the TEM images might be related to micellar aggregation. For **PEG_2k_-*b*-d8DAP**, better defined spherical micelles were observable in TEM images (Figure 1b) with D_h_ values of 14 ± 5 nm. Even though in this case, D_h_ was consistent with the TEM images, a small population having larger sizes was again observable by DLS when representing the size distribution by intensity that could also stem from some extent of micellar aggregation (Figure S44).

According to TEM images, **PEG_2k_-*b*-d16DAP** formed self-assemblies with very dissimilar sizes and an apparent vesical morphology (Figure 1c). In order to assess the morphology, the **PEG_2k_-*b*-d16DAP** self-assemblies dispersion was also inspected by cryo-TEM (Figure 1d). Spherical unilamellar vesicles with diameters in the range 50–245 nm and a membrane thickness of 8.9 ± 1.1 nm were observed. This thickness is comparable to other reported vesicles and fits well with a bilayer arrangement of the hydrophobic dendrons at the inner part of the vesicle membrane [23,25]. To further reassert the formation of **PEG_2k_-*b*-d16DAP** vesicles, loading experiments were performed using the fluorescent probe Rhodamine B because, as being a hydrophilic molecule, it should be entrapped inside the internal aqueous lumen of these vesicles. **PEG_2k_-*b*-d16DAP** self-assemblies formed in the presence of Rhodamine B were examined by confocal fluorescence microscopy where fluorescence dots in a dark background were observed that correspond to clusters of vesicles loaded with the probe (Figure 1e). For **PEG_2k_-*b*-d16DAP**, apparent D_h_ determined by DLS was 79 ± 36 nm.

The CAC of **PEG_2k_-*b*-dxDAP** was determined by fluorescence spectroscopy using Nile Red as a polarity sensitive probe (Figure S45) [25]. CAC values were 117, 41, and 25 µg mL^−1^, for **PEG_2k_-*b*-d4DAP**, **PEG_2k_-*b*-d8DAP**, and **PEG_2k_-*b*-d16DAP** respectively, which are in accordance with reported values for amphiphilic linear and linear–dendritic BCs of similar hydrophobic/hydrophilic ratios [25,55,56,57]. The thermodynamic stability of the self-assembly usually correlates with CAC, being higher as the CAC lowers. In this case, CAC decreases upon increasing the dendron generation, i.e., on increasing the hydrophobic content of the LDBC. In particular, the significant differences between **PEG_2k_-*b*-d4DAP** (117 µg mL^−1^) and **PEG_2k_-*b*-d8DAP** (41 µg mL^−1^), both of them apparently forming micelles, agrees with the greater facility of **PEG_2k_-*b*-d8DAP** to self-assemble in water inferred by TEM and DLS.

### 3.3. Self-Assembly of the Linear–Dendritic Amphiphilic BCs in Water by Microfluidics

Once we verified the self-assembly ability of these LDBCs, the preparation of polymeric nanoparticles was accomplished by microfluidics as a very reproducible, fast, and scalable methodology for preparing polymeric self-assemblies in a continuous manner. Due to their small dimensions, microreactors provide better control in the preparation of nanoparticles because mass and heat transport processes are fast and predictable, and heating and mixing are also uniform and controlled. Other additional advantages of this technology are the high reproducibility that allows an easy scale-up by increasing the number of microreactors, and the possibility of tuning the size and the shape of nanoparticles, due to a better control of the experimental parameters [58,59,60].

For that purpose, a solution of **PEG_2k_-*b*-dxDAP** (with x = 4, 8, and 16) in THF was instantaneously mixed with Milli-Q^®^ water, using a commercial slit interdigital microstructured mixer. Both solutions were fed in continuous and non-pulsed flow into the micromixer using two syringe pumps. The total flow rate and phase ratio values were modified to optimize the process. Consequently, dispersions with different aqueous/organic solution phase ratios (Table 3) were collected at a total flow rate finally fixed at 10 mL min^−1^. All the samples were dialyzed against water after being collected in order to remove THF.

**Figure 2:**
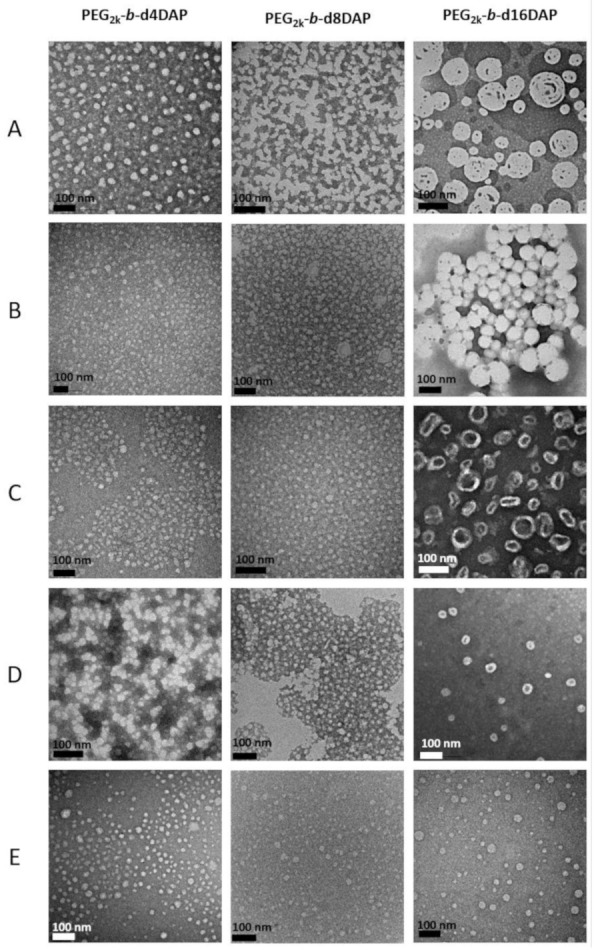
TEM images of **PEG_2k_-*b*-dxDAP** self-assemblies prepared by microfluidics at different experimental conditions (aqueous/organic phase ratio) according to Table 3: (**A**) (60/40); (**B**) (70/30); (**C**) (80/20); (**D**) (90/10); and (**E**) (95/5).

**Figure 3:**
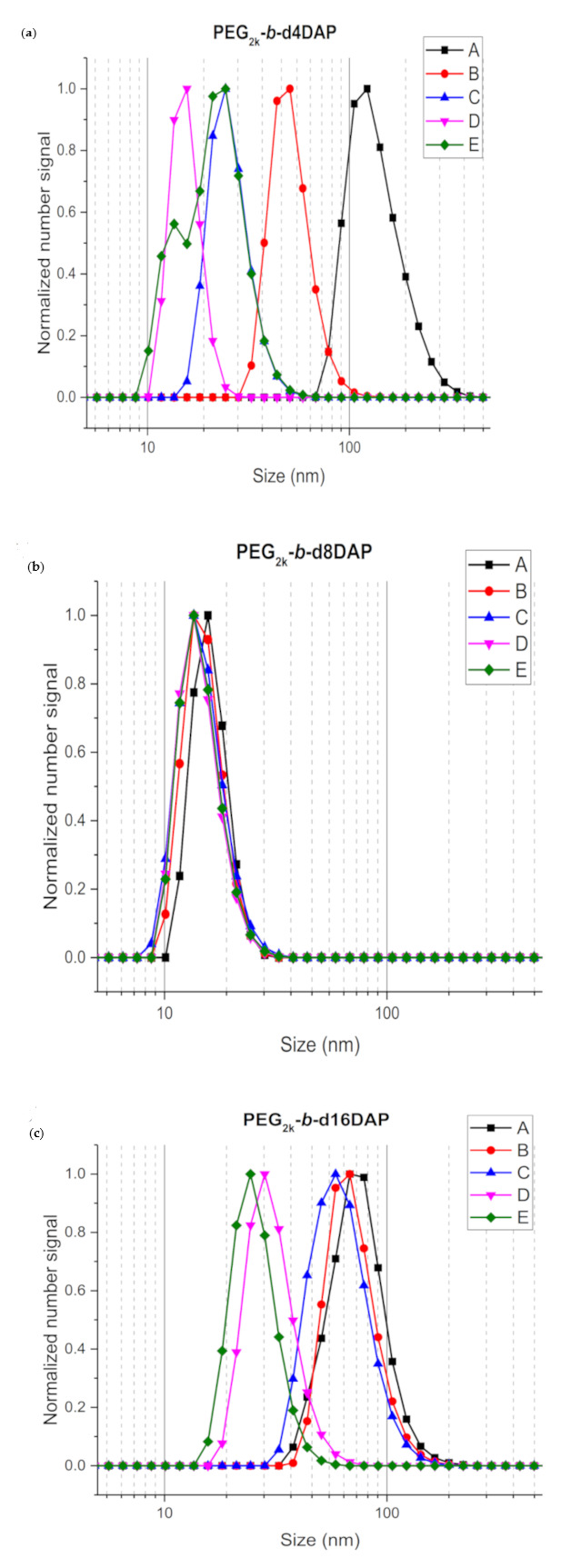
Number size distributions recorded by DLS of **PEG_2k_-*b*-dxDAP** self-assemblies prepared by microfluidics at different experimental conditions (aqueous/organic phase ratio) according to Table 3: A (60/40), B (70/30), C (80/20), D (90/10), and E (95/5): (**a**) **PEG_2k_-*b*-d4DAP**, (**b**) **PEG_2k_-*b*-d8DAP** and (**c**) **PEG_2k_-*b*-d16DAP.**

In the tested conditions, **PEG_2k_-*b*-d4DAP** self-assembled into spherical micelles with diameters ranging from 15 to 30 nm, according TEM images (Figure 2). As can be seen in Figure 3, in general, DLS measurements showed a substantial effect of microfludics experimental phase ratios on D_h_ with the size of micelles increasing when decreasing the water phase ratio, except for the sample obtained using a higher water phase ratio (**E**), which exhibited two size distributions in number recorded by DLS. In addition, in terms of size, a disparity was generally observed between TEM and DLS mainly for low water phase ratios (**A-B**). These irregularities might presumably be a consequence of some micellar aggregation associated to a lower stability of **PEG_2k_-*b*-d4DAP** micelles, as hypothesized for the corresponding self-assemblies prepared by nanoprecipitation.

**PEG_2k_-*b*-d8DAP** self-assembled forming dispersions of uniform spherical micelles in all conditions. Average D_h_ values were about 15 ± 5 nm, which was in good concordance with TEM, slightly increasing on increasing the water phase ratio (Figure 3). For **PEG_2k_-*b*-d16DAP**, a transition from vesicles to micelles was observed on increasing the water ratio (Figure 2). Using 60–80% water (**A-C** conditions), **PEG_2k_-*b*-d16DAP** self-assembled into vesicles of polydisperse sizes, as observed in TEM images (Figure 2), with average D_h_ values decreasing from 80 to 50 (± 35) nm upon increasing the water content (Figure 3). However, spherical micelles were formed at higher water contents (90–95%) having D_h_ values of 30 ± 13 and 24 ± 9 nm for **D** and **E** conditions, respectively. For **D** conditions, micelles coexisted with small vesicles, and some micellar aggregation was also detectable. For **E** conditions, only well-defined spherical micelles were formed with sizes matching well with DLS measurements. Thus, when using microfluidics, tuning the water phase ratio enables control over very distinct morphologies.

Therefore, the use of microfluidics allows a good control over the mixing conditions, a high nanocarrier production rate (ranging from 550 to 725 mg h^−1^), and assures homogenous self-assembly in water for **PEG_2k_-*b*-d8DAP** or control over the morphology for **PEG_2k_-*b*-d16DAP.**

### 3.4. Cell Viability, Apoptosis, and Cell Cycle Evaluation

**Figure 4:**
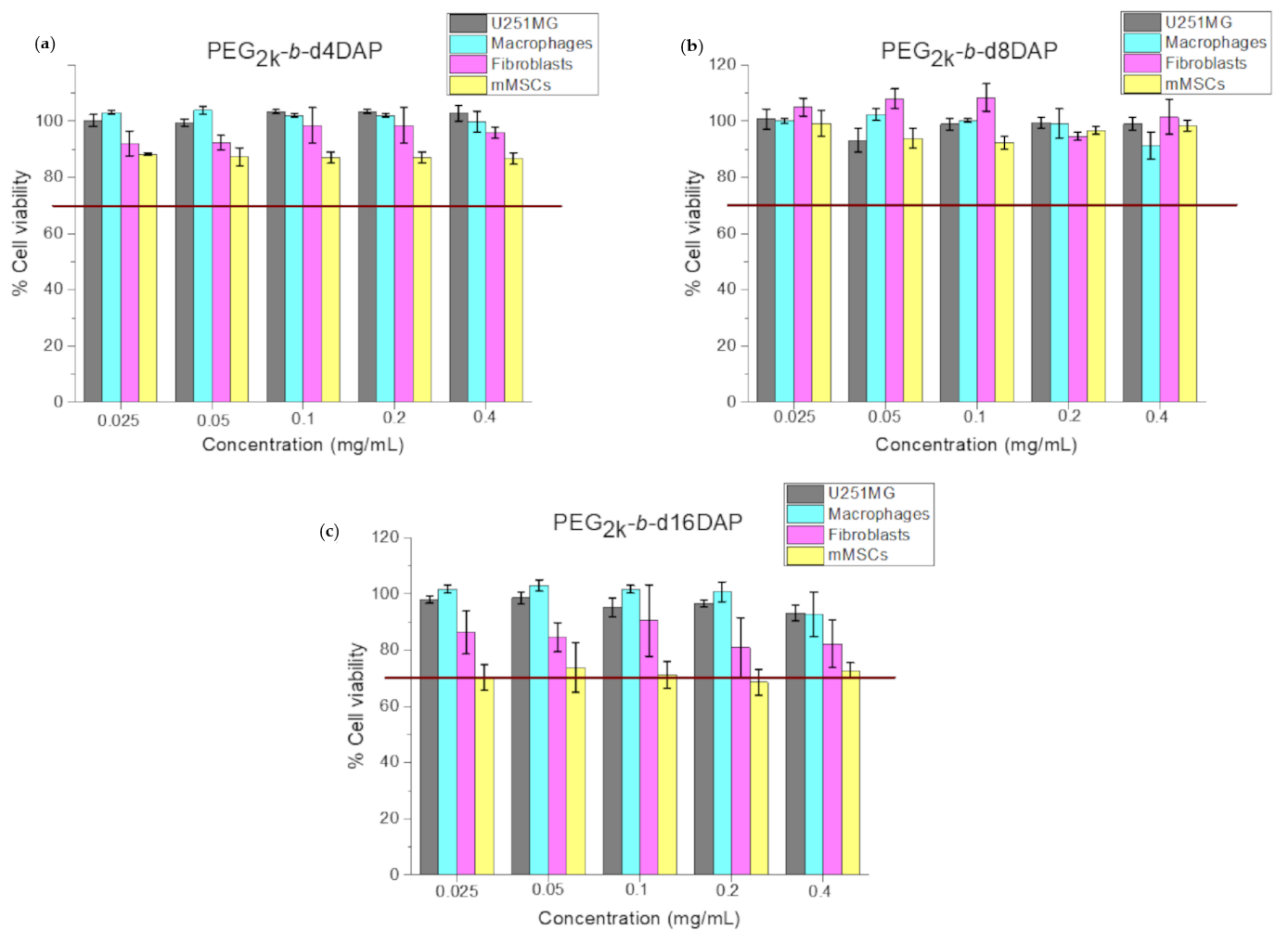
Cell viability of self-assemblies dispersion of LDBCs prepared by microfluidics (A, Table 3) adjusted at different polymer concentrations: (**a**) **PEG_2k_-*b*-d4DAP**, (**b**) **PEG_2k_-*b*-d8DAP**, and (**c**) **PEG_2k_-*b*-d16DAP** in the four cell lines assayed after 24 h. Red lines correspond to 70% cell viability, which is the minimum recommended for considering a subcytotoxic concentration according to ISO 10993-5 [61].

The cytotoxicity of **PEG_2k_-*b*-dxDAP** self-assemblies prepared by microfluidics employing **A** conditions, since it allowed obtaining the most concentrated samples (see Table 3), was studied in four cell lines (U251MG, human macrophages, human dermal fibroblasts, and mMSCs). Aqueous suspensions of **PEG_2k_-*b*-d4DAP**, **PEG_2k_-*b*-d8DAP**, and **PEG_2k_-*b*-d16DAP** self-assemblies (obtained by microfluids, experimental conditions A, Table 3) were assayed at different polymer concentrations that were adjusted from 0.025 to 0.4 mg mL^−1^. The results of cell viability estimated by the cell metabolism assay are represented in Figure 4. These results showed that cell viability is high (> 70%) for all the samples and all the concentrations assayed. Taking into account these results and the recommendations of the ISO 10993-5 [61], 0.4 mg mL^−1^, the maximum concentration assayed, was considered as the subcytotoxic concentration for further studies, as this concentration displayed viability percentages ≥70% in all the cell lines evaluated.

**Figure 5:**
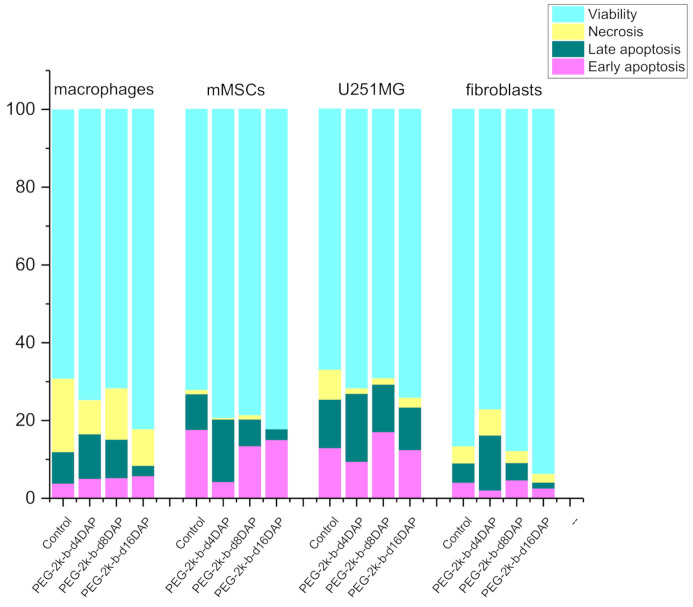
Distribution of early and late apoptotic, alive, and necrotic cells (percentage of cells, %) after treatment for 24 h with **PEG_2k_-*b*-dxDAP** aggregates obtained by microfluidics (A, Table 3) and with a polymer concentration adjusted at 0.4 mg mL^−1^.

Even though nanoparticles do not show cytotoxicity at the doses tested, they could disrupt cell cycle steps and cause DNA damage or apoptosis. Consequently, in order to know the effects of these nanoparticles on the cell membrane and nuclei, cell apoptosis and cell cycle studies were performed by flow cytometry. The results obtained from cell apoptosis studies, in order to know the potential cell membrane effects caused by **PEG_2k_-*b*-dxDAP** self-assemblies at a concentration of 0.4 mg mL^−1^, are represented in Figure 5. As it is shown, no remarkable changes compared to the control samples (non-treated cells) for any cell line were observed. In general, viability values were equal or higher than the ones determined in control assays. For macrophages, U251MG, and fibroblasts, necrosis percentages slightly decreased, and we observed a slight rise in the levels of apoptosis for macrophages, mMSCs, and fibroblasts when testing the **PEG_2k_-*b*-d4DAP** sample. Regardless, according to the recommendations indicated above of the ISO 10993-5 [61], **PEG_2k_-*b*-dxDAP** self-assemblies showed appropriate cytocompatibility for biomedical applications since their cytotoxic effect was always lower than 30% at the doses tested.

**Figure 6:**
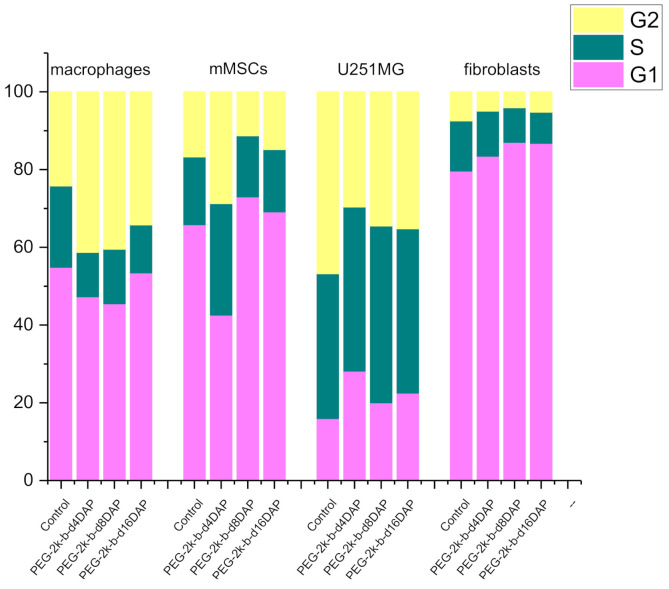
Cell cycle population distribution (percentage of cells, %) after treatment for 24 h with **PEG_2k_-*b*-dxDAP** aggregates obtained by microfluidics (A, Table 3) and with a polymer concentration adjusted to 0.4 mg mL^−1^.

Cell cycle results are depicted in Figure 6. For fibroblasts, not remarkable changes compared to control cells were observed. A slight increase in the G2 phase (<17%), together with a countervailing decrease in S and G1 phases, was observed for macrophages, which could indicate that **PEG_2k_-*b*-dxDAP** self-assemblies could induce cell cycle arrest in the G2 phase. In the case of mMSCs, the **PEG_2k_-*b*-d4DAP** sample caused a slight rise in the levels of G2 and S phases together with a decrease in the G1 phase (<25%). For U251MG, a slight decrease in the G2 phase (<17%), with a concomitant increase in S and G1 population, was observed. Anyway, changes compared to control cells depended on each cell line, but they were negligible.

### 3.5. Supramolecular Cross-Linking

The main structural feature of these LDBCs is the presence of DAP units at the periphery of the dendronized block and the ability of these units for the molecular recognition of complementary thymine units through highly specific three H-bonding interactions. Thus, these LDBCs could be used as a basic platform on which to incorporate functionality by supramolecular chemistry that is, in principle, simpler, more versatile, and less synthetically demanding than covalent chemistry.

One of the concerns about polymeric nanocarriers is the possibility of their premature destabilization in highly diluted physiological media. The integrity of BCs-based nanocarriers can be preserved by cross-linking either the hydrophobic core (or the inner membrane of polymersomes) or the hydrophilic crown of the self-assemblies [62]. Cross-linking strategies commonly rely on non-reversible covalent bonding [63], but the release of entrapped cargoes might be hindered, reducing any therapeutic effect. Alternatively, reversible or dynamic covalent bonds have been also used [64]. Likewise, non-covalent approaches coming from the supramolecular chemistry have been considered as an option to reduce the shortcoming of covalent cross-linking [65]. Amongst them, the less explored supramolecular cross-linking by hydrogen bonding might improve the stability of the polymeric nanocarriers without hampering the cargoes release, as this dynamic interaction can be reversibly broken and reconstituted. In this context, we have explored the potential versatility of **PEG_2k_-*b*-dxDAP** LDBCs by using the periphery of the dendrons for dynamic cross-linking using complementary thymine based cross-linkers. As a first proof of concept, we have prepared several H-bond cross-linked polymers from **PEG_2k_-*b*-dxDAP** and thymine-based cross-linkers (Scheme S1), and we evaluated whether it is possible to obtain stable aqueous self-assemblies. The effect of H-bond cross-linking on the self-assembly ability was evaluated using **PEG_2k_-*b*-d8DAP** and **PEG_2k_-*b*-d16DAP** as they yielded stable aggregates.

Two hydrophobic cross-linkers with either two (**thym-C_12_-thym**) or four (**(thym-C_6_)_4_-O**) thymine units were synthesized (Scheme 4). **Thym-C_12_-thym** was synthesized by the reaction of thymine with 1,12-dibromododecane, while **(Thym-C_6_)_4_-O** was synthesized by the reaction of pentaerythritol with 6-(5-methyl-2,4-dioxo-3,4-dihydro-2H-pyrimidin-1-yl)hexanoic acid **(5)**, which was prepared as previously [49]. These compounds were fully characterized by ^1^H-NMR, ^13^C-NMR, FTIR, and high-resolution electrospray ionization mass spectrometry HR-ESI MS (Figure S46). It was checked that none of the cross-linkers were able to form stable self-assemblies in water; in fact, a precipitate was formed by the slow addition of water to THF solutions of these thymine derivatives (under the same experimental conditions used on the nanoprecipitation experiments of LDBCs). Once we checked the thymine derivatives, we prepared supramolecular LDBCs by dissolving the corresponding amounts of **PEG_2k_-*b*-d8DAP** or **PEG_2k_-*b*-d16DAP**, and **thym-C_12_-thym** or **(thym-C_6_)_4_-O**, in THF to have a 1:1 DAP/thymine molar ratio, and the preparation of self-assemblies from these supramolecular LDBCs–thymine derivatives was approached by nanoprecipitation (Figure S47). In all cases, stable self-assemblies dispersions were obtained, and no evidence of a macroscopic precipitate of any of the components was observed; i.e., thymine derivatives do not precipitate under these experimental conditions and were incorporated onto the self-assemblies. Their morphology and size were investigated by TEM, cryo-TEM, and DLS, and CAC was also calculated.

**Figure 7:**
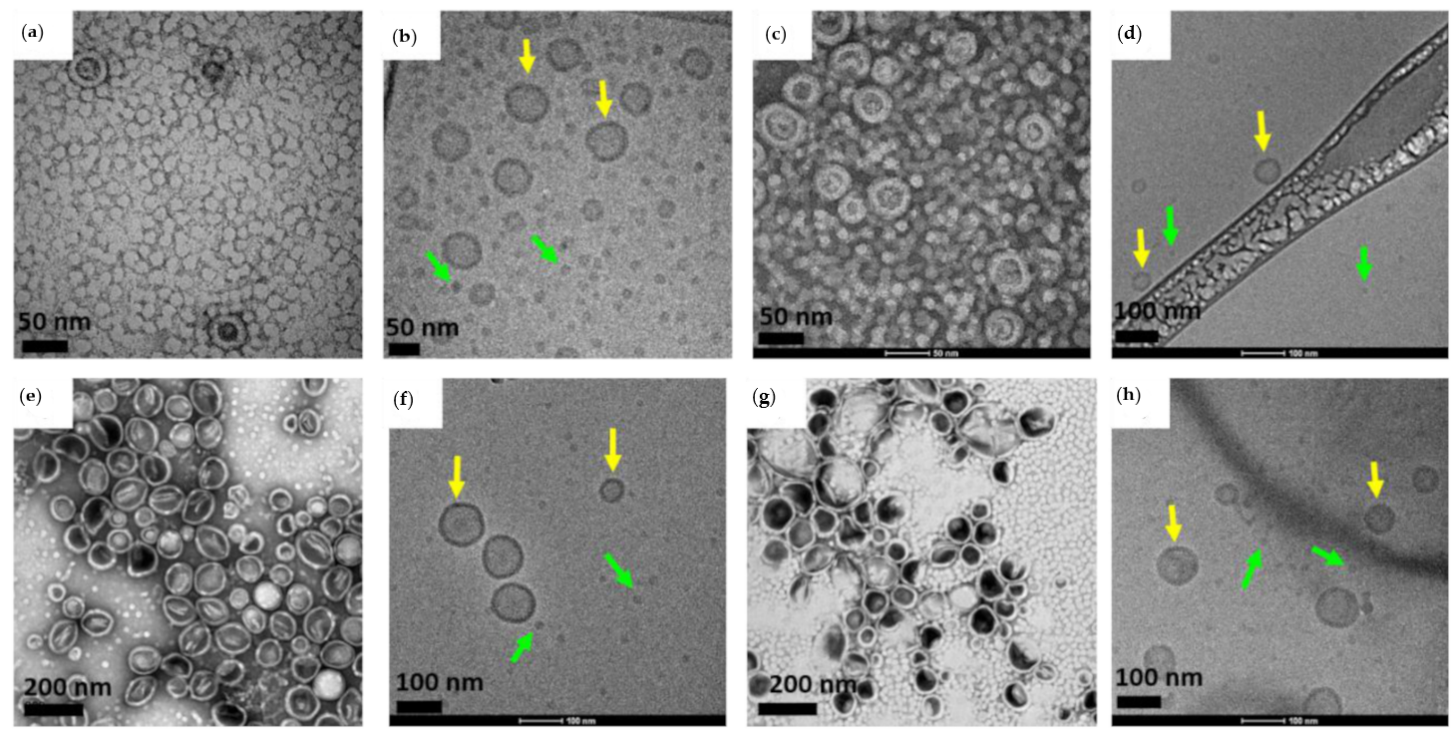
TEM images of (**a**) PEG_2k_-*b*-d8DAP·thym-C_12_-thym, (**c**) PEG_2k_-*b*-d8DAP·(thym-C_6_)_4_-O, (**e**) PEG_2k_-*b*-d16DAP·thym-C_12_-thym, and (**g**) PEG_2k_-*b*-d16DAP·(thym-C_6_)_4_-O; and cryo-TEM images of (**b**) PEG_2k_-*b*-d8DAP·thym-C_12_-thym, (**d**) PEG_2k_-*b*-d8DAP·(thym-C_6_)_4_-O, (**f**) PEG_2k_-*b*-d16DAP·thym-C_12_-thym and (**h**) PEG_2k_-*b*-d16DAP·(thym-C_6_)_4_-O self-assemblies (yellow arrows indicate vesicles and green arrows indicate micelles).

**Figure 8:**
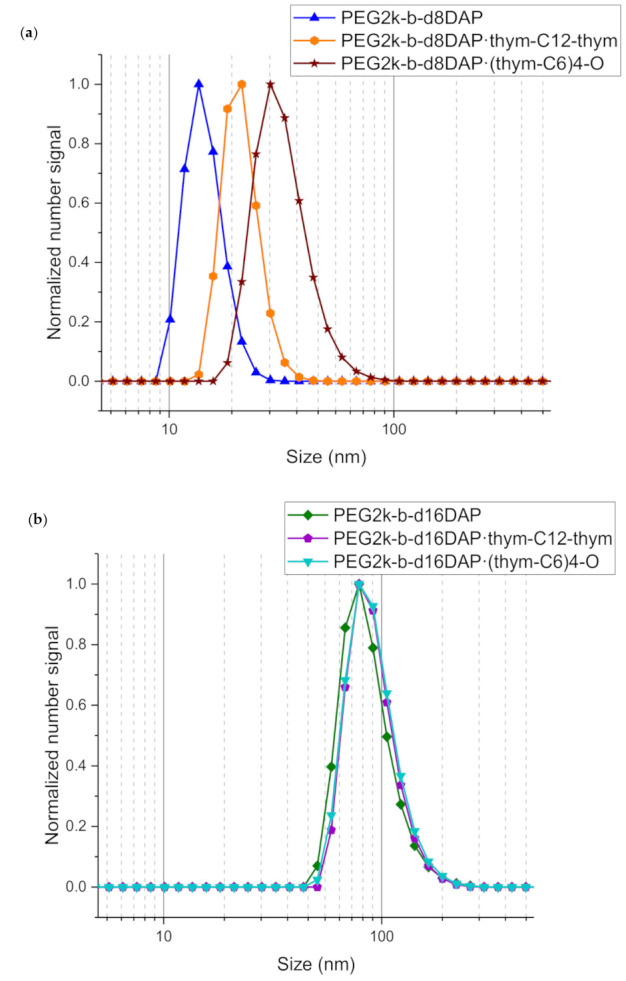
Number size distributions recorded by the DLS of supramolecular cross-linked LDBCs self-assemblies prepared by nanoprecipitation from either **PEG_2k_-*b*-d8DAP** (**a**) or **PEG_2k_-*b*-d16DAP** (**b**) and two thymine crosslinkers, either **thym-C_12_-thym** or (**(thym-C_6_)_4_-O**. The DLS of **PEG_2k_-*b*-d8DAP** and **PEG_2k_-*b*-d16DAP** self-assemblies (without incorporation of the supramolecular crosslinkers) are also included as reference.

TEM and cryo-TEM images revealed that **PEG_2k_-*b*-d8DAP·thym-C_12_-thym** and **PEG_2k_-*b*-d8DAP·(thym-C_6_)_4_-O** self-assembled, forming spherical micelles with diameters about 16 nm according TEM. Small vesicles were formed in both cases with diameters between 30 and 75 nm and membrane thicknesses of 9.3 ± 1.7 nm and 10.5 ± 1.8 nm for **PEG_2k_-*b*-d8DAP·thym-C_12_-thym** and **PEG_2k_-*b*-d8DAP·(thym-C_6_)_4_-O** according to cryo-TEM (Figure 7a–d). Size distribution curves registered by DLS revealed that apparent D_h_ values of cross-linked self-assemblies, 21 ± 7 nm and 31 ± 14 nm for **PEG_2k_-*b*-d8DAP·thym-C_12_-thym** and **PEG_2k_-*b*-d8DAP·(thym-C_6_)_4_-O** respectively, were higher than that of **PEG_2k_-*b*-d8DAP**, 14 ± 5 nm (Figure 8), although the contribution of both morphologies (micelles and vesicles) to DLS measurements did not allow a proper comparison of D_h_ values. CAC values were significantly lower for **PEG_2k_-*b*-d8DAP·thym-C_12_-thym** and **PEG_2k_-*b*-d8DAP·(thym-C_6_)_4_-O** than for the parent **PEG_2k_-*b*-d8DAP**, 22 and 16 µg mL^−1^ vs. 41 µg mL^−1^, respectively (Figure S48). The transition in morphology from micelles to vesicles could be explained by considering the increase on the hydrophobic-to-hydrophilic balance due to the incorporation of cross-linkers. In addition, the decrease of CAC values when cross-linkers are incorporated suggests a greater thermodynamic stability of self-assemblies.

When observed by TEM and cryo-TEM, **PEG_2k_-*b*-d16DAP·thym-C_12_-thym** and **PEG_2k_-*b*-d16DAP·(thym-C_6_)_4_-O** self-assembled, mainly forming polymeric vesicles, as the parent **PEG_2k_-*b*-d16DAP**. The membrane thickness measured by cryo-TEM was 12.4 ± 1.7 nm for **PEG_2k_-*b*-d16DAP·thym-C_12_-thym** and 14.8 ± 1.3 nm for **PEG_2k_-*b*-d16DAP·(thym-C_6_)_4_-O** (Figure 7e–g), which were higher values than that the estimated for **PEG_2k_-*b*-d16DAP**. Some spherical micelles were also observed with diameters of approximately 20–25 nm, according to TEM estimations. Apparent *D_h_* values determined by DLS were 91 ± 37 and 83 ± 39 nm for **PEG_2k_-*b*-d16DAP·thym-C_12_-thym** and **PEG_2k_-*b*-d16DAP·(thym-C_6_)_4_-O**, respectively, which were only slightly higher than for **PEG_2k_-*b*-d16DAP** (D_h_ = 79 ± 36 nm) (Figure 8). Accordingly, CAC values for **PEG_2k_-*b*-d16DAP·thym-C_12_-thym** and **PEG_2k_-*b*-d16DAP·(thym-C_6_)_4_-O** were 35 and 28 µg mL^−1^ (Figure S48). These values were slightly above that retrieved for **PEG_2k_-*b*-d16DAP** (25 µg mL^−1^). The slight increase in the size of the vesicles and, mainly, the increase in the thickness of the membrane are evidence of the inclusion of supramolecular cross-linkers in the hydrophobic internal part of the membrane of these vesicle self-assemblies.

Comparing both cross-linkers, the morphology of the self-assemblies is similar but CAC values are lower for self-assemblies cross-linked with **(thym-C_6_)_4_-O**, which suggests a comparative higher thermodynamic stability associated to a more efficient supramolecular cross-linking.

## 4. Conclusions

A series of amphiphilic LDBCs have been synthesized by the CuAAC coupling of a linear PEG block and the first fourth generations of dendrons derived from bis-MPA functionalized with 2,6-diaminopyridine (DAP) units at the periphery. A synthetic route for these dendrons has been developed based on a double exponential growth convergent approach. Their ability to self-assemble in water has been evaluated and depends on the dendron generation. Low dendron generations do not form stable self-assemblies, but for **PEG_2k_-*b*-d8DAP** and **PEG_2k_-*b*-d16DAP**, stable self-assemblies were obtained whose morphology depends on the generation and the self-assembly preparation technique. By nanoprecipitation, **PEG_2k_-*b*-d8DAP** and **PEG_2k_-*b*-d16DAP** yielded spherical micelles and vesicles, respectively. Self-assemblies have also been efficiently prepared by microfluidics as an alternative scalable and high throughput (725 mg h^−1^) methodology with a controlled and ultra-fast homogeneous mixing of fluids. The self-assembly study assisted by microfluidics has shown that the morphology and size of assemblies not only depend on the LDBC chemical structure but also on the water flow rate at a fixed mixing time as small as 48 milliseconds. In vitro cytotoxicity, metabolism, and cell cycle studies have been carried out concluding that these **PEG_2k_-*b*-dxDAP** self-assemblies are not cytotoxic at doses below 0.4 mg mL^−1^, opening new avenues for their use as drug delivery carriers.

The DAP ability of supramolecular functionalization was explored by incorporating thymine derivatives (**thym-C_12_-thym** and **(thym-C_6_)_4_-O**) as hydrogen bonding cross-linkers that were efficiently incorporated into the self-assemblies obtained in aqueous media. The supramolecular modification of these materials opens possibilities to the stabilization and functionalization of these assemblies based on the molecular recognition by H-bonding. Furthermore, the dynamic nature of the cross-links and their response to chemical stimuli as pH allow a faster and stimulated delivery than cross-linked nanocarriers based on covalent crosslinks.

## Figures and Tables

**Table 1:** Molar mass, dispersity, and hydrophobic/hydrophilic ratio of the synthesized LDBCs.

Block Copolymer	M_n_^a^ (calc.) g mol^−1^	M_n_^b^ g mol^−1^	M_n_^c^ (GPC) g mol^−1^	*Đ* ^c^	Hydrophobic/Hydrophilic Ratio ^d^
**PEG_2k_-*b*-d2DAP**	2737.2	2737.9	4252	1.01	28/72
**PEG_2k_-*b*-d4DAP**	3463.8	3509.8	4434	1.03	43/57
**PEG_2k_-*b*-d8DAP**	4917.5	4919.8	5427	1.02	60/40
**PEG_2k_-*b*-d16DAP**	7814.1	7720.9	6813	1.01	75/25

^a^ Theoretical number average molar mass (Mn) calculated as the sum of the molar mass of polyethylene glycol (PEG) block (Mn = 1983.9 g mol−1, determined by MALDI-TOF and corresponding to the more intense m/z peak) and the molar mass of the monodisperse dendritic block. ^b^ Mn determined by MALDI-TOF corresponding to the more intense m/z peak. ^c^ Relative Mn and dispersity (Đ) determined by Gel Permeation Chromatography (GPC) using THF (1 mL min−1) and polymethacrylate (PMMA) standards calibration. ^d^ Approximate hydrophobic/hydrophilic weight percentage ratio calculated from Mn (calc.), considering the dendritic block as the hydrophobic part and the linear block (PEG) as the hydrophilic part.

**Table 2:** Thermal properties of dendrons and linear–dendritic block copolymers (LDBCs).

Sample	T_onset_ (°C) ^a^	T_g_ (°C) ^b^	T_m_ (°C) ^c^
**N_3_**-**d2DAP**	253	19	-
**N_3_**-**d4DAP**	238	30	-
**N_3_**-**d8DAP**	249	71	-
**N_3_**-**d16DAP**	253	73	-
**PEG_2k_-*b*-d2DAP**	262	-	43 ^d^
**PEG_2k_-*b*-d4DAP**	241	-	41 ^e^
**PEG_2k_-*b*-d8DAP**	263	−14	-
**PEG_2k_-*b*-d16DAP**	256	−11	- ^f^

^a^ Decomposition temperature determined by thermogravimetric analysis (TGA) given as the onset of the peak recorded at the first derivative of the mass loss curve. ^b^ Glass transition temperature calculated at the half height of the baseline jump on the second heating scan at 10 °C min^−1^. ^c^ Melting temperature given at the maximum of the peak on the second heating scan at 10 °C min^−1^. ^d^ Polymer partially crystallizes during the cooling process at 0 °C. On heating, cold crystallization is observed at ‒30 °C. ^e^ Cold crystallization process at ‒10 °C. ^f^ Melting of a small crystalline fraction at 44 °C was observed.

**Table 3:** Experimental preparation conditions of LDBCs self-assemblies by microfluidics: phase ratio values (aqueous solution/organic solution percentage) used with a flow rate fixed at 10 mL min^−1^ and final polymer concentration.

Experimental Conditions	Aqueous/Organic Solution Phase Ratio ^a^	Polymer Concentration (mg mL^−1^) ^b^
**A**	60/40	2.00
**B**	70/30	1.50
**C**	80/20	1.00
**D**	90/10	0.50
**E**	95/5	0.25

^a^ Ratio between Milli-Q^®^ water and a 5 mg mL^−1^ THF solution of each LDBC fed into the micromixer. ^b^ Polymer concentration in the self-assemblies dispersions collected at the micromixer outlet.

## Data Availability

The data presented in this study are available in this article and its Supplementary Materials.

## Supplementary Materials

The following are available online at https://www.mdpi.com/2073-4360/13/5/684/s1, Figure S1: MALDI-TOF spectra of (a) PEG_2k_-OH and (b) PEG_2k_-Alky. Figure S2-35: FTIR and ^1^H-NMR spectra of all the compounds synthesized. Figure S36: (a) HR-ESI spectrum of N_3_-d2DAP. (b) MALDI-TOF spectra of N_3_-d4DAP. Figure S37: MALDI-TOF spectra of (a) N_3_-d8DAP and (b) N_3_-d16DAP. Figure S38: MALDI-TOF spectra of (a) PEG_2k_-*b*-d2DAP and (b) PEG_2k_-*b*-d4DAP. Figure S39: MALDI-TOF spectra of (a) PEG_2k_-*b*-d8DAP and (b) PEG_2k_-*b*-d16DAP. Figure S40: GPC chromatograms for (a) dendrons N_3_dxDAP and (b) LDBCs PEG_2k_-*b*-dxDAP. Figure S41: DSC curves registered on first cooling (above) and second heating (below) at 10 °C min^−1^ scanning rate of N_3_dxDAP. Figure S42: DSC curves registered on first heating (above), first cooling (middle), and second heating (below) at a 10 °C min^−1^ scanning rate of PEG_2k_-*b*-dxDAP. Figure S43: Turbidity curves of LDBCs PEG_2k_-*b*-dxDAP. Figure S44: Intensity size distributions by DLS of LDBCs self-assemblies prepared by nanoprecipitation. Figure S45: Fluorescence emission of Nile Red at 620 nm (λ_exc_ = 550 nm) versus (a) PEG_2k_-*b*-d4DAP, (b) PEG_2k_-*b*-d8DAP and (c) PEG_2k_-*b*-d16DAP concentration. CAC was determined from the intersection of the two extrapolated lines. Scheme S1: Supramolecular recognition through a triple H-bond between DAP units (blue) linked at the periphery of dendron block of PEG2k-b-dxDAP and thymine moieties (black) of the crosslinkers. Figure S46: HR-ESI spectra of (a) thym-C_12_-thym and (b) (thym-C_6_)_4_-O. Figure S47: Turbidity curves of supramolecular cross-linked LDBCs of PEG_2k_-*b*-d8DAP (left) and PEG_2k_-*b*-d16DAP (right). Figure S48: Fluorescence emission of Nile Red at 620 nm (λ_exc_ = 550 nm) versus (a) PEG2k-b-d8DAP·thym-C12-thym, (b) PEG2k-b-d8DAP·(thym-C6)4-O, (c) PEG2k-b-d16DAP·thym-C12-thym and PEG2k-b-d16DAP·(thym-C6)4-O concentration. CAC was determined from the intersection of the two extrapolated lines.

## References

[1] Lazzari M., Liu G., Lecommandoux S. (2006). Block Copolymers in Nanoscience.

[2] Jain S., Bates F.S. (2003). On the origins of morphological complexity in block copolymer surfactants. Science.

[3] Elsabahy M., Wooley K.L. (2012). Design of polymeric nanoparticles for biomedical delivery applications. Chem. Soc. Rev..

[4] Mintzer M.A., Grinstaff M.W. (2011). Biomedical applications of dendrimers: A tutorial. Chem. Soc. Rev..

[5] Astruc D., Boisselier E., Ornelas C. (2010). Dendrimers designed for functions: From physical, photophysical, and supramolecular properties to applications in sensing, catalysis, molecular electronics, photonics, and nanomedicine. Chem. Rev..

[6] Wurm F., Frey H. (2011). Linear-dendritic block copolymers: The state of the art and exciting perspectives. Prog. Polym. Sci..

[7] Kolb H.C., Finn M.G., Sharpless K.B. (2001). Click Chemistry: Diverse Chemical Function from a Few Good Reactions. Angew. Chem. Int. Ed..

[8] Hua C., Peng S.M., Dong C.M. (2008). Synthesis and characterization of linear-dendron-like poly(ε-caprolactone)-b-poly(ethylene oxide) copolymers via the combination of ring-opening polymerization and click chemistry. Macromolecules.

[9] Del Barrio J., Oriol L., Alcalá R., Sánchez C. (2009). Azobenzene-Containing linear-Dendritic diblock copolymers by click chemistry: Synthesis, characterization, morphological study, and photoinduction of optical anisotropy. Macromolecules.

[10] Blasco E., Piñol M., Oriol L. (2014). Responsive Linear-Dendritic Block Copolymers. Macromol. Rapid Commun..

[11] Mongkhontreerat S., Walter M.V., Cai Y., Brismar H., Hult A., Malkoch M. (2015). Functional porous membranes from amorphous linear dendritic polyester hybrids. Polym. Chem..

[12] Mongkhontreerat S., Walter M.V., Andrén O.C.J., Cai Y., Malkoch M. (2015). Beyond State of the Art Honeycomb Membranes: High Performance Ordered Arrays from Multiprogrammable Linear-Dendritic Block Copolymers. Adv. Funct. Mater..

[13] Liu X., Monzavi T., Gitsov I. (2019). Controlled ATRP synthesis of novel linear- dendritic block copolymers and their directed self- assembly in breath figure arrays. Polymers.

[14] Liu X., Gitsov I. (2019). Nonionic amphiphilic linear dendritic block copolymers. solvent-induced self-Assembly and morphology tuning. Macromolecules.

[15] Sousa-Herves A., Riguera R., Fernandez-Megia E. (2012). PEG-dendritic block copolymers for biomedical applications. New J. Chem..

[16] Dong C.M., Liu G. (2013). Linear-dendritic biodegradable block copolymers: From synthesis to application in bionanotechnology. Polym. Chem..

[17] Whitton G., Gillies E.R. (2015). Functional aqueous assemblies of linear-dendron hybrids. J. Polym. Sci. Part A Polym. Chem..

[18] Fan X., Zhao Y., Xu W., Li L. (2016). Linear-dendritic block copolymer for drug and gene delivery. Mater. Sci. Eng. C.

[19] Matsumura Y., Maeda H. (1986). A New Concept for Macromolecular Therapeutics in Cancer Chemotherapy: Mechanism of Tumoritropic Accumulation of Proteins and the Antitumor Agent Smancs. Cancer Res..

[20] Van Hest J.C.M., Delnoye D.A.P., Baars M.W.P.L., Van Genderen M.H.P., Meijer E.W. (1995). Polystyrene-dendrimer amphiphilic block copolymers with a generation-dependent aggregation. Science.

[21] Lebedeva I.O., Zhulina E.B., Borisov O.V. (2018). Theory of Linear-Dendritic Block Copolymer Micelles. ACS Macro Lett..

[22] Lebedeva I.O., Zhulina E.B., Borisov O.V. (2019). Self-Assembly of Linear-Dendritic and Double Dendritic Block Copolymers: From Dendromicelles to Dendrimersomes. Macromolecules.

[23] Del Barrio J., Oriol L., Sánchez C., Serrano J.L., Di Cicco A., Keller P., Li M.H. (2010). Self-assembly of linear-dendritic diblock copolymers: From nanofibers to polymersomes. J. Am. Chem. Soc..

[24] Lin Y.L., Chang H.Y., Sheng Y.J., Tsao H.K. (2012). Photoresponsive polymersomes formed by amphiphilic linear-dendritic block copolymers: Generation-dependent aggregation behavior. Macromolecules.

[25] Blasco E., Del Barrio J., Sánchez-Somolinos C., Piñol M., Oriol L. (2013). Light induced molecular release from vesicles based on amphiphilic linear-dendritic block copolymers. Polym. Chem..

[26] Torres Neus F., Walter M.V., Montañez M.I., Kunzmann A., Hult A., Nyström A.M., Malkoch M., Fadeel B. (2012). Biocompatibility of polyester dendrimers in comparison to polyamidoamine dendrimers. Toxicol. Lett..

[27] Andrén O.C.J., Zhang Y., Lundberg P., Hawker C.J., Nyström A.M., Malkoch M. (2017). Therapeutic Nanocarriers via Cholesterol Directed Self-Assembly of Well-Defined Linear-Dendritic Polymeric Amphiphiles. Chem. Mater..

[28] Kalva N., Parekh N., Ambade A.V. (2015). Controlled micellar disassembly of photo- and pH-cleavable linear-dendritic block copolymers. Polym. Chem..

[29] Fedeli E., Lancelot A., Dominguez J.M., Serrano J.L., Calvo P., Sierra T. (2019). Self-assembling hybrid linear-dendritic block copolymers: The design of nano-carriers for lipophilic antitumoral drugs. Nanomaterials.

[30] Kosakowska K.A., Casey B.K., Kurtz S.L., Lawson L.B., Grayson S.M. (2018). Evaluation of Amphiphilic Star/Linear-Dendritic Polymer Reverse Micelles for Transdermal Drug Delivery: Directing Carrier Properties by Tailoring Core versus Peripheral Branching. Biomacromolecules.

[31] Kosakowska K.A., Casey B.K., Albert J.N.L., Wang Y., Ashbaugh H.S., Grayson S.M. (2018). Synthesis and Self-Assembly of Amphiphilic Star/Linear-Dendritic Polymers: Effect of Core versus Peripheral Branching on Reverse Micelle Aggregation. Biomacromolecules.

[32] Rosenbaum I., Harnoy A.J., Tirosh E., Buzhor M., Segal M., Frid L., Shaharabani R., Avinery R., Beck R., Amir R.J. (2015). Encapsulation and covalent binding of molecular payload in enzymatically activated micellar nanocarriers. J. Am. Chem. Soc..

[33] Harnoy A.J., Buzhor M., Tirosh E., Shaharabani R., Beck R., Amir R.J. (2017). Modular Synthetic Approach for Adjusting the Disassembly Rates of Enzyme-Responsive Polymeric Micelles. Biomacromolecules.

[34] Zhou K., Nguyen L.H., Miller J.B., Yan Y., Kos P., Xiong H., Li L., Hao J., Minnig J.T., Zhu H. (2016). Modular degradable dendrimers enable small RNAs to extend survival in an aggressive liver cancer model. Proc. Natl. Acad. Sci. USA.

[35] Zhou K., Johnson L.T., Xiong H., Barrios S., Minnig J.T., Yan Y., Abram B., Yu X., Siegwart D.J. (2020). Hydrophobic Domain Structure of Linear-Dendritic Poly(ethylene glycol) Lipids Affects RNA Delivery of Lipid Nanoparticles. Mol. Pharm..

[36] Milton Harris J., Chess R.B. (2003). Effect of pegylation on pharmaceuticals. Nat. Rev. Drug Discov..

[37] Jain N.K., Nahar M. (2010). PEGylated nanocarriers for systemic delivery. Methods Mol. Biol..

[38] Qian Y., You D., Lin F., Wei J., Wang Y., Bi Y. (2019). Enzyme triggered disassembly of amphiphilic linear-dendritic block copolymer micelles based on poly[: N -(2-hydroxyethyl-l-glutamine)]. Polym. Chem..

[39] Wei J., Lin F., You D., Qian Y., Wang Y., Bi Y. (2019). Self-assembly and enzyme responsiveness of amphiphilic linear-dendritic block copolymers based on poly(N-vinylpyrrolidone) and dendritic phenylalanyl-lysine dipeptides. Polymers.

[40] Concellón A., Clavería-Gimeno R., Velázquez-Campoy A., Abian O., Piñol M., Oriol L. (2016). Polymeric micelles from block copolymers containing 2,6-diacylaminopyridine units for encapsulation of hydrophobic drugs. RSC Adv..

[41] Concellón A., Blasco E., Martínez-Felipe A., Martínez J.C., Šics I., Ezquerra T.A., Nogales A., Piñol M., Oriol L. (2016). Light-Responsive Self-Assembled Materials by Supramolecular Post-Functionalization via Hydrogen Bonding of Amphiphilic Block Copolymers. Macromolecules.

[42] Liu Y., Yang G., Baby T., Chen D., Weitz D.A., Zhao C.X., Tengjisi (2020). Stable Polymer Nanoparticles with Exceptionally High Drug Loading by Sequential Nanoprecipitation. Angew. Chem. Int. Ed..

[43] Liu Y., Yang G., Zou D., Hui Y., Nigam K., Middelberg A.P.J., Zhao C.X. (2020). Formulation of Nanoparticles Using Mixing-Induced Nanoprecipitation for Drug Delivery. Ind. Eng. Chem. Res..

[44] Jiang Z., Liu H., He H., Ribbe A.E., Thayumanavan S. (2020). Blended Assemblies of Amphiphilic Random and Block Copolymers for Tunable Encapsulation and Release of Hydrophobic Guest Molecules. Macromolecules.

[45] Macedo A.S., Carvalho E.O., Cardoso V.F., Correia D.M., Tubio C.R., Fidalgo-Marijuan A., Botelho G., Lanceros-Méndez S. (2020). Tailoring electroactive poly(vinylidene fluoride-co-trifluoroethylene) microspheres by a nanoprecipitation method. Mater. Lett..

[46] Garcia-Salinas S., Himawan E., Mendoza G., Arruebo M., Sebastian V. (2018). Rapid on-Chip Assembly of Niosomes: Batch versus Continuous Flow Reactors. ACS Appl. Mater. Interfaces.

[47] Blasco E., Del Barrio J., Piñol M., Oriol L., Berges C., Sánchez C., Alcalá R. (2012). Azobenzene-containing linear-dendritic block copolymers prepared by sequential ATRP and click chemistry. Polymer (Guildf.).

[48] Concellón A., Blasco E., Piñol M., Oriol L., Díez I., Berges C., Sánchez-Somolinos C., Alcalá R. (2014). Photoresponsive polymers and block copolymers by molecular recognition based on multiple hydrogen bonds. J. Polym. Sci. Part A Polym. Chem..

[49] Higley M.N., Pollino J.M., Hollembeak E., Weck M. (2005). A modular approach toward block copolymers. Chem. A Eur. J..

[50] Löber S., Rodriguez-Loaiza P., Gmeiner P. (2003). Click linker: Efficient and high-yielding synthesis of a new family of SPOS resins by 1,3-dipolar cycloaddition. Org. Lett..

[51] De Solorzano I.O., Uson L., Larrea A., Miana M., Sebastian V., Arruebo M. (2016). Continuous synthesis of drug-loaded nanoparticles using microchannel emulsification and numerical modeling: Effect of passive mixing. Int. J. Nanomed..

[52] Kawaguchi T., Moore J.S., Walker K.L., Wilkins C.L. (1995). Double Exponential Dendrimer Growth. J. Am. Chem. Soc..

[53] Roche A., Oriol L., Tejedor R.M., Piñol M. (2019). Polymeric self-assemblies based on tetra-ortho-substituted azobenzene as visible light responsive nanocarriers. Polymers.

[54] Choucair A., Lavigueur C., Eisenberg A. (2004). Polystyrene-b-poly(acrylic acid) vesicle size control using solution properties and hydrophilic block length. Langmuir.

[55] Rainbolt E.A., Washington K.E., Biewer M.C., Stefan M.C. (2015). Recent developments in micellar drug carriers featuring substituted poly(ε-caprolactone)s. Polym. Chem..

[56] Chang Y., Kwon Y.C., Lee S.C., Kim C. (2000). Amphiphilic linear PEO-dendritic carbosilane block copolymers. Macromolecules.

[57] Mynar J.L., Goodwin A.P., Cohen J.A., Ma Y., Fleming G.R., Fréchet J.M.J. (2007). Two-photon degradable supramolecular assemblies of linear-dendritic copolymers. Chem. Commun..

[58] Park J.I., Saffari A., Kumar S., Günther A., Kumacheva E. (2010). Microfluidic synthesis of polymer and inorganic particulate materials. Annu. Rev. Mater. Res..

[59] Marre S., Jensen K.F. (2010). Synthesis of micro and nanostructures in microfluidic systems. Chem. Soc. Rev..

[60] Sebastián V., Khan S.A., Kulkarni A.A. (2017). Perspective article: Flow synthesis of functional materials. J. Flow Chem..

[61] ISO (2009). ISO 10993-5:2009 Biological Evaluation of Medical Devices—Part 5: Tests for in Vitro Cytotoxicity.

[62] Talelli M., Barz M., Rijcken C.J.F., Kiessling F., Hennink W.E., Lammers T. (2015). Core-crosslinked polymeric micelles: Principles, preparation, biomedical applications and clinical translation. Nano Today.

[63] Zhou Z., Forbes R.T., D’Emanuele A. (2017). Preparation of core-crosslinked linear-dendritic copolymer micelles with enhanced stability and their application for drug solubilisation. Int. J. Pharm..

[64] Li Y., Xiao K., Luo J., Xiao W., Lee J.S., Gonik A.M., Kato J., Dong T.A., Lam K.S. (2011). Well-defined, reversible disulfide cross-linked micelles for on-demand paclitaxel delivery. Biomaterials.

[65] Shi Y., Lammers T., Storm G., Hennink W.E. (2017). Physico-Chemical Strategies to Enhance Stability and Drug Retention of Polymeric Micelles for Tumor-Targeted Drug Delivery. Macromol. Biosci..

