# Supplementary material for: Supramolecular Functionalizable Linear–Dendritic Block Copolymers for the Preparation of Nanocarriers by Microfluidics
Source: Polymers (Basel). 2021 Feb 25;13(5):684. doi: 10.3390/polym13050684 (PMC7956801; doi:10.3390/polym13050684)
Supplement: Supplementary file 1 [file polymers-13-00684-s001.pdf]

Supplementary Materials

Supramolecular Functionalizable Linear-Dendritic Block Copolymers for the Preparation of Nanocarriers by Microfluidics

Miriam Abad [1,2], Alejandro Martínez-Bueno [1,2], Gracia Mendoza [1,3,4,5], Manuel Arruebo [1,3,4,5], Luis Oriol [1,2], Víctor Sebastián [1,3,4,5]* and Milagros Piñol [1,2]*

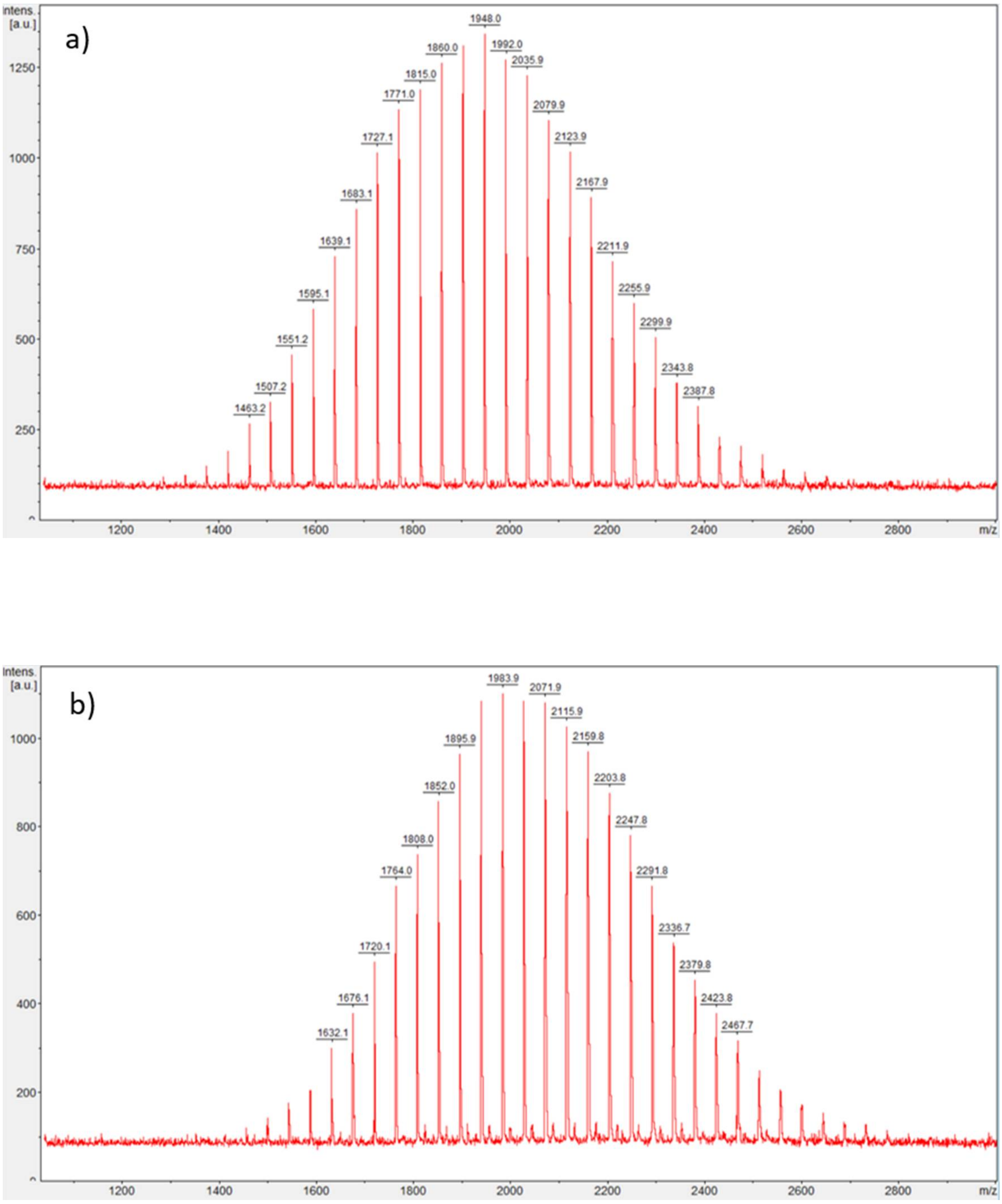

Figure S1. MALDI-TOF spectra of a) **PEG2k-OH** and b) **PEG2k-Alky**.

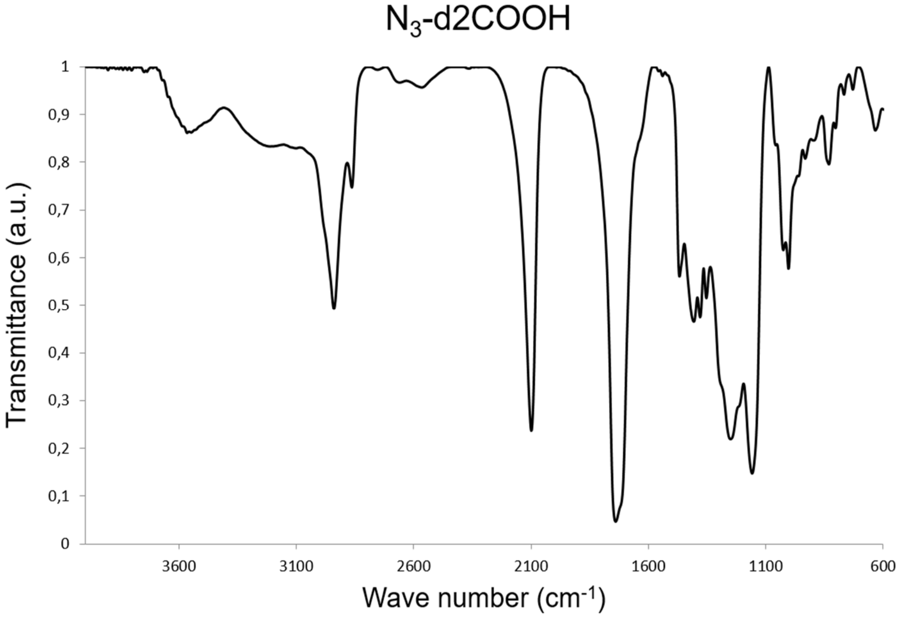

Figure S2 FTIR spectrum of **N_3-d2COOH**.

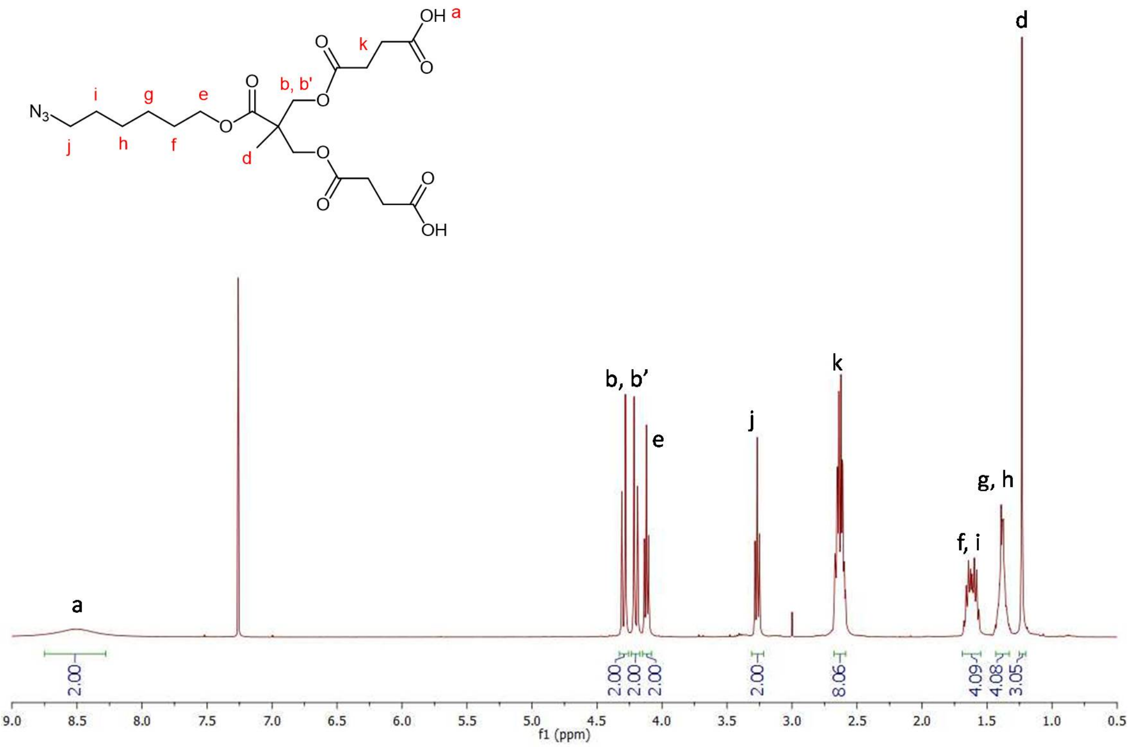

Figure S3. ^{1}H-NMR spectrum of **N_3-d2COOH** (CDCl3, 400MHz) δ (ppm).

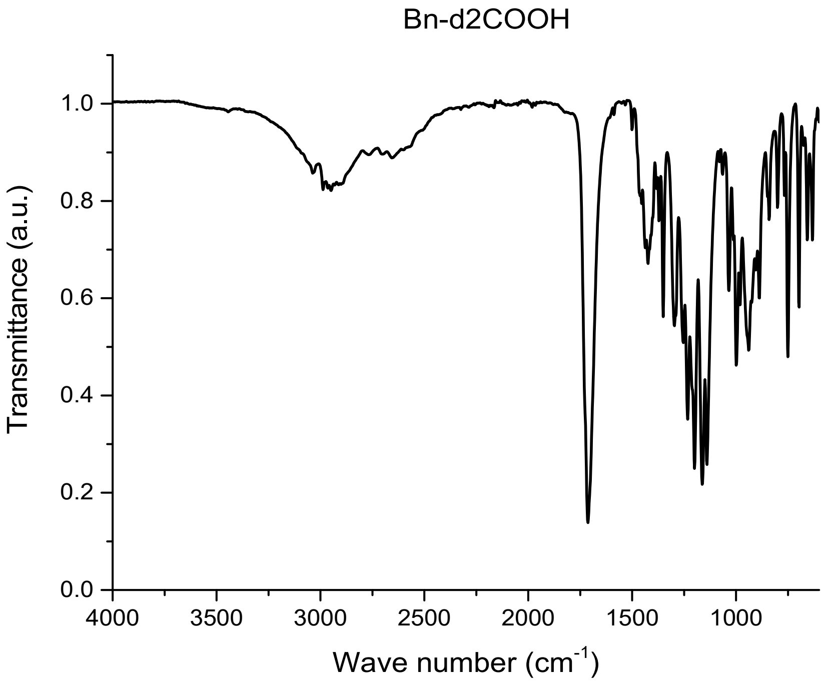

Figure S4. FTIR spectrum of **Bn-d2COOH**.

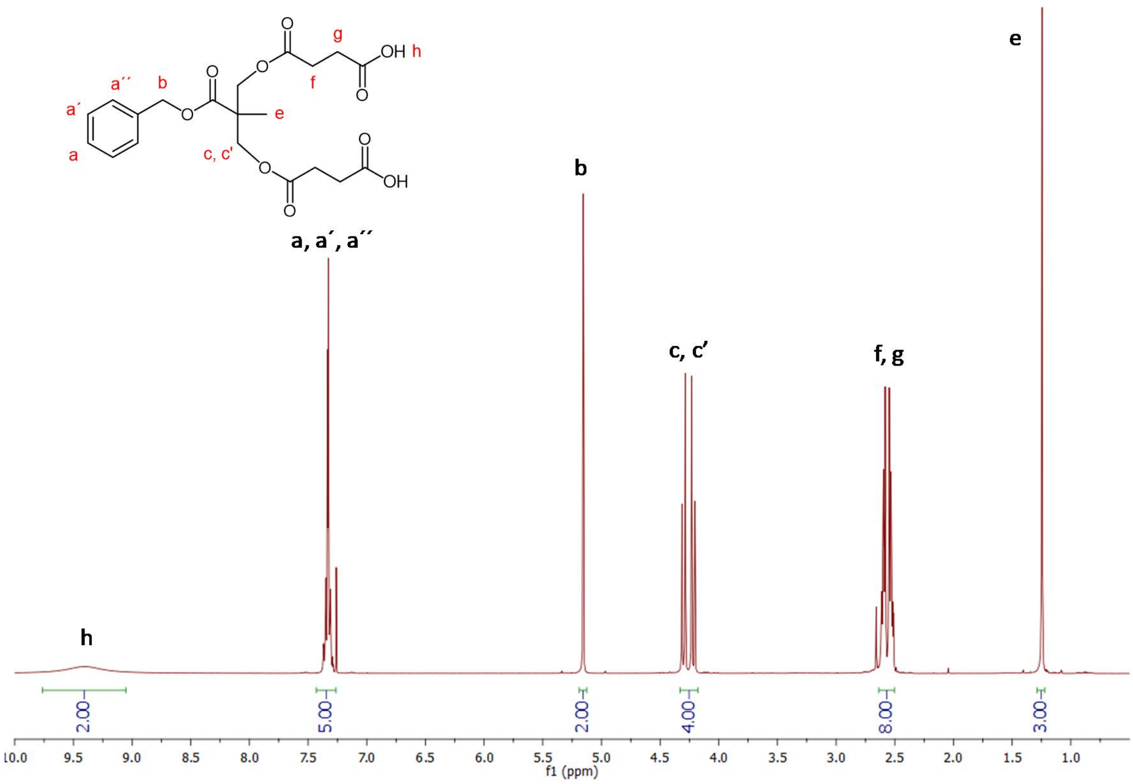

Figure S5. ^{1}H-NMR spectrum of **Bn-d2COOH** (CDCl3, 400MHz) δ (ppm).

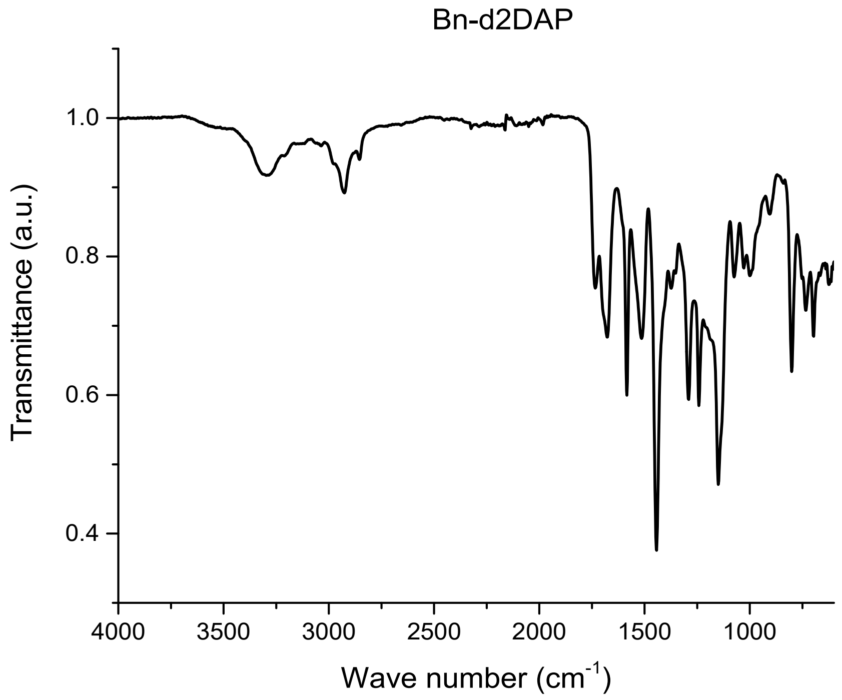

Figure S6. FTIR spectrum of **Bn-d2DAP**.

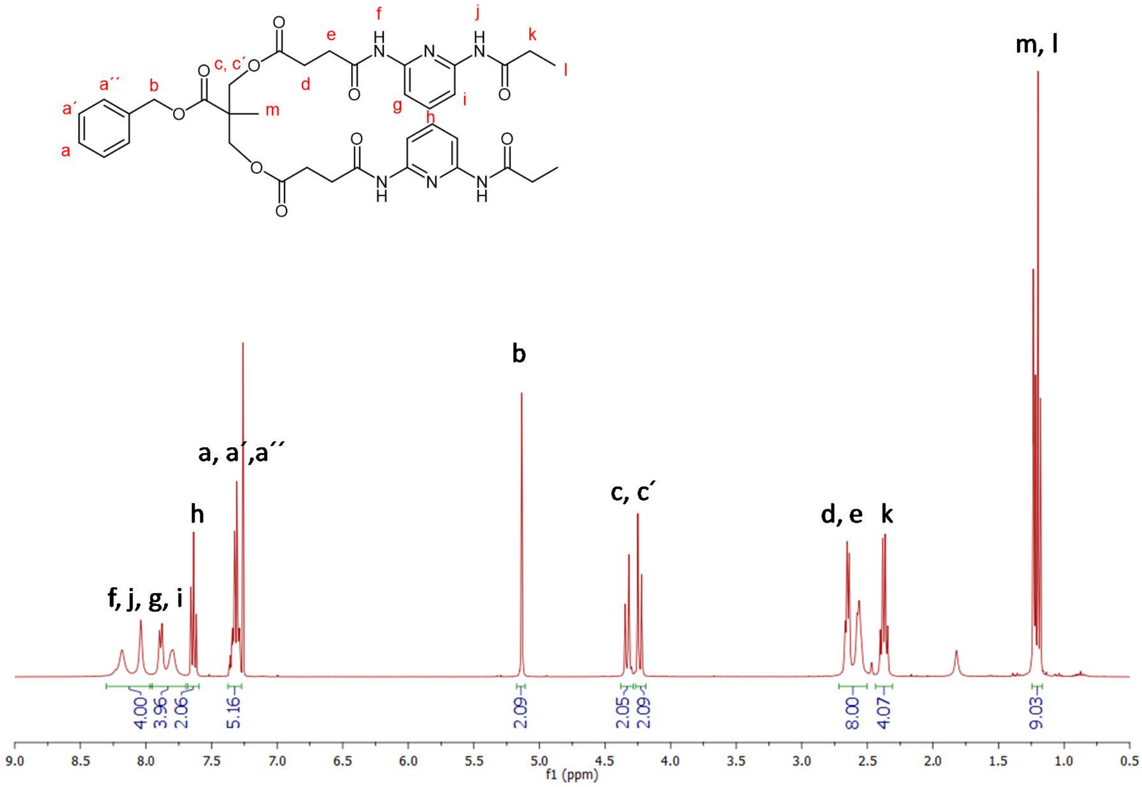

Figure S7. ^{1}H-NMR spectrum of **Bn-d2DAP** (CDCl3, 400MHz) δ (ppm).

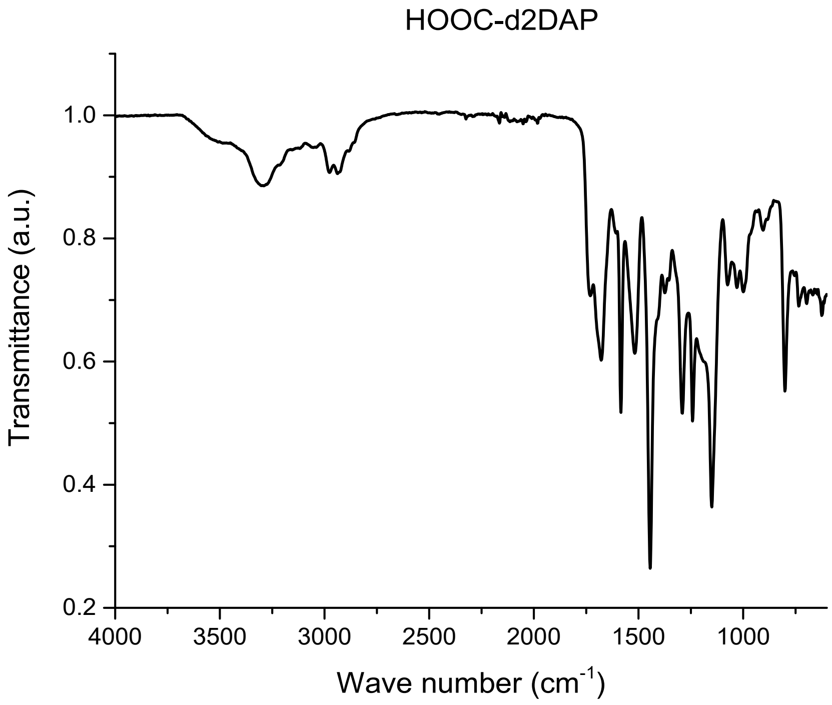

Figure S8. FTIR spectrum of **HOOC-d2DAP**.

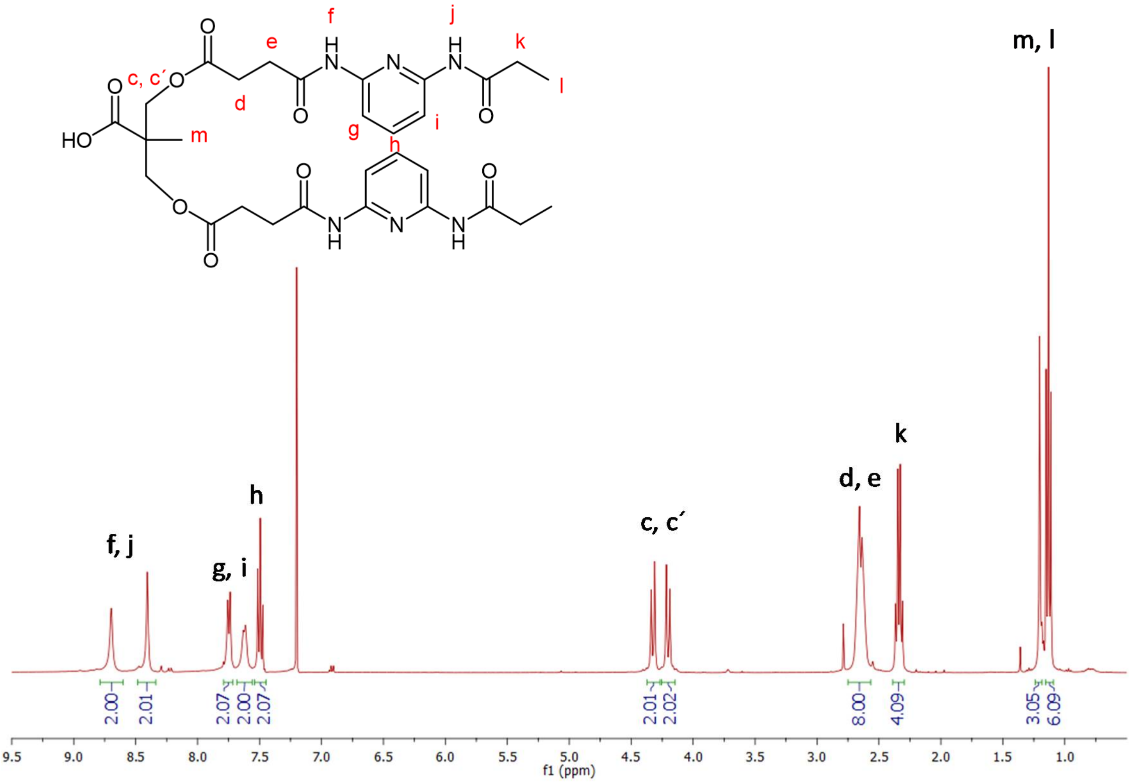

Figure S9. ^{1}H-NMR spectrum of **HOOC-d2DAP** (CDCl3, 400MHz) δ (ppm).

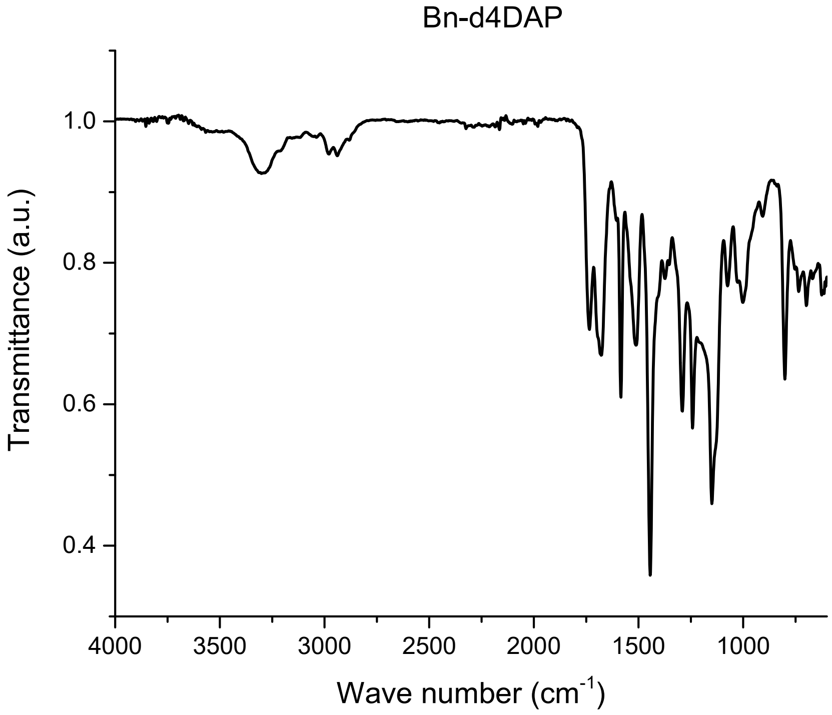

Figure S10. FTIR spectrum of **Bn-d4DAP**.

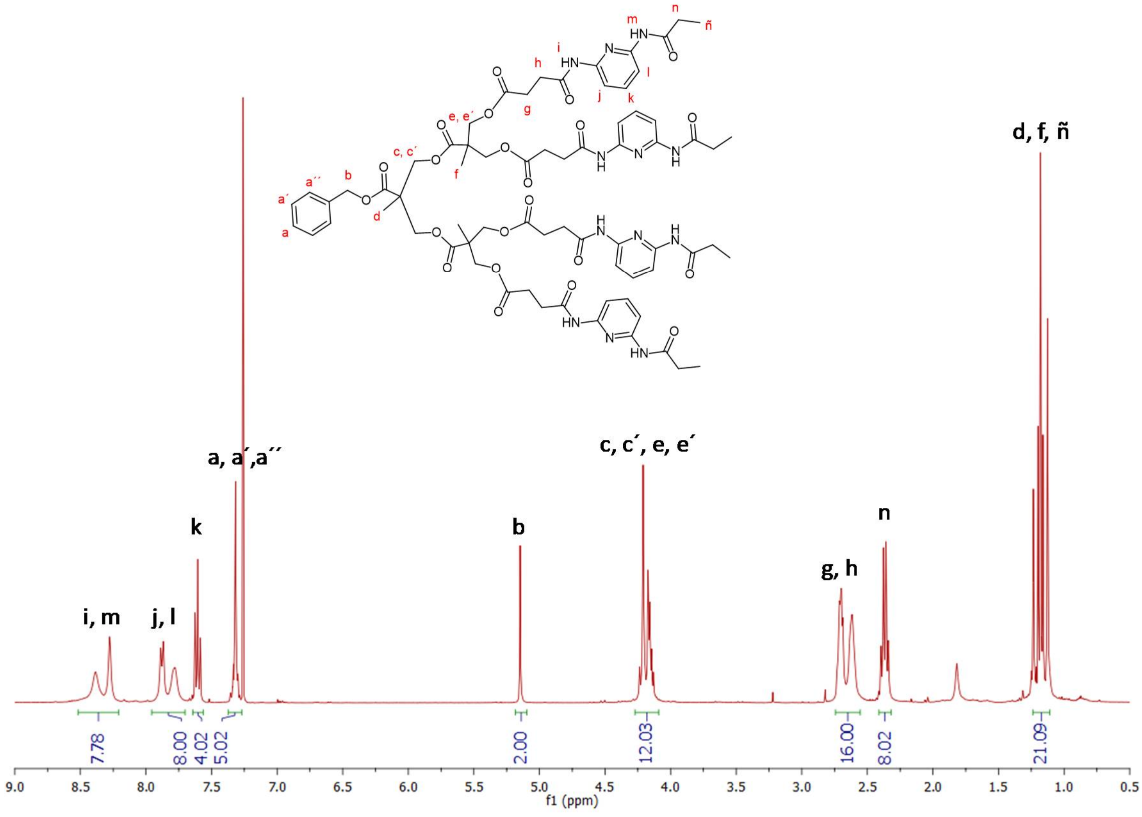

Figure S11. ^{1}H-NMR spectrum of **Bn-d4DAP** (CDCl3, 400MHz) δ (ppm).

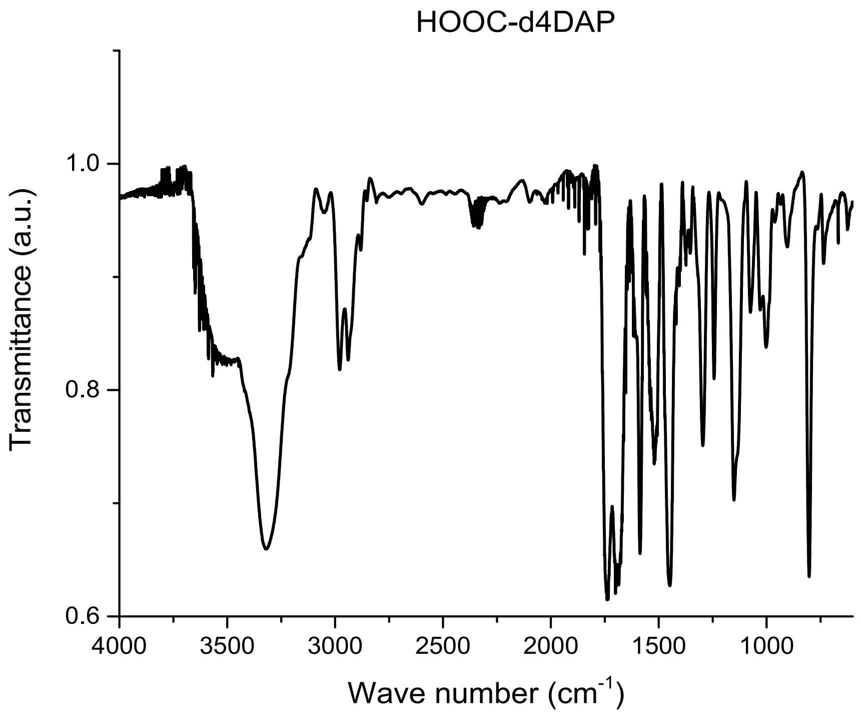

Figure S12. FTIR spectrum of **HOOC-d4DAP**.

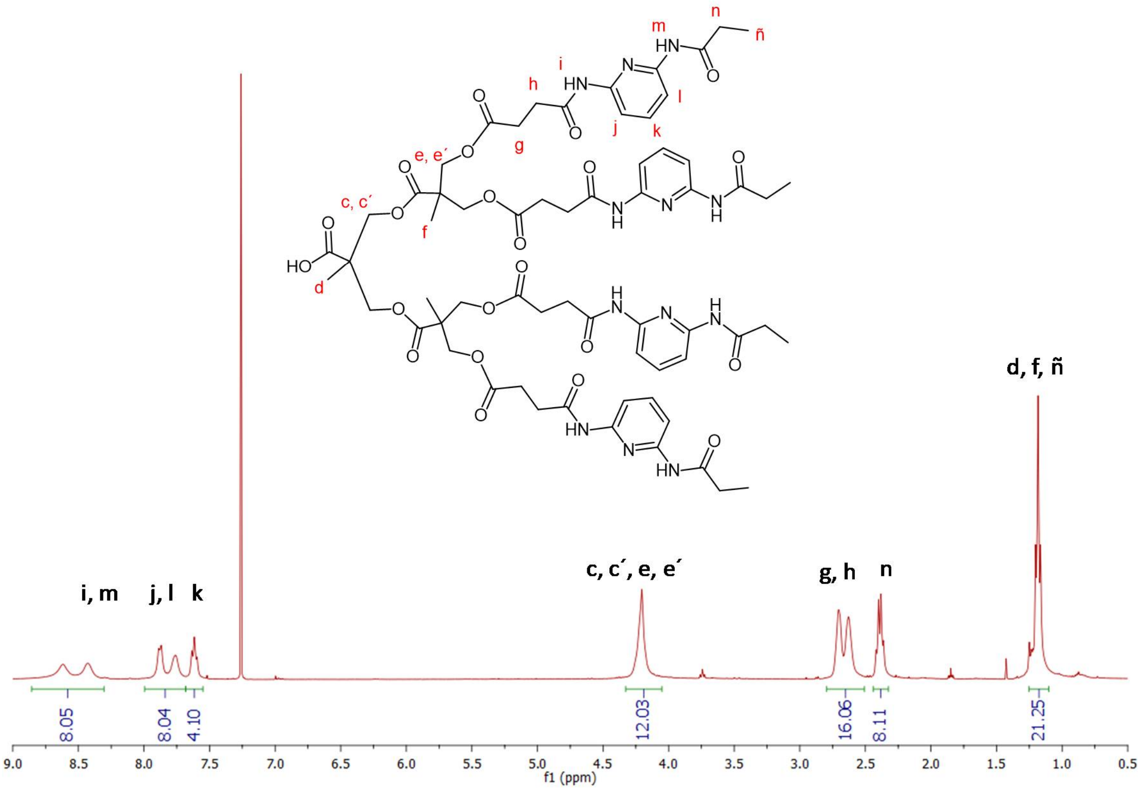

Figure S13. ^{1}H-NMR spectrum of **HOOC-d4DAP** (CDCl3, 400MHz) δ (ppm).

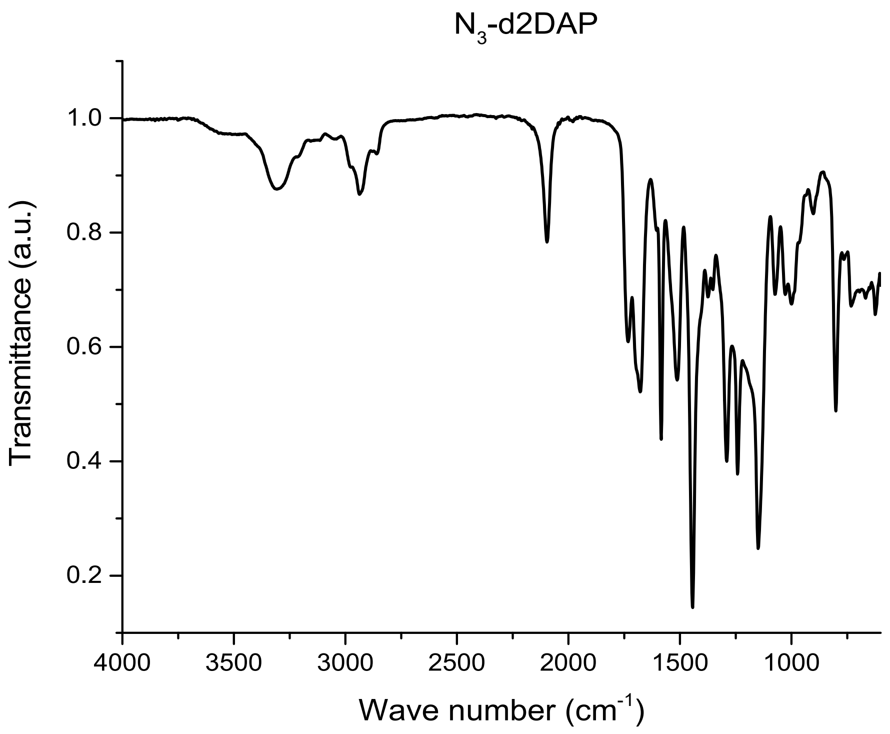

Figure S14. FTIR spectrum of **N₃-d2DAP**.

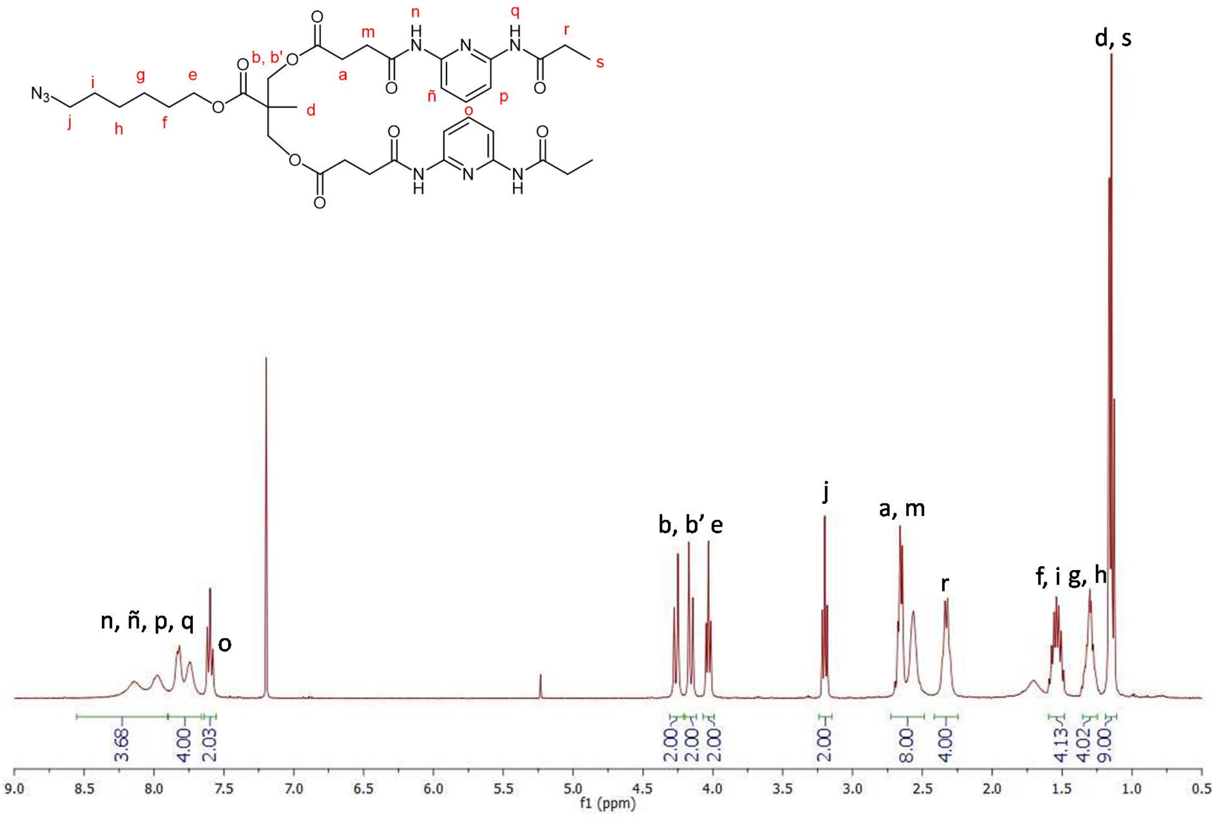

Figure S15. ^{1}H-NMR spectrum of **N_3-d2DAP** (CDCl3, 400MHz) δ (ppm).

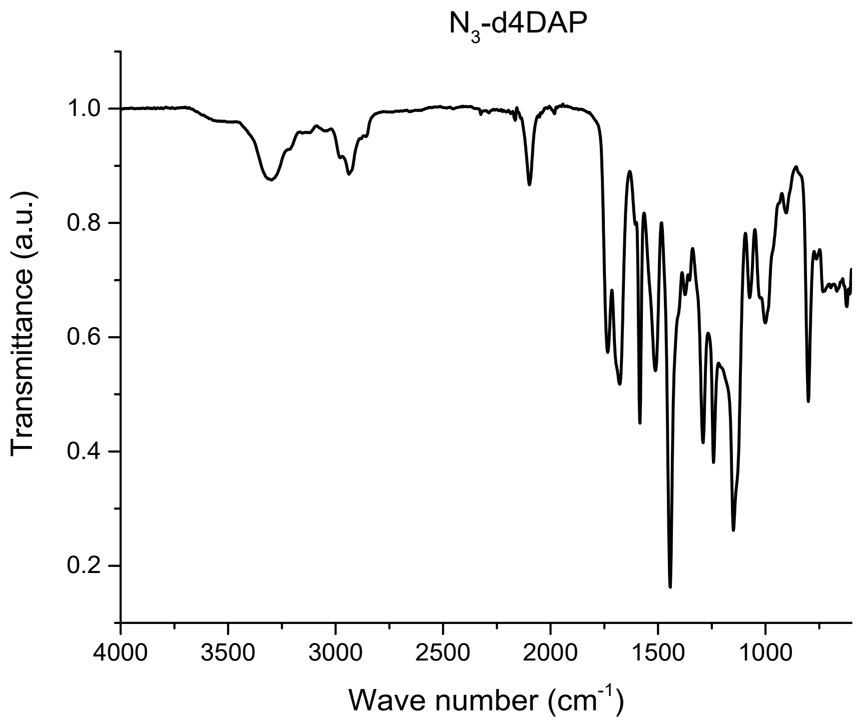

Figure S16. FTIR spectrum of **N_3-d4DAP**.

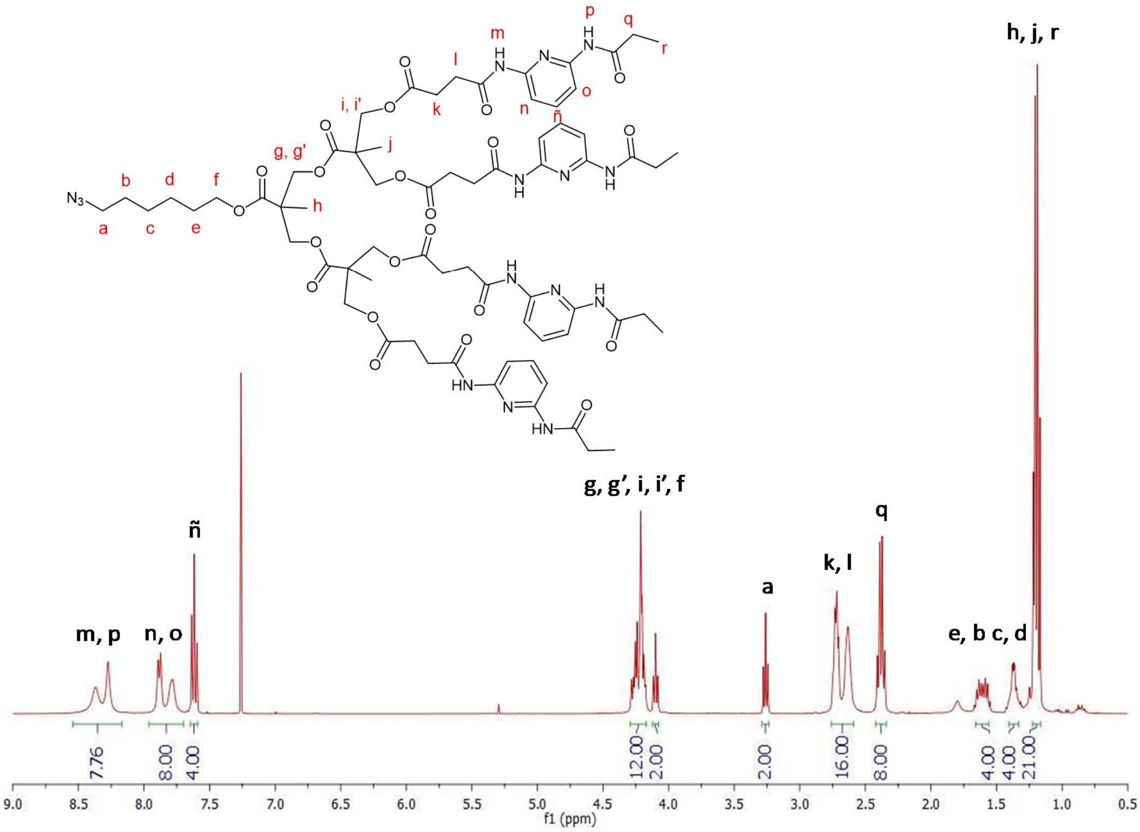

Figure S17. ^{1}H-NMR spectrum of **N_3-d4DAP** (CDCl3, 400MHz) δ (ppm).

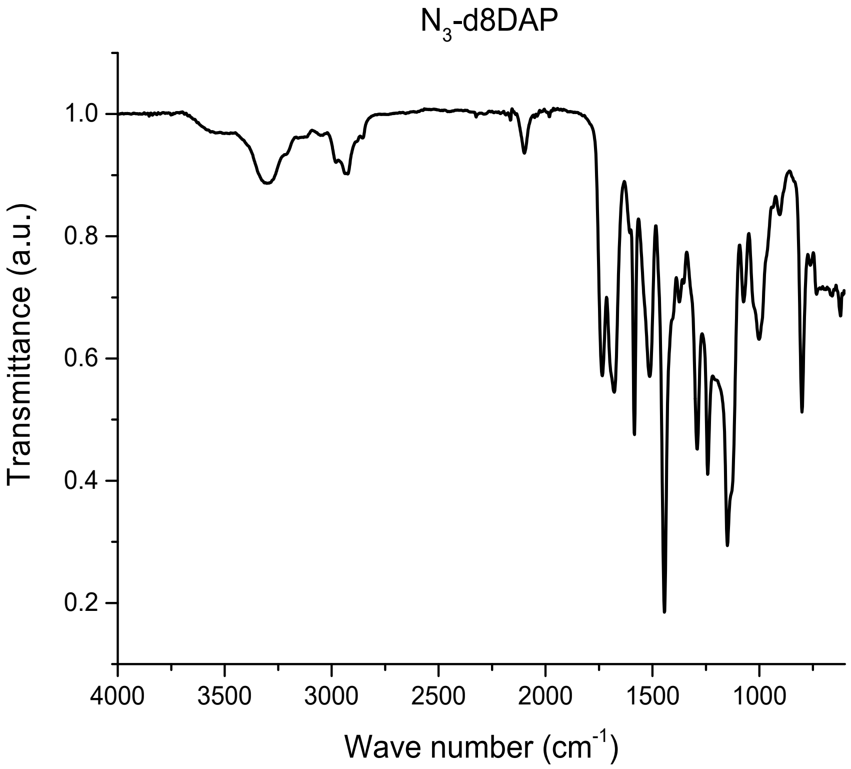

Figure S18. FTIR spectrum of **N3-d8DAP**.

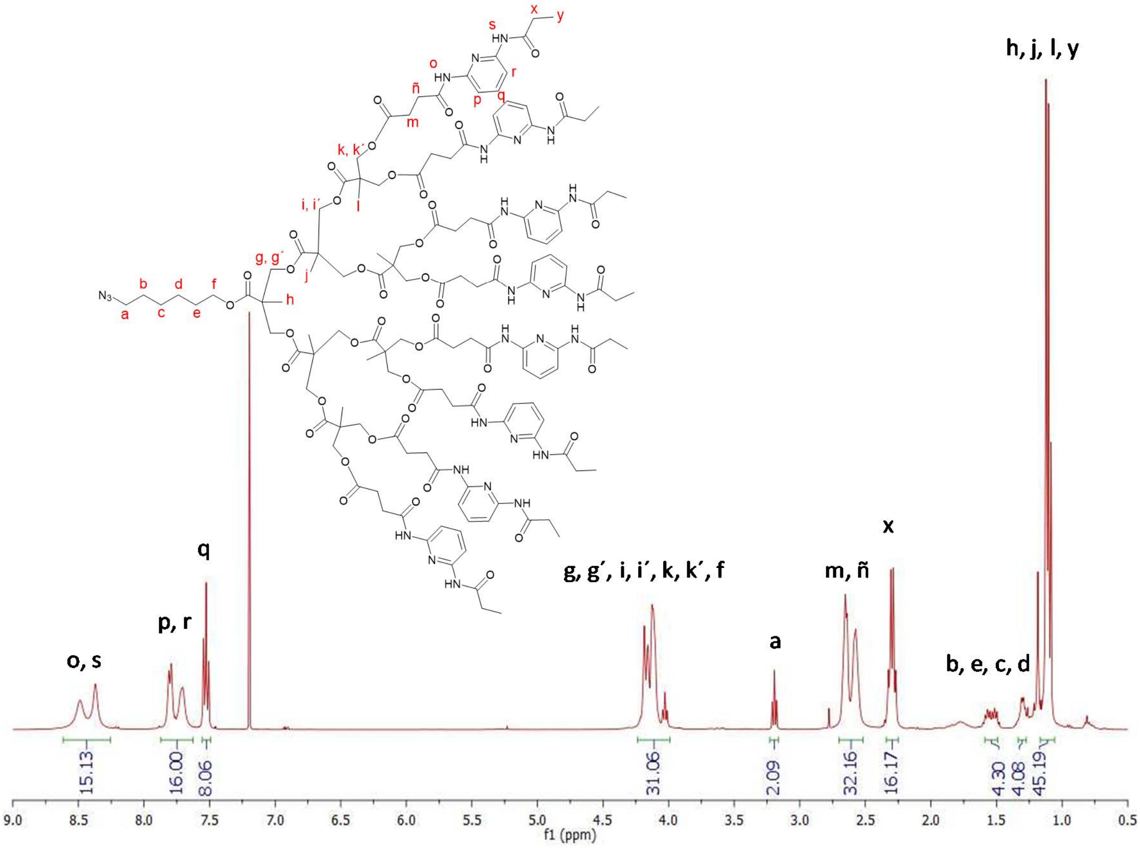

Figure S19. ^{1}H-NMR spectrum of **N3-d8DAP** (CDCl3, 400MHz) δ (ppm).

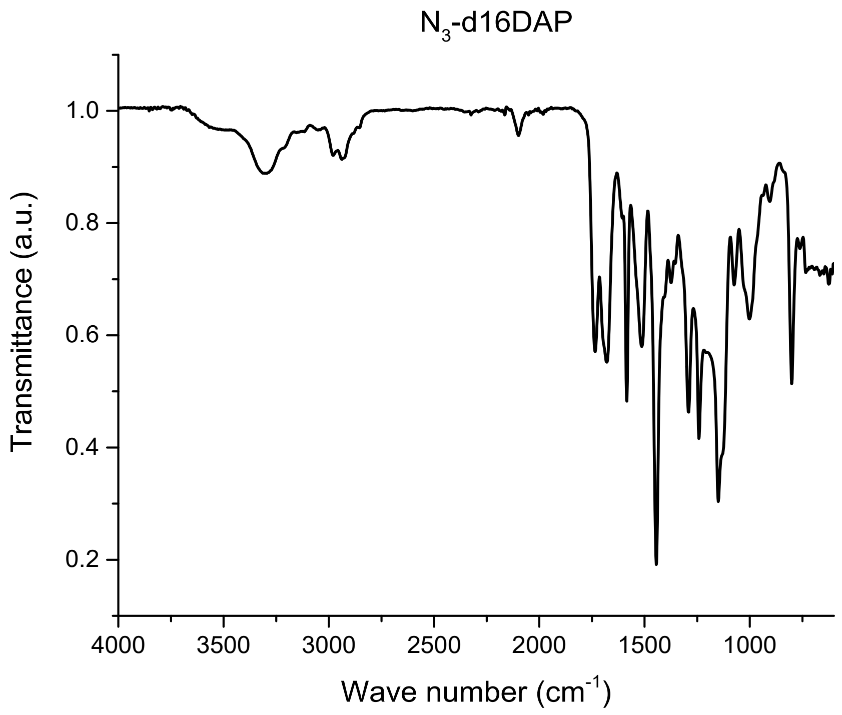

Figure S20. FTIR spectrum of **N3-d16DAP**.

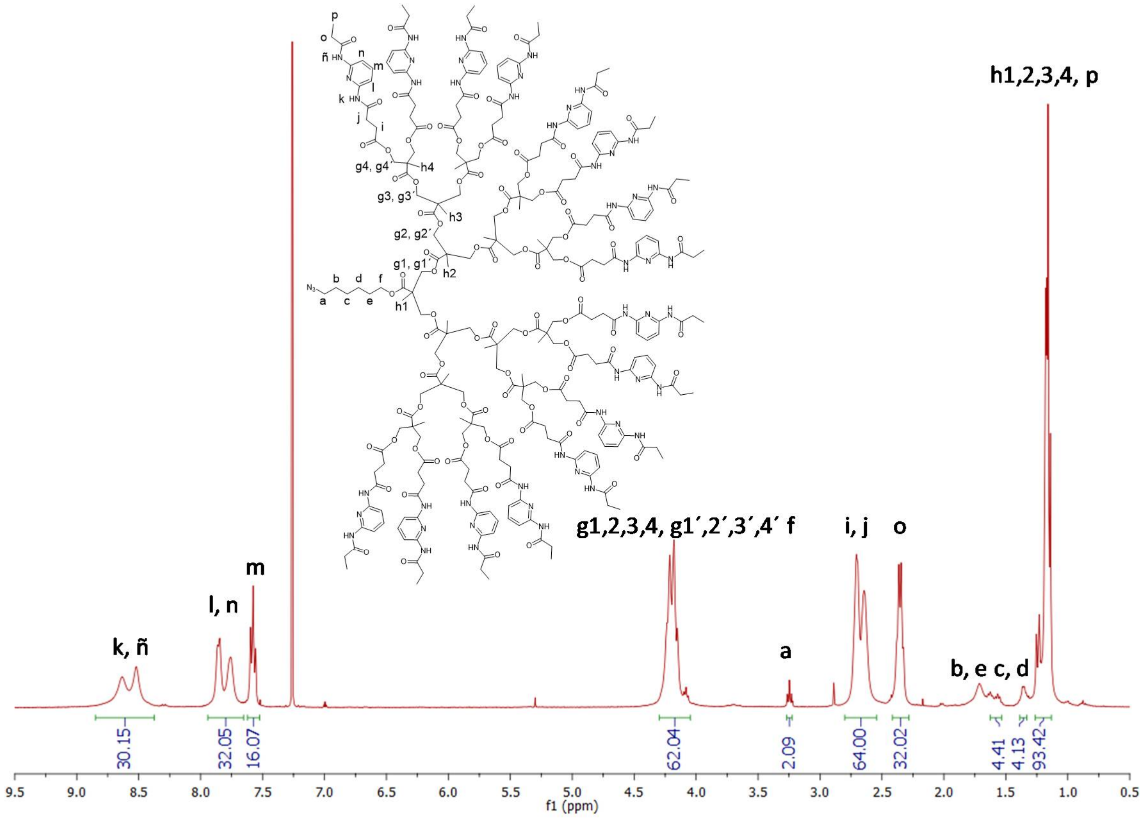

Figure S21. ^{1}H-NMR spectrum of **N3-d16DAP** (CDCl3, 400MHz) δ (ppm).

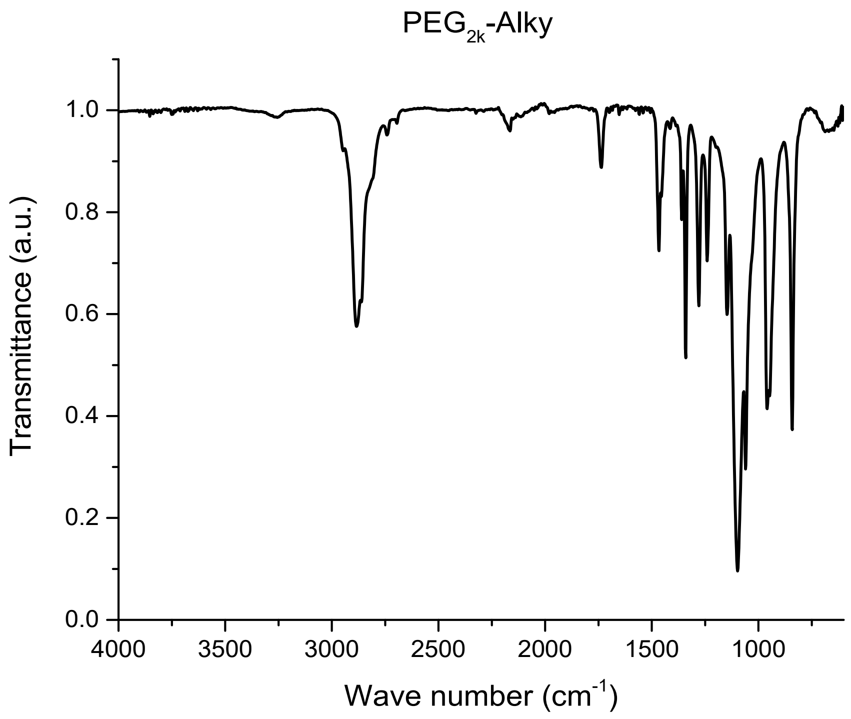

Figure S22. FTIR spectrum of **PEG2k-Alky**.

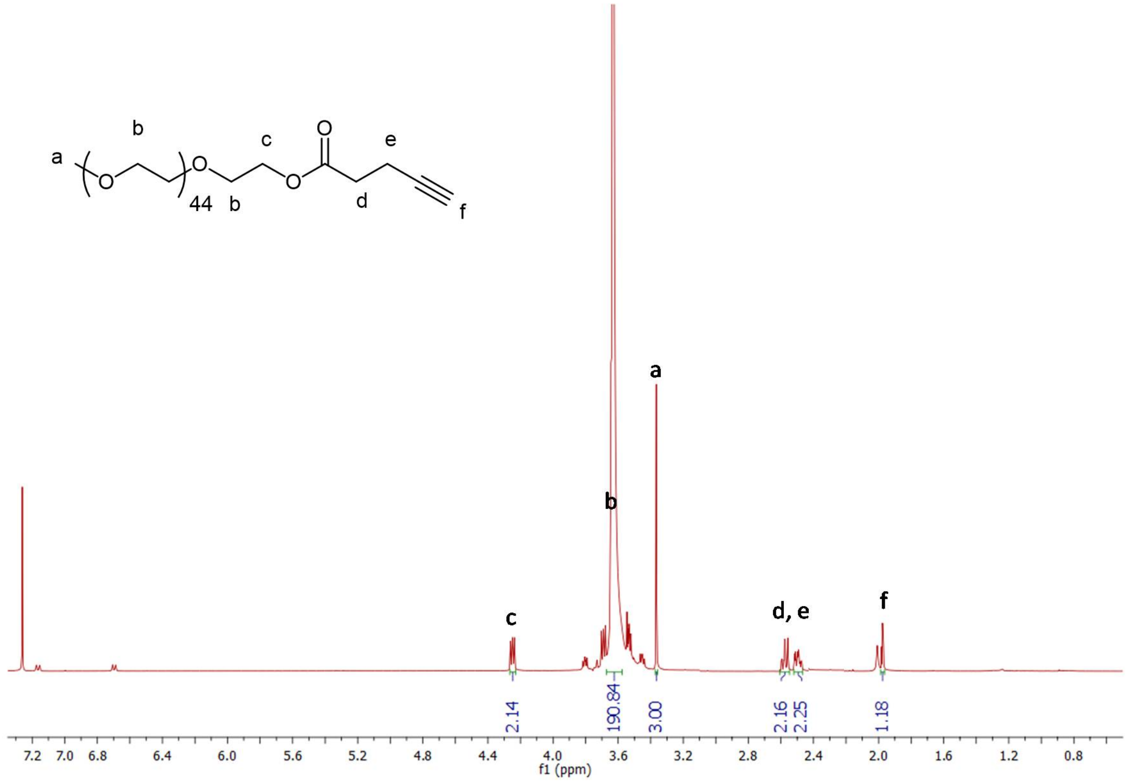

Figure S23. ^{1}H-NMR spectrum of **PEG2k-Alky** (CDCl3, 400MHz) δ (ppm).

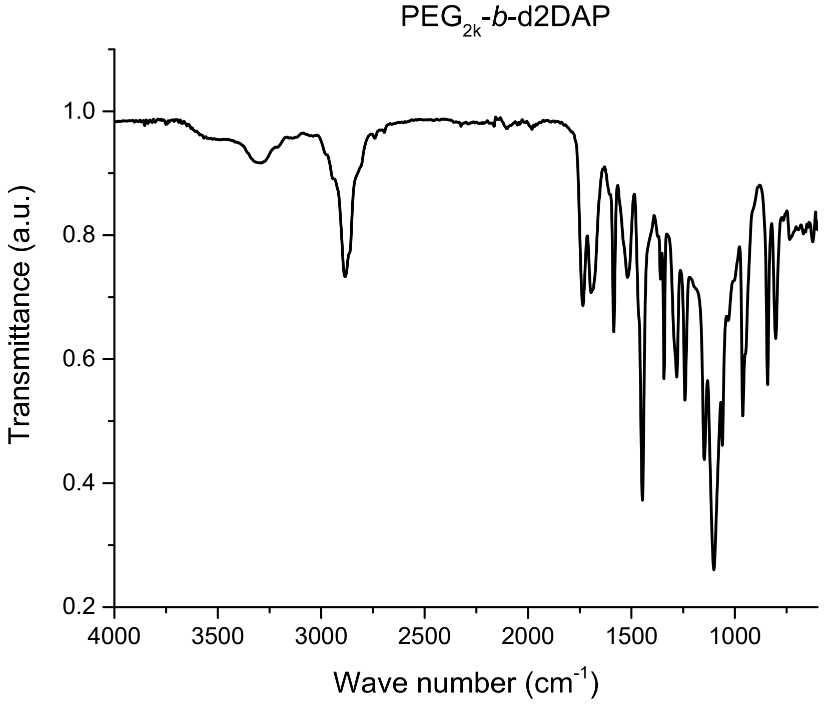

Figure S24. FTIR spectrum of **PEG2k-*b*-d2DAP**.

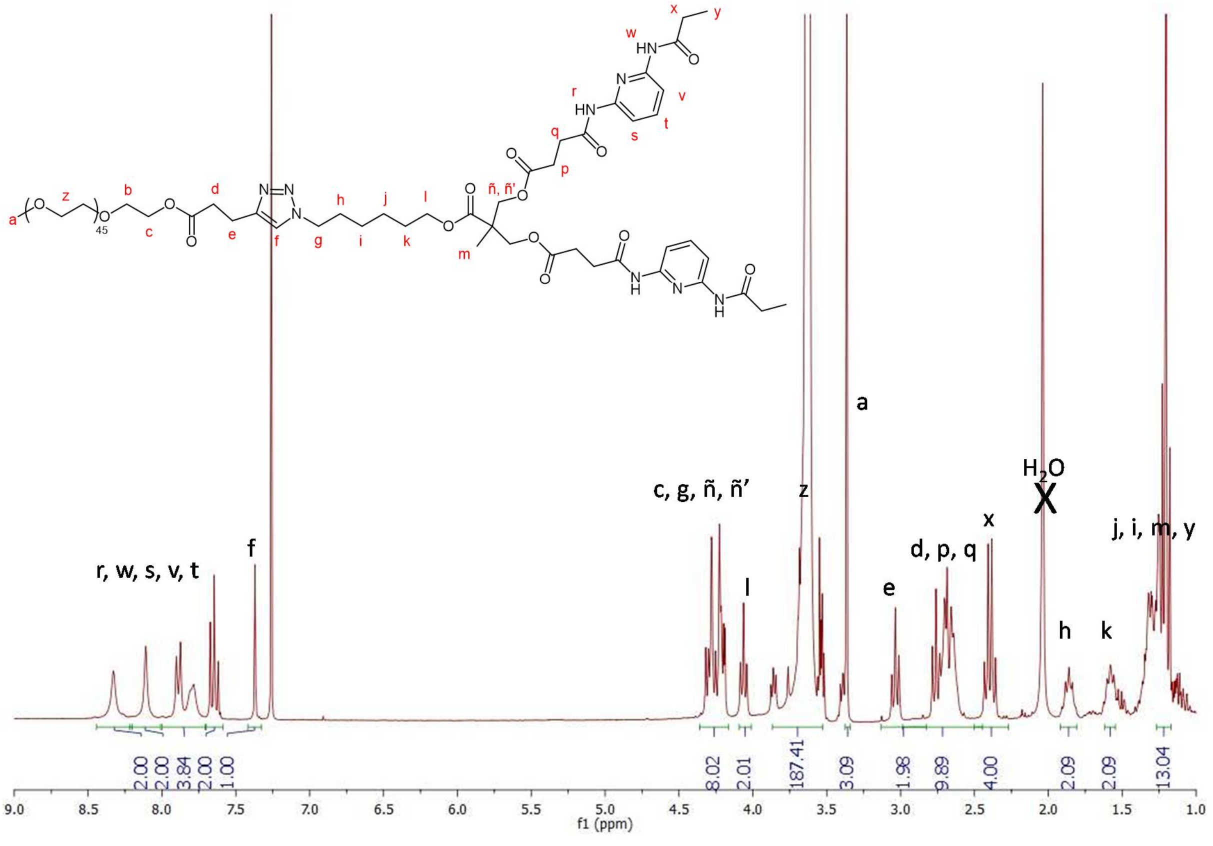

Figure S25. ^{1}H-NMR spectrum of **PEG2k-*b*-d2DAP** (CDCl3, 400MHz) δ (ppm).

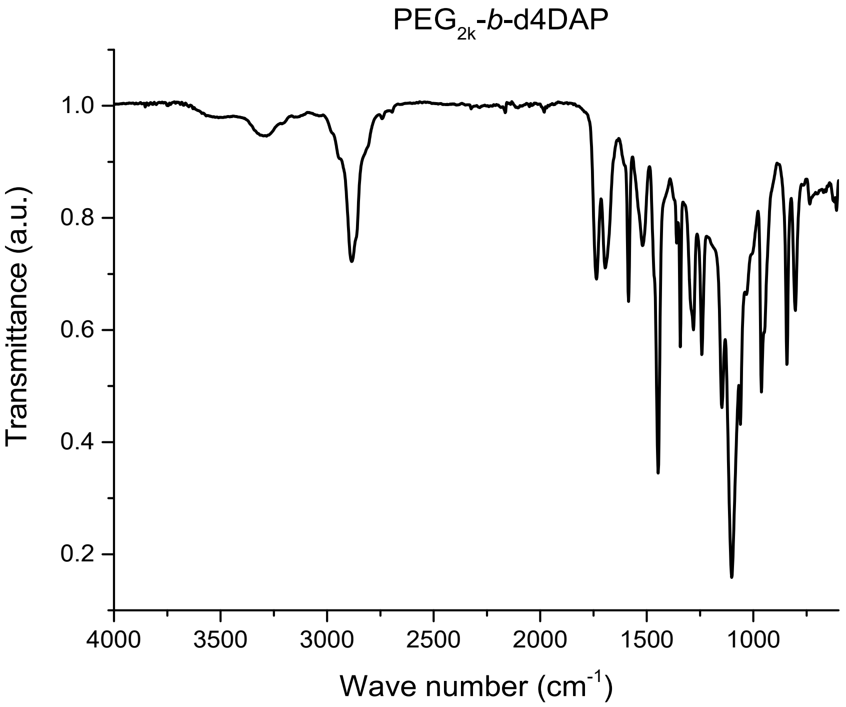

Figure S26. FTIR spectrum of **PEG2k-*b*-d4DAP**.

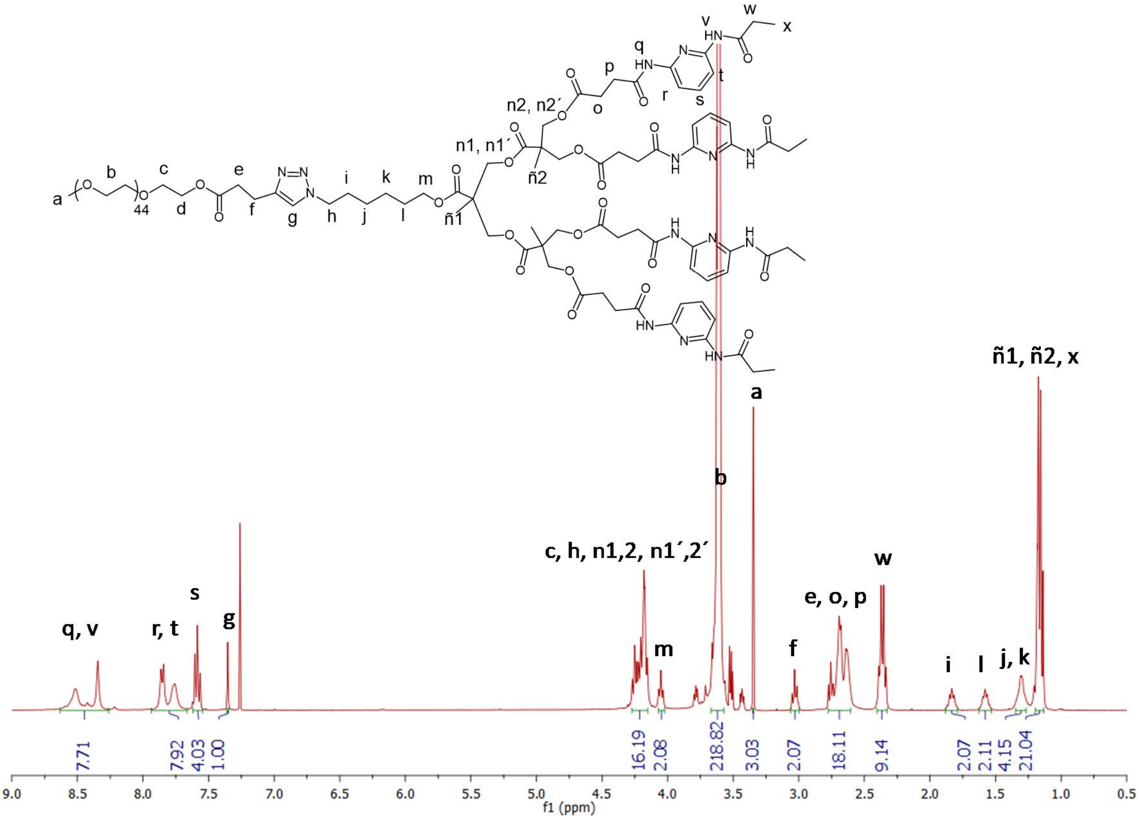

Figure S27. ¹H-NMR spectrum of **PEG2k-*b*-d4DAP** (CDCl3, 400MHz) δ (ppm).

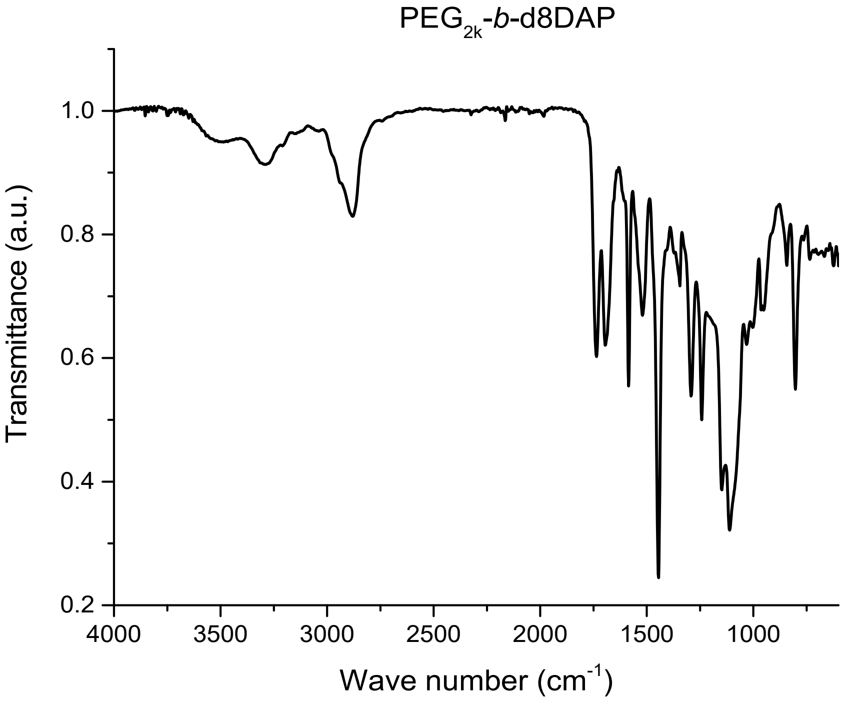

Figure S28. FTIR spectrum of **PEG2k-*b*-d8DAP**.

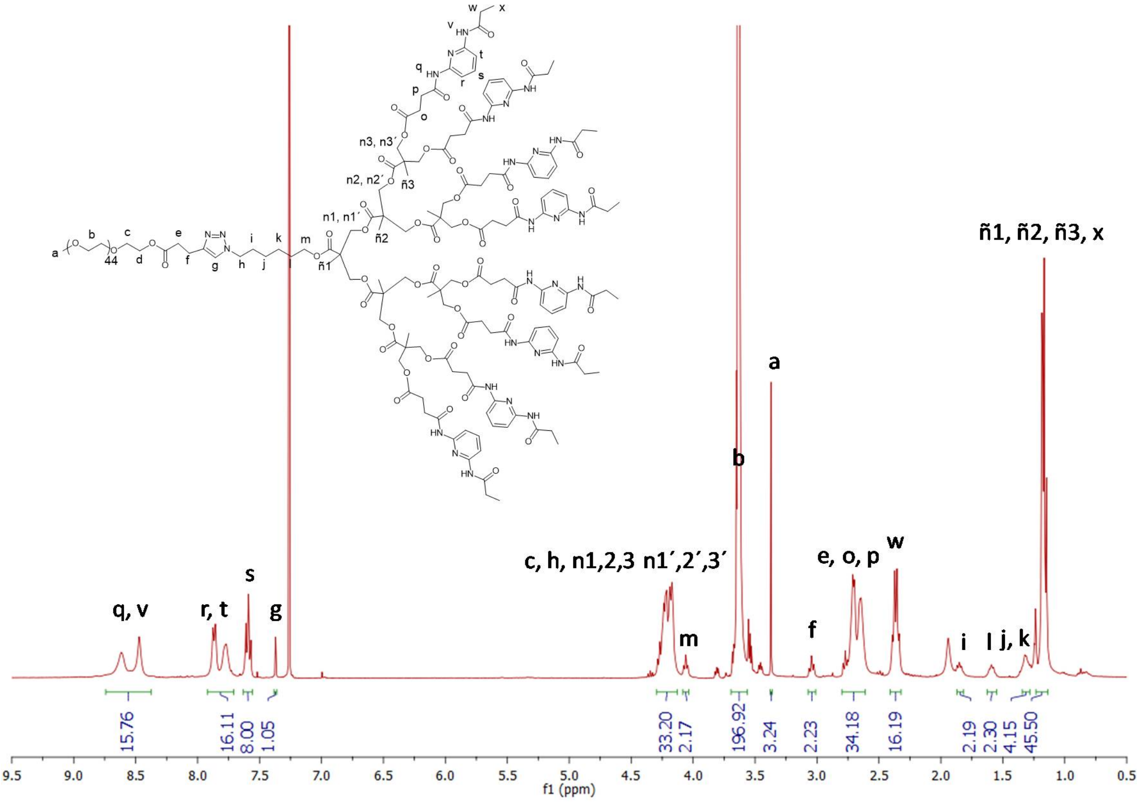

Figure S29. ^{1}H-NMR spectrum of **PEG2k-*b*-d8DAP** (CDCl3, 400MHz) δ (ppm).

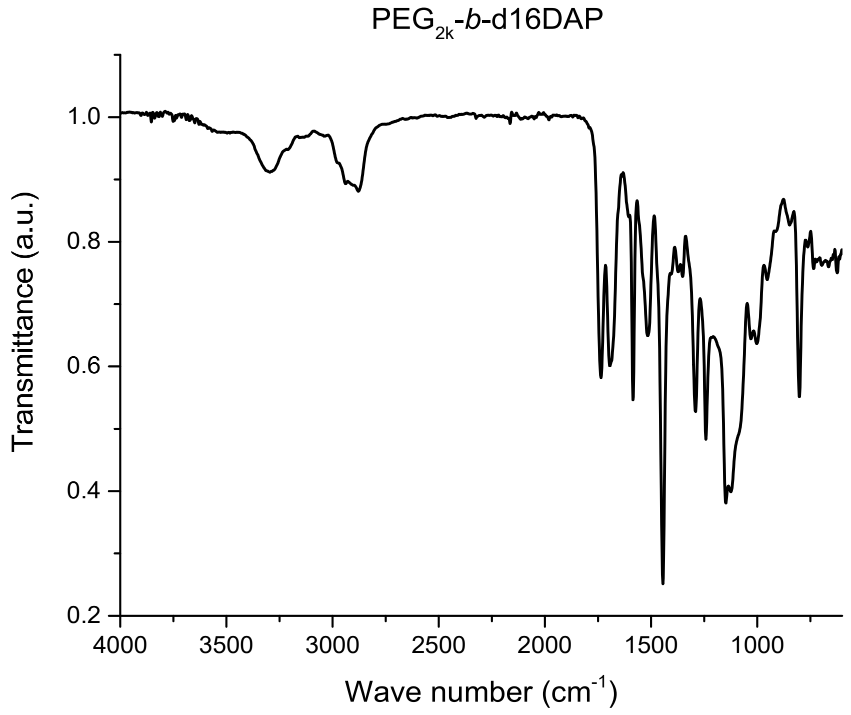

Figure S30. FTIR spectrum of **PEG2k-*b*-d16DAP**.

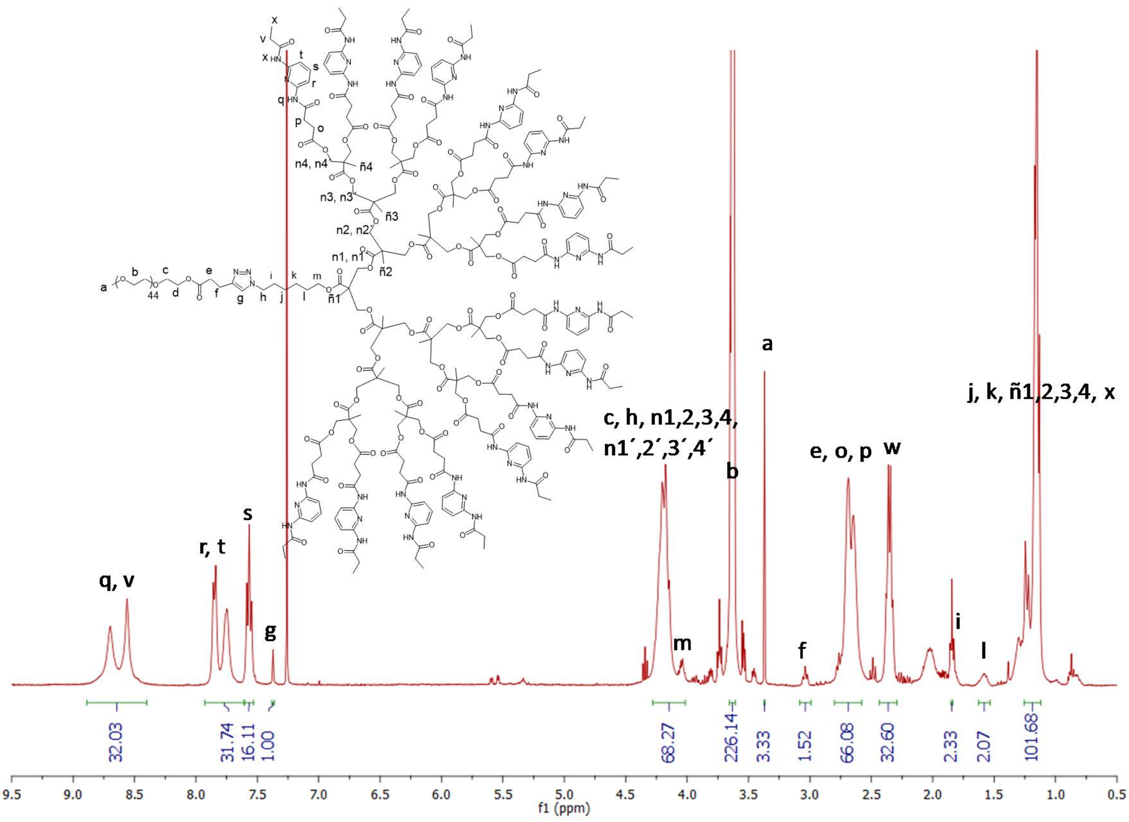

Figure S31. ^{1}H-NMR spectrum of **PEG2k-*b*-d16DAP** (CDCl3, 400MHz) δ (ppm).

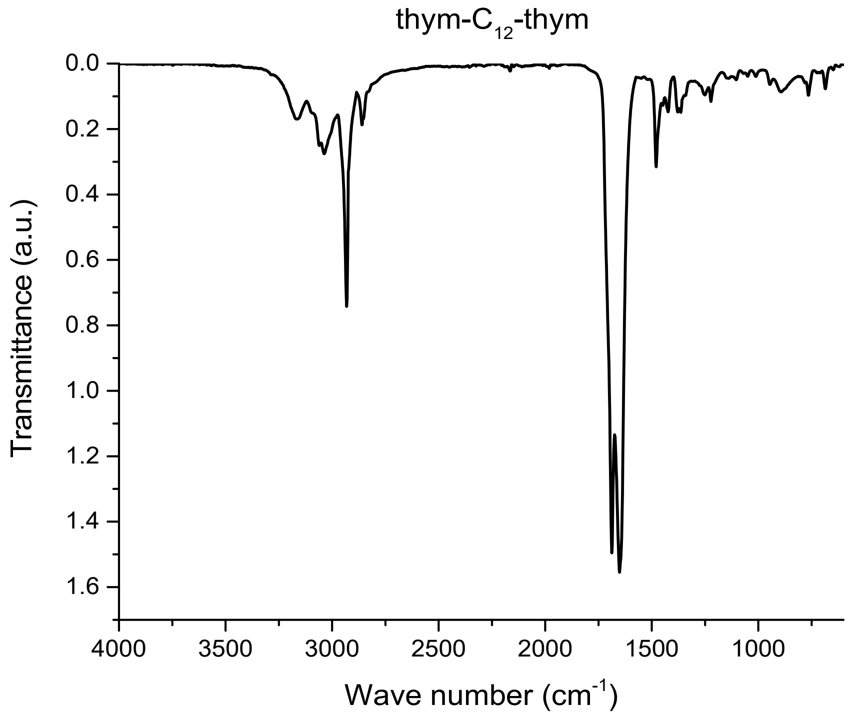

Figure S32. FTIR spectrum of **thym-C12-thym**.

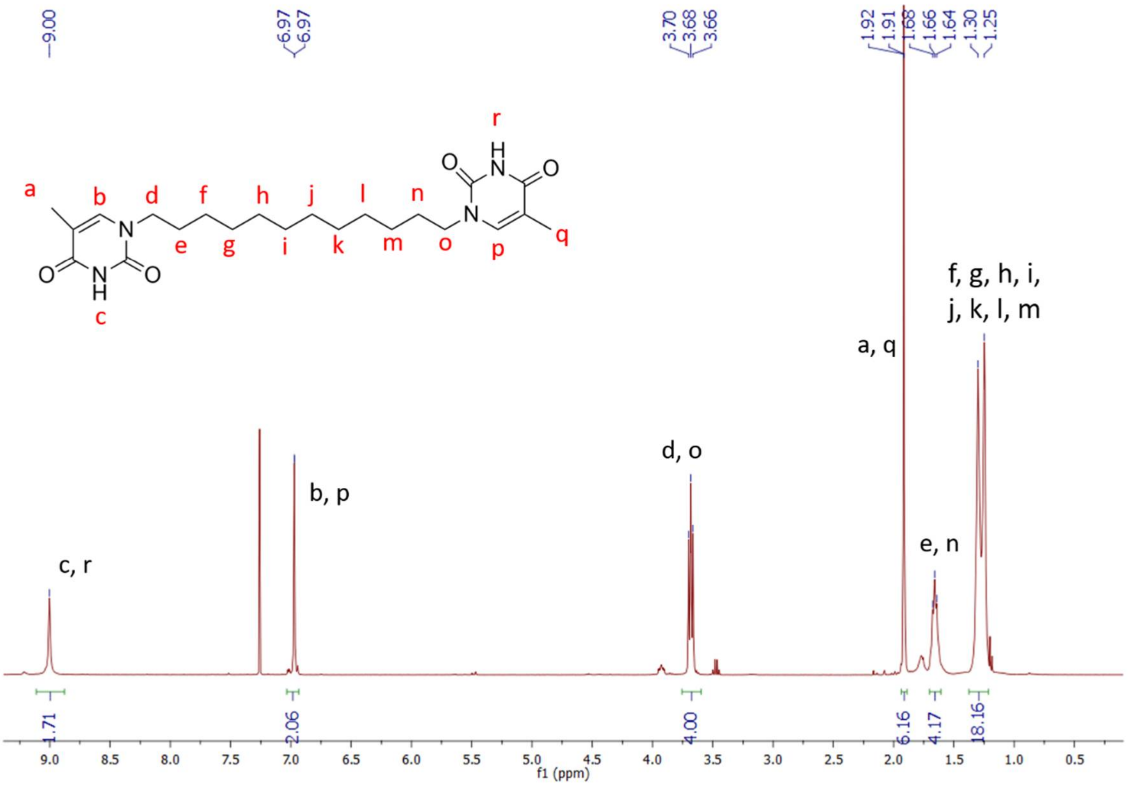

Figure S33. ^{1}H-NMR spectrum of **thym-C12-thym** (CDCl3, 400MHz) δ (ppm).

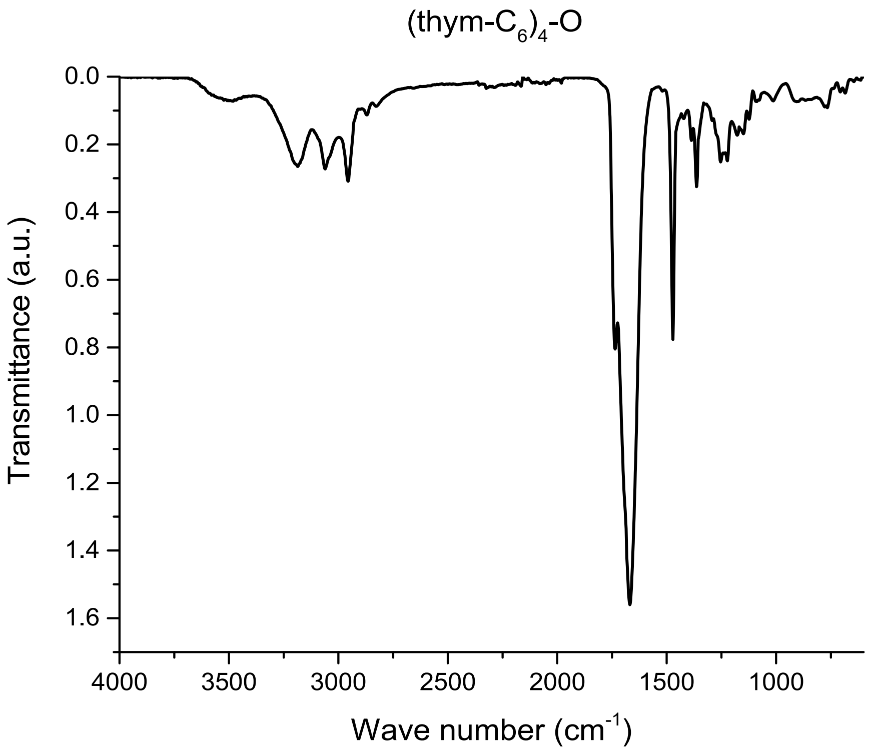

Figure S34. FTIR spectrum of **(thym-C$_6$)$_4$-O**.

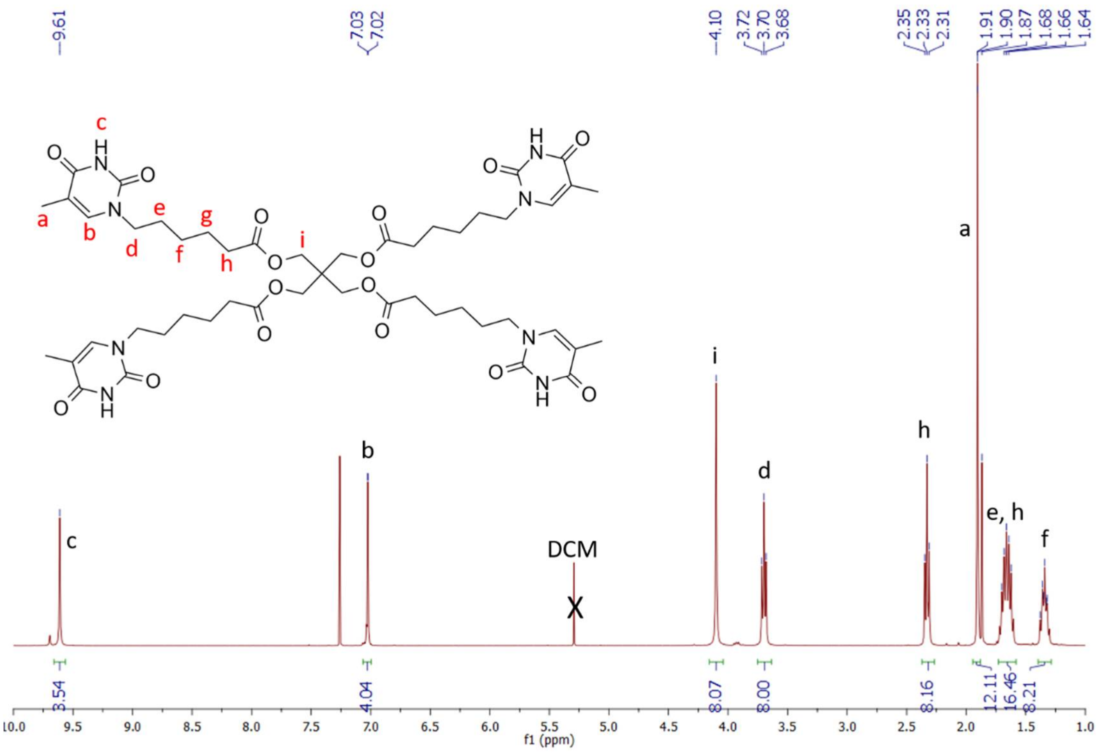

Figure S35. ^{1}H-NMR spectrum of **(thym-C$_6$)$_4$-O** (CDCl3, 400MHz) δ (ppm).

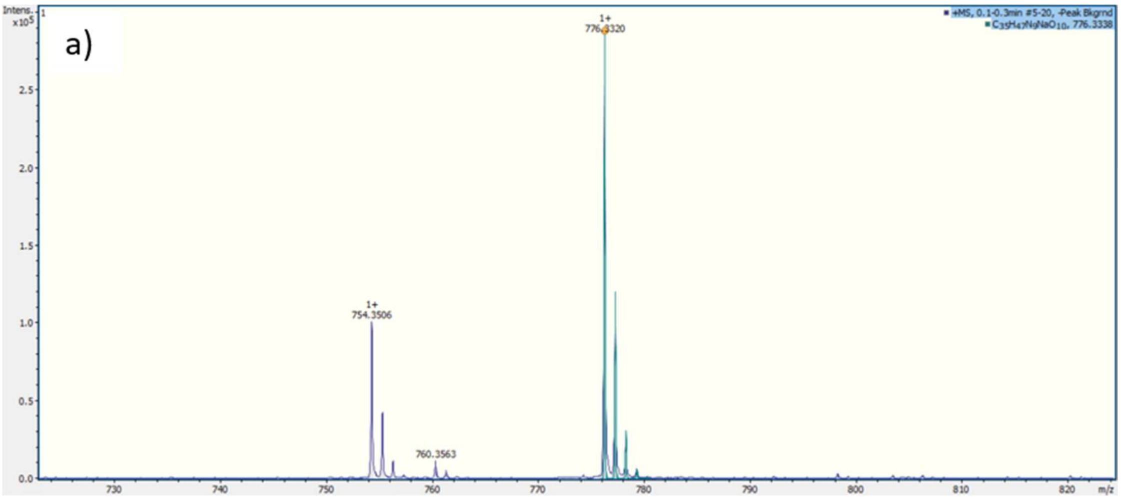

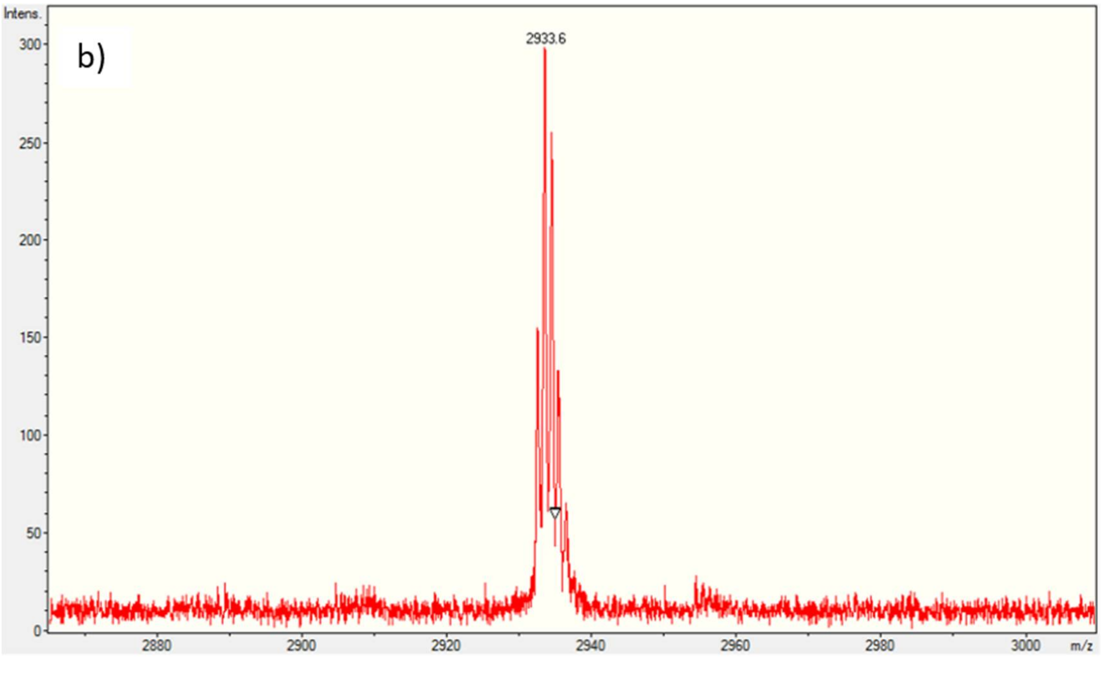

Figure S36. a) HR-ESI spectrum of **N_3-d2DAP**. b) MALDI-TOF spectra of **N_3-d4DAP**.

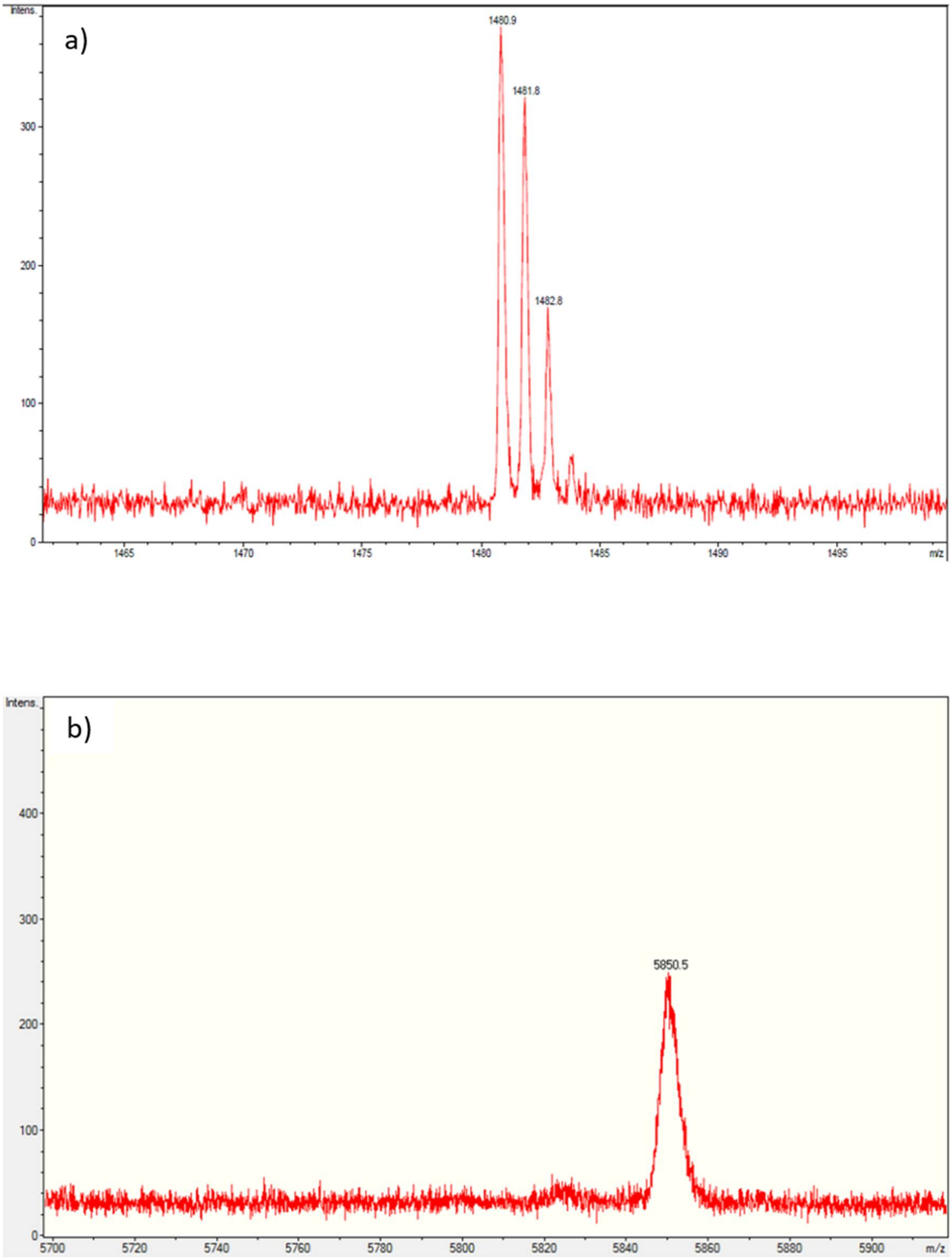

Figure S37. MALDI-TOF spectra of a) **N_3-d8DAP** and b) **N_3-d16DAP**.

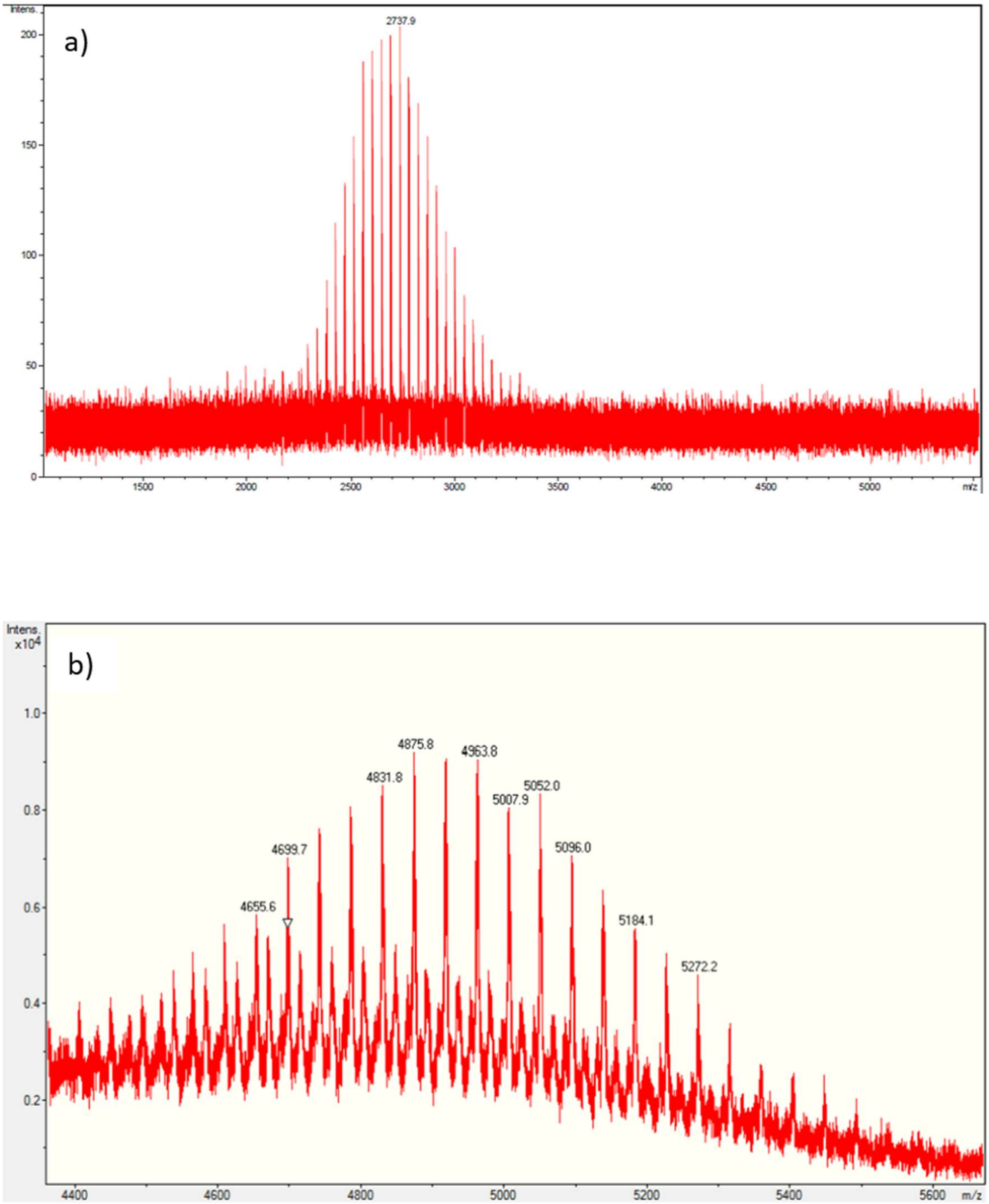

Figure S38. MALDI-TOF spectra of a) **PEG$_{2k}$-*b*-d2DAP** and b) **PEG$_{2k}$-*b*-d4DAP.**

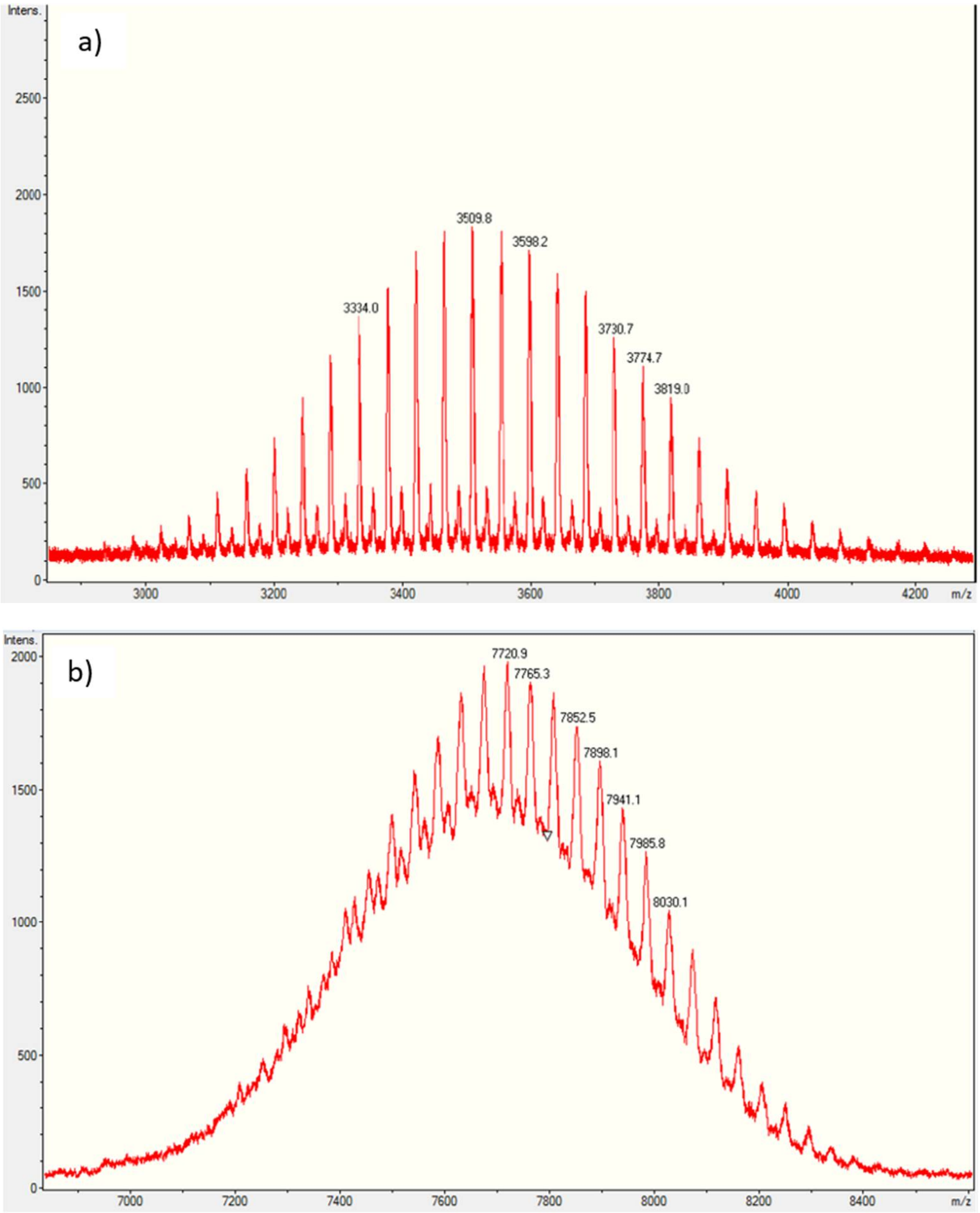

Figure S39. MALDI-TOF spectra of a) **PEG$_{2k}$-*b*-d8DAP** and b) **PEG$_{2k}$-*b*-d16DAP.**

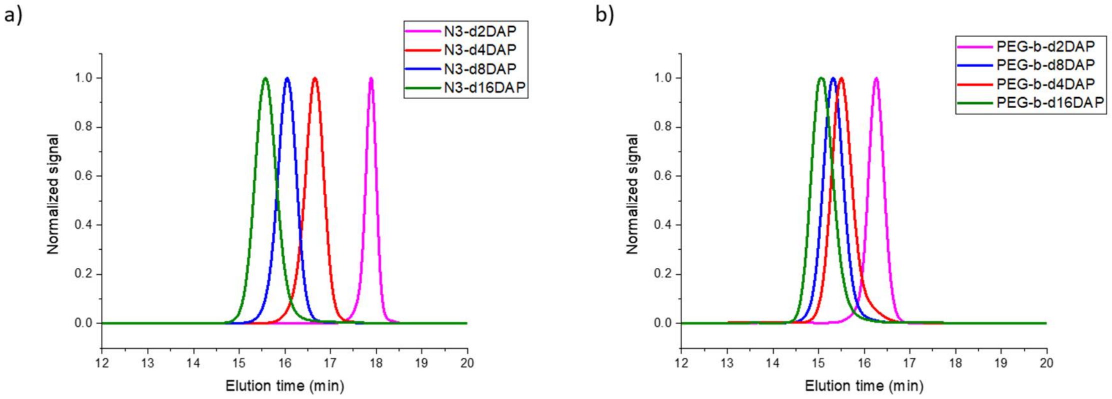

Figure S40. GPC chromatograms for a) dendrons **N$_3$-dxDAP** and b) LDBCs **PEG$_{2k}$-*b*-dxDAP.**

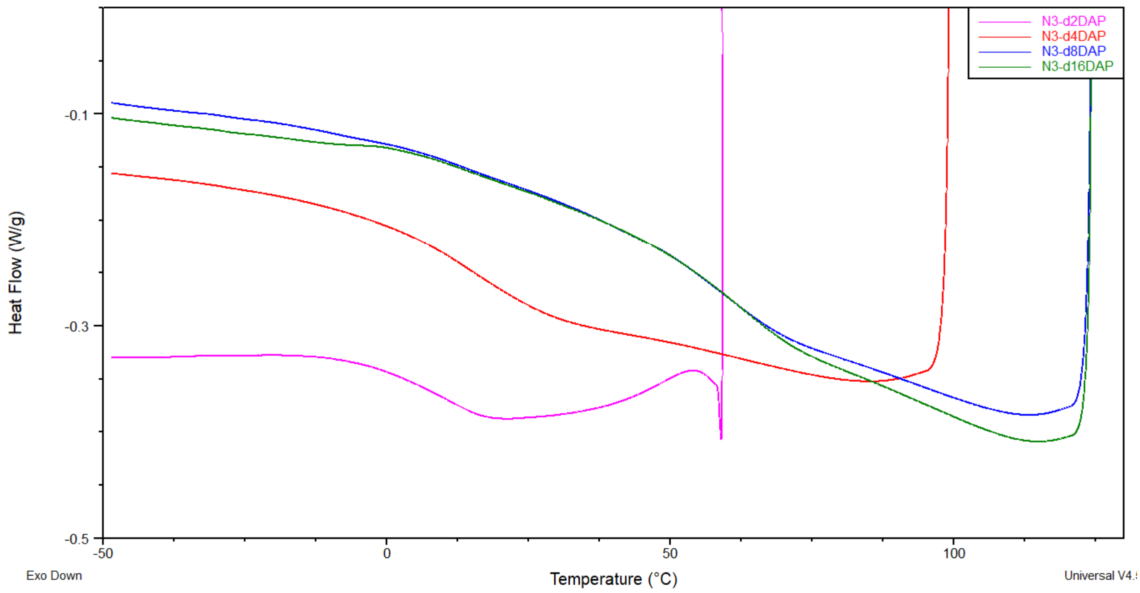

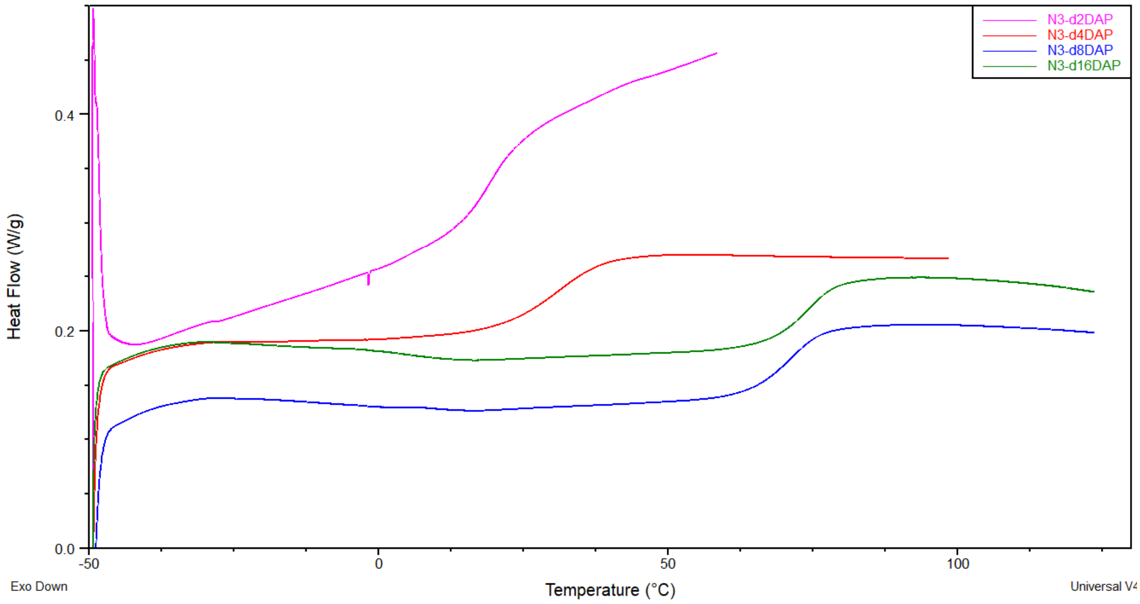

Figure S41. DSC curves registered on first cooling (above) and second heating (below) at 10 °C min^{-1} scanning rate of **N$_3$-dxDAP**.

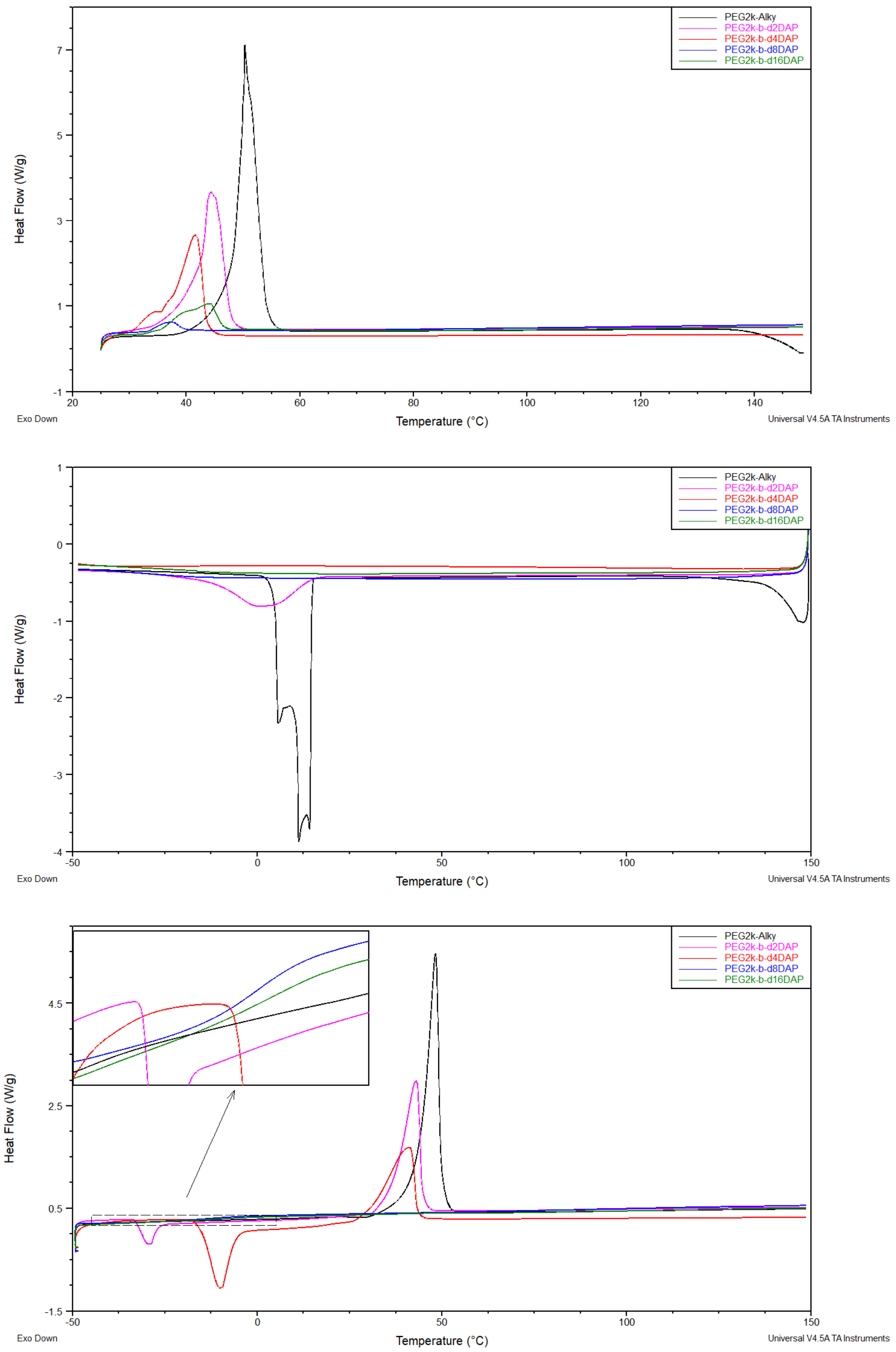

Figure S42. DSC curves registered on first heating (above), first cooling (middle) and second heating (below) at 10 °C min^{-1} scanning rate of **PEG$_{2k}$-*b*-dxDAP**.

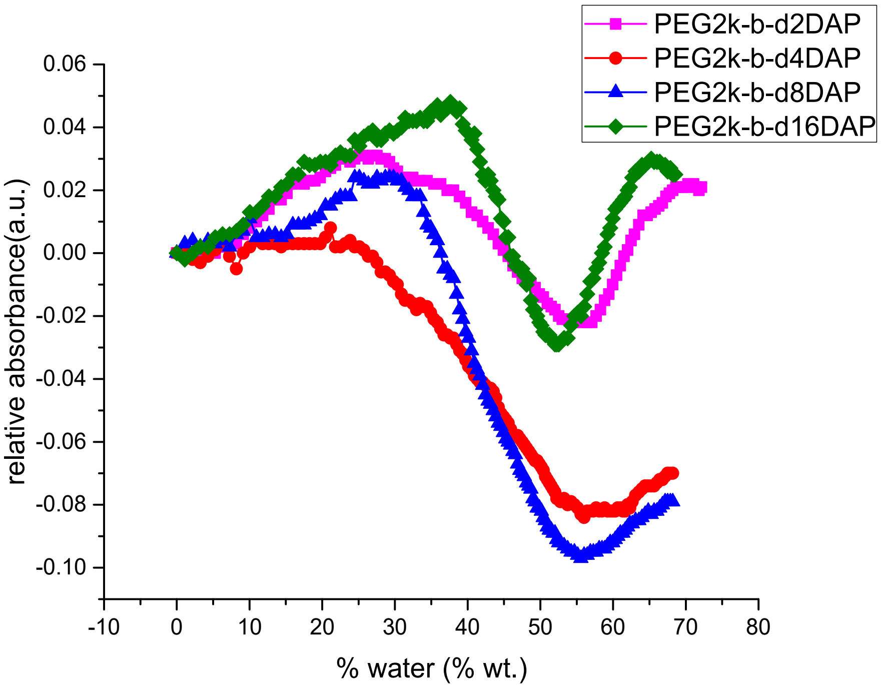

Figure S43. Turbidity curves of LDBCs **PEG2k-*b*-dxDAP**.

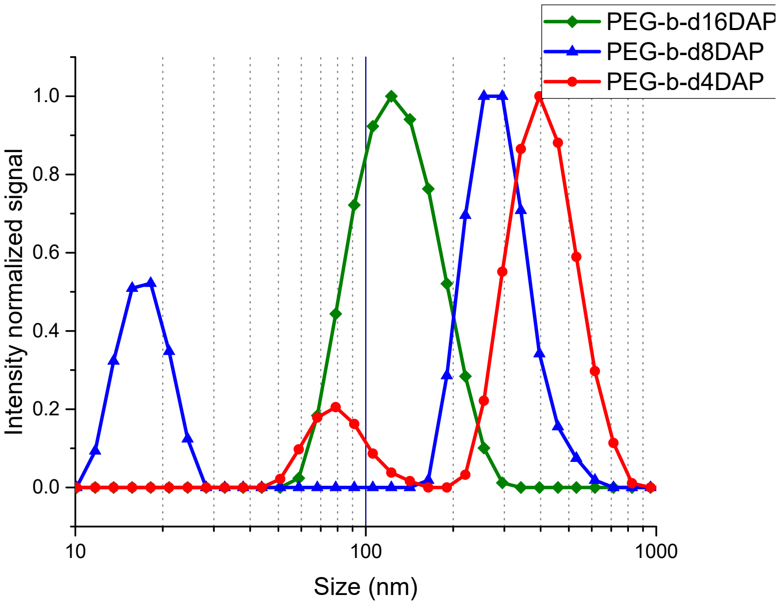

Figure S44. Intensity size distributions by DLS of LDBCs self-assemblies prepared by nanoprecipitation.

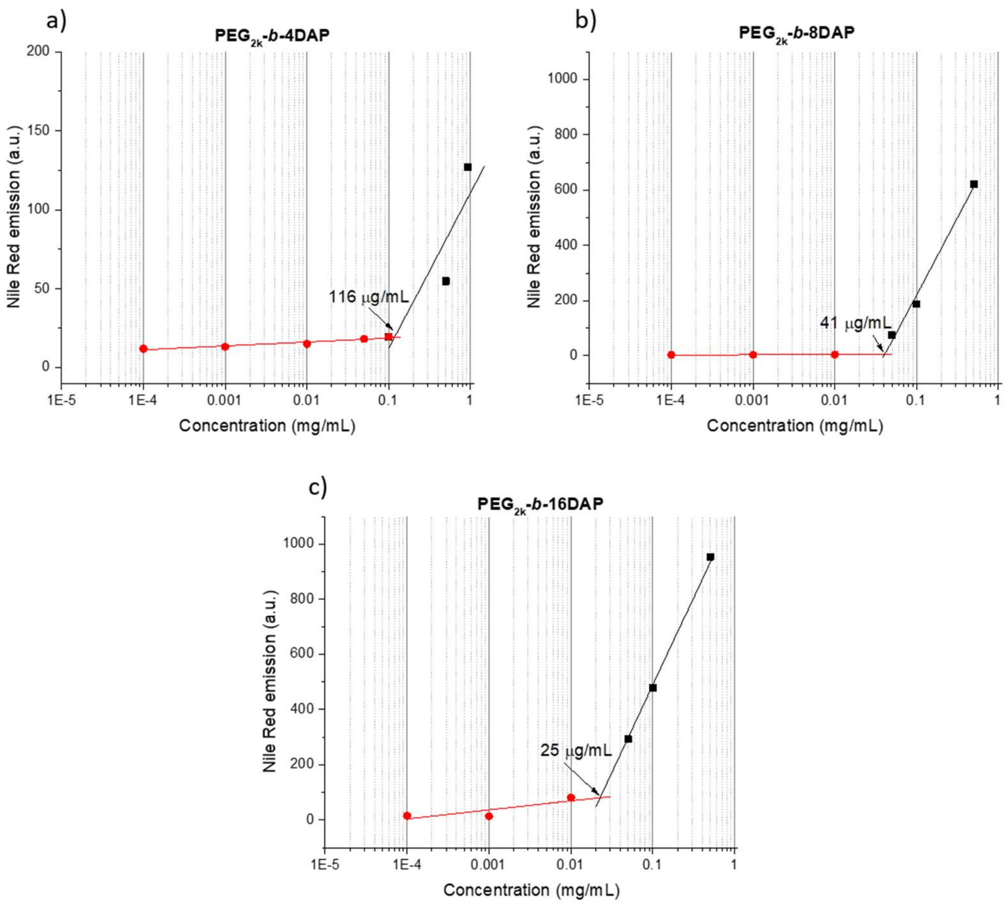

Figure S45. Fluorescence emission of Nile Red at 620 nm (λ_{exc} = 550 nm) versus a) **PEG2k-*b*-d4DAP,** b) **PEG2k-*b*-d8DAP** and c) **PEG2k-*b*-d16DAP** concentration. CAC was determined from the intersection of the two extrapolated lines.

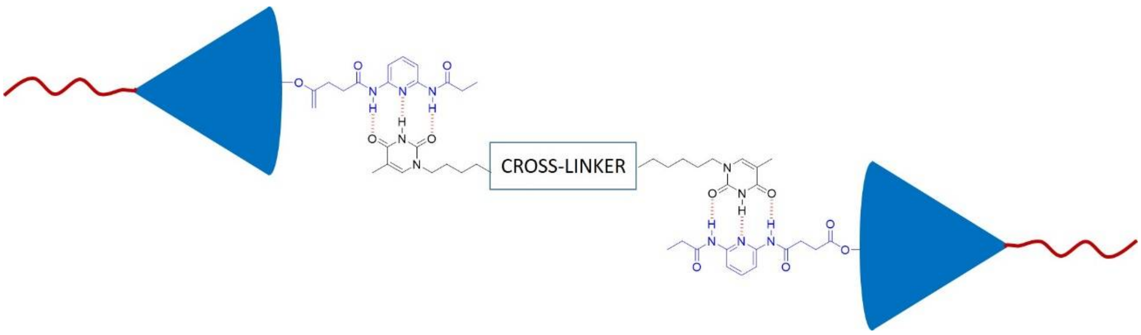

Scheme S1. Supramolecular recognition through a triple H-bond between DAP units (blue) linked at the periphery of dendron block of **PEG2k-*b*-dxDAP** and thymine moieties (black) of the crosslinkers.

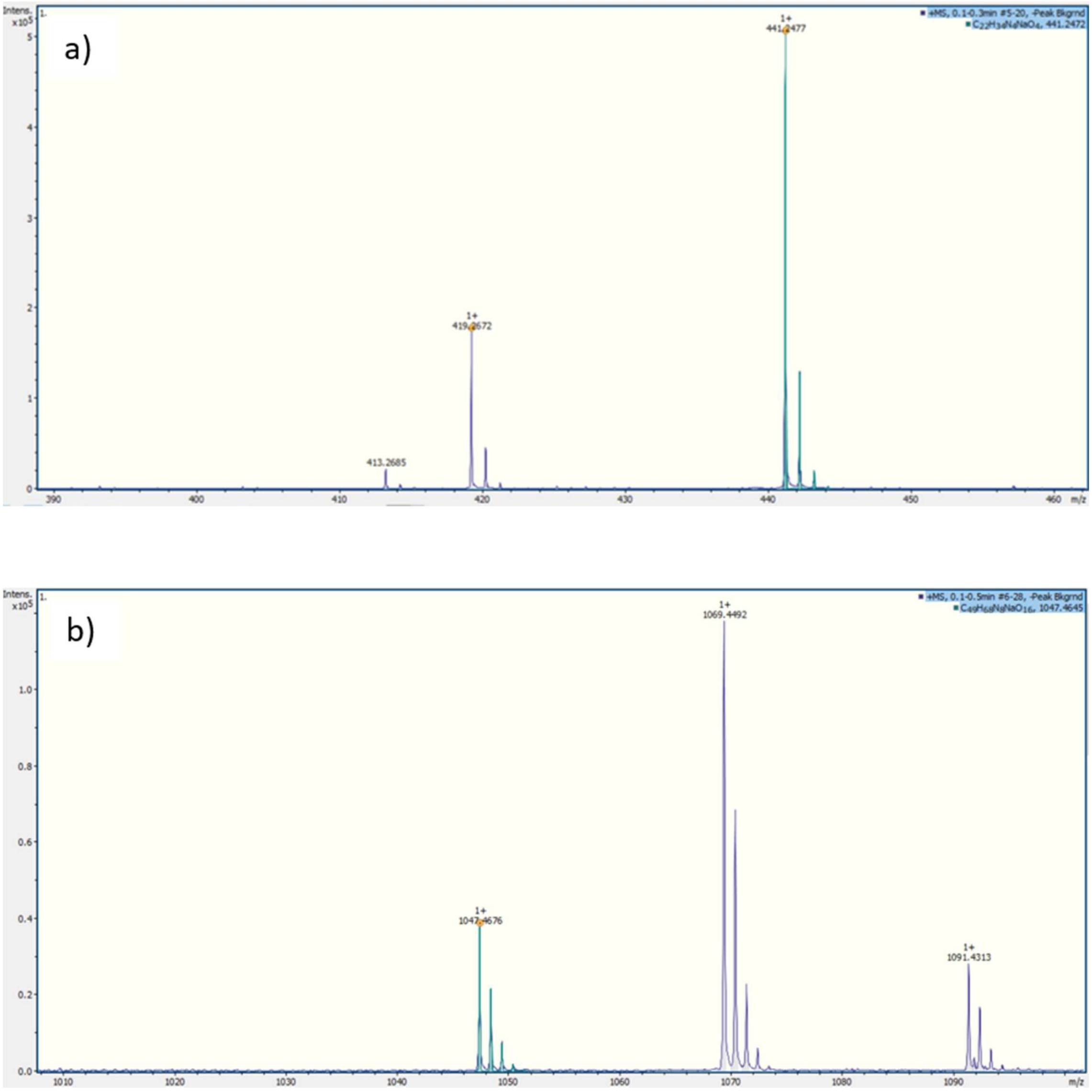

Figure S46. HR-ESI spectra of a) **thym-C$_{12}$-thym** and b) **(thym-C$_6$)$_4$-O**.

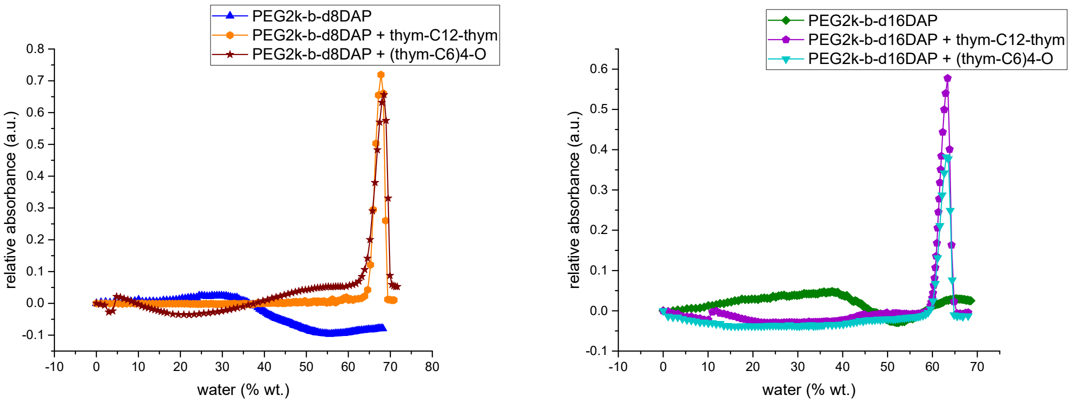

Figure S47. Turbidity curves of supramolecular cross-linked LDBCs of **PEG$_{2k}$-*b*-d8DAP** (left) and **PEG$_{2k}$-*b*-d16DAP** (right).

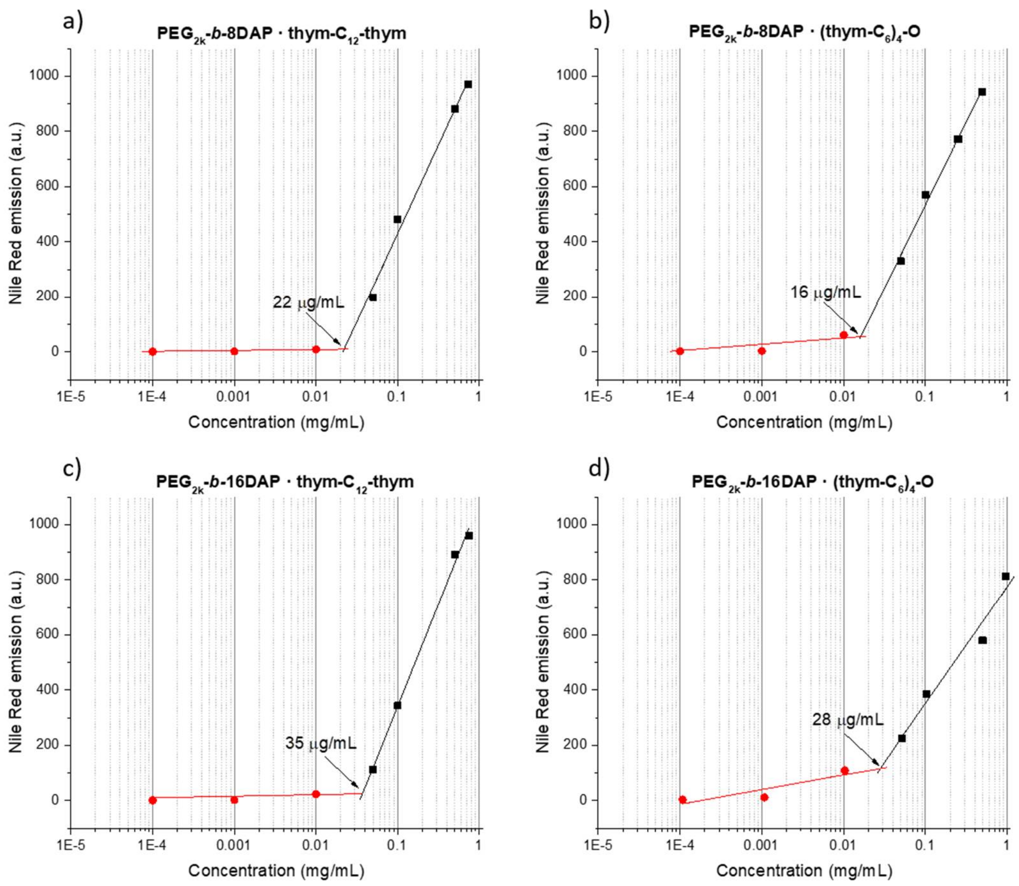

Figure S48. Fluorescence emission of Nile Red at 620 nm (λ_{exc} = 550 nm) versus a) **PEG$_{2k}$-*b*-d8DAP·thym-C$_{12}$-thym**, b) **PEG$_{2k}$-*b*-d8DAP·(thym-C$_6$)$_4$-O**, c) **PEG$_{2k}$-*b*-d16DAP·thym-C$_{12}$-thym** and **PEG$_{2k}$-*b*-d16DAP·(thym-C$_6$)$_4$-O** concentration. CAC was determined from the intersection of the two extrapolated lines.
